# One-pot synthesis of 2-arylated and 2-alkylated benzoxazoles and benzimidazoles based on triphenylbismuth dichloride-promoted desulfurization of thioamides

**DOI:** 10.3762/bjoc.18.155

**Published:** 2022-10-18

**Authors:** Arisu Koyanagi, Yuki Murata, Shiori Hayakawa, Mio Matsumura, Shuji Yasuike

**Affiliations:** 1 School of Pharmaceutical Sciences, Aichi Gakuin University, 1-100 Kusumoto-cho, Chikusa-ku, Nagoya 464-8650, Japan

**Keywords:** benzazole, bismuth, cyclization, desulfurization, thioamide

## Abstract

The development of novel and efficient synthesis methods for 2-substituted benzazole derivatives is of interest as they are biologically active substances. Herein, a simple method for the synthesis of 2-aryl- and 2-alkyl-substituted benzazoles is described. The reaction of 2-aminophenols with thioamides at 60 °C in the presence of triphenylbismuth dichloride in 1,2-dichloroethane as a promoter afforded various 2-aryl- and 2-alkylbenzoxazoles in moderate to excellent yields under mild reaction conditions. This method could also be applied to the synthesis of benzimidazoles and benzothiazoles. This study presents the first use of triphenylbismuth dichloride to produce benzimidoyl chloride from thioamides by desulfurization and chlorination, as well as its application to the synthesis of 2-substituted benzazoles.

## Introduction

In the production of pharmacologically active compounds, 2-substituted benzazoles containing oxazole and imidazole skeletons are the most commonly used scaffolds [1–5]. Therefore, it is crucial for the drug industry to develop methods for their syntheses [1–2,4,6]. For example, conventional benzoxazole synthesis methods involve the condensation of 2-aminophenol with various carbonyl compounds, such as carboxylic acid derivatives or aldehydes [6–9]. These classic reactions require harsh conditions, such as using strong acids or high temperatures. The syntheses of azoles using one-pot reactions are attracting increasing attention as alternatives to conventional methods; a notable alternative is the cyclodesulfurization, where intramolecular cyclization is combined with desulfurization from thioamide analogs such as thioureas, thiosemicarbazides, and dithiocarbamate salts under mild reaction conditions [10]. These synthesis methods are effective for constructing azoles with an amine functional group at the 2-position, such as benzoxazole or benzoimidazole, but are unsuitable for the syntheses of 2-arylated or 2-alkylated benzoxazoles and benzimidazoles. In fact, only two reports cover the subject. Jackson et al. carried out the intramolecular ring-closure reaction of *o*-alkoxythiobenzamides with iodine in the presence of sodium hydride as the base [11]. Sugita et al. reported an unstable iodoalkyne, pentafluoro(iodoethynyl)benzene, which catalyzed the cyclization of thioamides with 2-aminophenol [12]. These approaches have some drawbacks, such as low yields, the need for bases, and limited substrate scope.

**Scheme 1:**
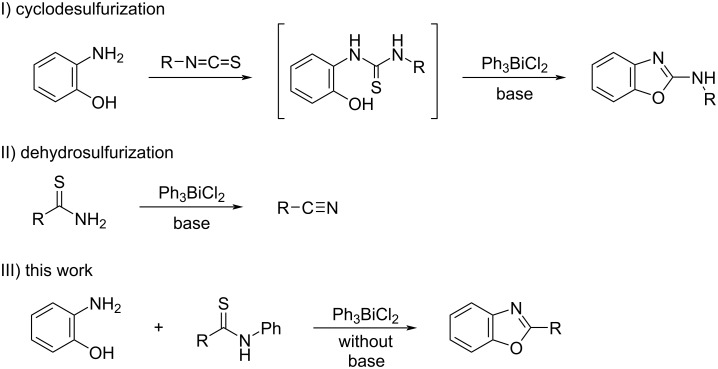
Utilization of Ph_3_BiCl_2_ for organic reactions involving desulfurization.

With the development of organobismuth chemistry, these compounds have been applied in various areas of science including biology and organic synthesis because they are normally non-toxic and exhibit unique biological activities [13–21]. Among them, triarylbismuth dichlorides (Ar_3_BiCl_2_) have been widely used as aryl group donors for the *C*- and *O*-arylation of phenol derivatives [22–24], *N*-arylation of pyridin-2-ones [25–26], α-arylation of α,β-unsaturated carbonyls [27–28], and tandem 1,3-bisarylation of cyclopropanes with arenes [29]. They have also been used in Pd-catalyzed cross-coupling reactions to react with hypervalent iodonium salts, organostananes, and vinyl epoxides [30–32]. Moreover, there are reports of them serving as oxidizing agents for alcohols [33]. Two papers have recently reported triphenylbismuth dichloride (Ph_3_BiCl_2_) to act efficiently in desulfurization reactions (Scheme 1-I and II). In 2018, we reported the preparation of 2-aminobenzoxazoles by the Ph_3_BiCl_2_-mediated cyclodesulfurization of thioureas, which were obtained from 2-aminophenols and isothiocyanates [34]. In 2019, Doris et al. performed the dehydrosulfurization of thioamides and thioureas that provided nitriles and cyanamides [35]. Using bismuth compounds as desulfurization reagents for synthesizing heteroazoles, this paper presents the syntheses of 2-aryl- and 2-alkylbenzoxazoles through the ring-closure reaction of 2-aminophenol with benzimidoyl chloride, which is produced by the desulfurization and chlorination of thioamides promoted by Ph_3_BiCl_2_ without a base. The developed protocol is also applied to prepare 2-substituted benzimidazoles using *N*-tosyl-1,2-phenylenediamines as substrates.

## Results and Discussion

We initially focused on identifying the optimal experimental conditions for the synthesis of 2-phenylbenzoxazole (**8a**) using 2-aminophenol (**1a**) with benzothioamides **2**–**5** in the presence of organobismuth or organoantimony compounds **6** or **7**. The results, including those for the screening of suitable ring-closure reagents, solvents, thioamides, and reagent ratios, are summarized in Table 1. We first performed the reaction of **1a** (0.5 mmol) with *N*-phenylbenzothioamide (**2a**, 1.0 mmol) using organobismuth or organoantimony reagents **6** or **7** (1.0 mmol) in 1,2-dichloroethane (DCE) under aerobic conditions at 60 °C for 18 h (Table 1, entries 1–8). The use of Ph_3_BiCl_2_ **6a** resulted in the best yield (99%) of the expected product **8a**. Screening of the solvents showed that the reaction proceeded effectively in 1,2-DCE, chloroform, and EtOH, among which 1,2-DCE afforded the highest yield of **8a** (Table 1, entries 1, 9, and 10). In contrast, THF, toluene, DMF, and DMSO were inefficient reaction solvents (entries 11–14). Thus, 1,2-DCE was the best solvent for the reaction in terms of the product yield of **8a** (99%), while chloroform posed a concern of acid contamination. Examination of the optimum amount of reagents **2a** and **6a** toward **1a** proved that the reaction of **1a** with **2a** and Ph_3_BiCl_2_ **6a** in the ratio of 1:2:2 provided the best results, affording product **8a** in the highest yield (99%) (Table 1, entries 1, 15, and 16). Moreover, in the presence of 30 mol % of **6a** and a **1a**:**2a** ratio of 1:2, the reaction was suppressed and the bismuth reagent did not catalyze it (entry 17). The addition of triethylamine as the base afforded **8a** in a low yield (32%) (entry 18). At room temperature, the reaction hardly proceeded (entry 19). Screening various thioamides (**2a**–**5**) showed different behavior in the reaction with **1a** and **6a**; **2a** afforded the desired product **8a** in an excellent yield, whereas the *N*,*N*-disubstituted thioamide **5** did not react (Table 1, entries 1, 20–22). These results show that *N*-phenylbenzothioamide (**2a**) is superior as a C1 unit donor at the 2-position of benzoxazole. Consequently, the best result was obtained when **1a** and **2a** were reacted in the presence of **6a** in 1,2-DCE at 60 °C under aerobic conditions. The optimal reagent, Ph_3_BiCl_2_ (**6a**), can be easily and inexpensively synthesized on a 10 g scale by scaling up the reported procedure (see Supporting Information File 1) [36–37].

**Table 1:** Screening of reaction conditions^a^.

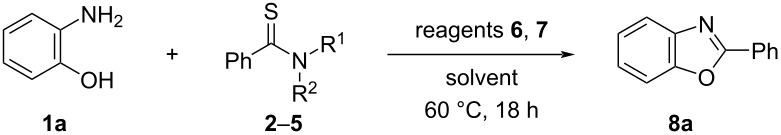						

Entry	Compound	R^1^	R^2^	Reagent	Solvent	Yield [%]^b^

1	**2a**	Ph	H	Ph_3_BiCl_2_ **6a**	1,2-DCE	99 (94)^c^
2	**2a**	Ph	H	Ph_3_Bi(OAc)_2_ **6b**	1,2-DCE	12
3	**2a**	Ph	H	Ph_3_Bi **6c**	1,2-DCE	23
4	**2a**	Ph	H	BiCl_3_ **6d**	1,2-DCE	38
5	**2a**	Ph	H	Ph_3_SbCl_2_ **7a**	1,2-DCE	24
6	**2a**	Ph	H	Ph_3_Sb(OAc)_2_ **7b**	1,2-DCE	60
7	**2a**	Ph	H	Ph_3_Sb **7c**	1,2-DCE	–
8	**2a**	Ph	H	SbCl_3_ **7d**	1,2-DCE	28
9	**2a**	Ph	H	Ph_3_BiCl_2_ **6a**	CHCl_3_	98
10	**2a**	Ph	H	Ph_3_BiCl_2_ **6a**	EtOH	82
11	**2a**	Ph	H	Ph_3_BiCl_2_ **6a**	THF	54
12	**2a**	Ph	H	Ph_3_BiCl_2_ **6a**	toluene	49
13	**2a**	Ph	H	Ph_3_BiCl_2_ **6a**	DMF	22
14	**2a**	Ph	H	Ph_3_BiCl_2_ **6a**	DMSO	22
15^d^	**2a**	Ph	H	Ph_3_BiCl_2_ **6a**	1,2-DCE	55
16^e^	**2a**	Ph	H	Ph_3_BiCl_2_ **6a**	1,2-DCE	50
17^f^	**2a**	Ph	H	Ph_3_BiCl_2_ **6a**	1,2-DCE	27
18^g^	**2a**	Ph	H	Ph_3_BiCl_2_ **6a**	1,2-DCE	32
19^h^	**2a**	Ph	H	Ph_3_BiCl_2_ **6a**	1,2-DCE	32
20	**3**	H	H	Ph_3_BiCl_2_ **6a**	1,2-DCE	22
21	**4**	Me	H	Ph_3_BiCl_2_ **6a**	1,2-DCE	62
22	**5**	Ph	Me	Ph_3_BiCl_2_ **6a**	1,2-DCE	–

^a^Reaction conditions: **1a** (0.5 mmol), **2a**–**5** (1.0 mmol), pnictogen reagent (**6** or **7**: 1.0 mmol); ^b^GC yield using dibenzyl as internal standard; ^c^Isolated yield; ^d^**1a** (0.5 mmol), **2a** (0.75 mmol), **6a** (0.75 mmol); ^e^**1a** (0.5 mmol), **2a** (0.5 mmol), **6a** (0.5 mmol); ^f^**6a** (30 mol %); ^g^Addition of Et_3_N (1.0 mmol); ^h^At room temperature.

To investigate the efficiency and generality of the above-described cyclization, the reaction of various aminophenols **1** (0.5 mmol) and thioamides **2** (1.0 mmol) was investigated in the presence of Ph_3_BiCl_2_ **6a** (1.0 mmol) under the optimized conditions. The results are summarized in Table 2. The reaction of aminophenol (**1a**) with thioamides **2b**–**g** bearing electron-donating or electron-withdrawing groups on the phenyl rings (R^2^) afforded the corresponding 2-arylbenzoxazoles **8b**–**g** in good to excellent yields (79–99%). The nature of substituents on the benzene rings of the thioamides did not significantly affect the reaction outcome. Sterically hindered thioamides bearing ortho-substituted aryl groups readily reacted to furnish the corresponding benzoxazoles **8h**–**j**; further, the reaction with a thioamide bearing a thiophene ring gave the expected product **8k** in excellent yield (96%). Moreover, thioamides bearing alkyl groups (R^2^ = cyclohexyl, methyl) reacted with aminophenol **1a** to afford the 2-alkylbenzoxazoles **8l** and **8m**. Various aminophenols bearing different electron-donating and electron-withdrawing groups at the 4-position of the benzene ring were treated with **2a** and **6a** to afford the corresponding products **8n**–**r** in good to excellent yields. Subsequently, 2-aminophenols with methyl groups at the 3-, 4-, and 5-positions provided satisfactory yields of the products **8o**, **8s**, and **8t**, respectively. On the other hand, an aminophenol with a methyl group at the 6-position provided the product **8u** in a low yield. The reaction of 3-amino-2-naphthol with **2a** furnished the tricyclic compound **8v** in 84% yield. In contrast, the reaction with 3-amino-2-anthracenol resulted in a low yield of the tetracyclic compound **8w** due to the low solubility of aminoanthracenol. The reaction of **2a** with 2-aminothiophenol instead of 2-aminophenol proceeded smoothly, and the corresponding 2-phenylbenzothiazole (**9**) was isolated in 93% yield.

**Table 2:** Synthesis of 2-substituted benzazoles^a^.

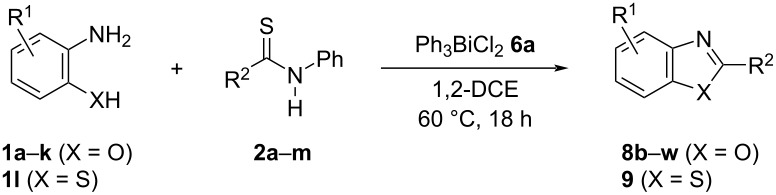			

Product	Yield (%)^b^	Product	Yield (%)^b^

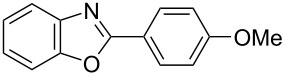 **8b**	95	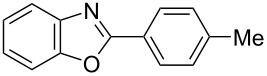 **8c**	97
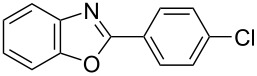 **8d**	99	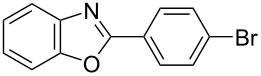 **8e**	92
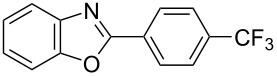 **8f**	79	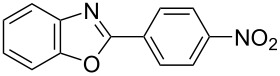 **8g**	99
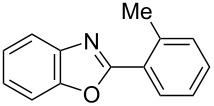 **8h**	89 (5 h)	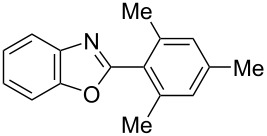 **8i**	78 (24 h)
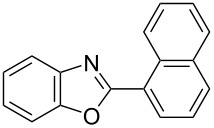 **8j**	91	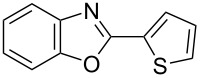 **8k**	96
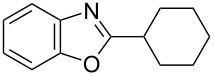 **8l**	97	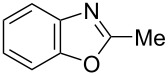 **8m**	80
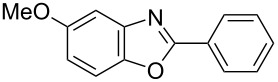 **8n**	99	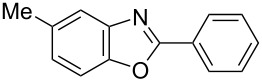 **8o**	84
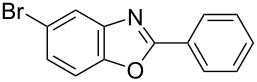 **8p**	94	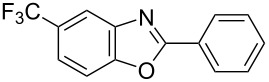 **8q**	99
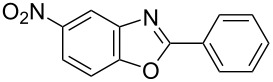 **8r**	99	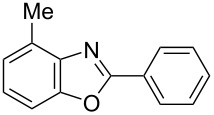 **8s**	95
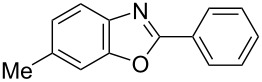 **8t**	94	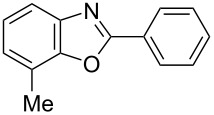 **8u**	55
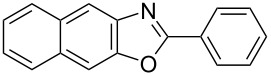 **8v**	84	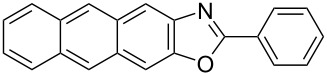 **8w**	48
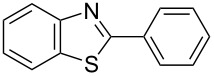 **9**	93		

^a^Reagents and conditions: **1** (0.5 mmol), **2** (1.0 mmol), and **6a** (1.0 mmol) in 1,2-DCE at 60 °C; ^b^Isolated yield.

**Scheme 2:**
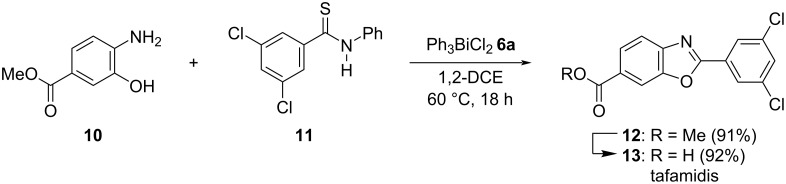
Synthesis of tafamidis (**13**).

Tafamidis (**13**), a compound with a 2-arylbenzoxazole skeleton is a clinically used drug for transthyretin amyloid inhibition [38–39], and was first synthesized by Kelly et al. [40]. We synthesized compound **13** by the developed cyclodesulfurization method (Scheme 2). The reaction of methyl 4-amino-3-hydroxybenzoate (**10**) with 3,5-dichloro-*N*-phenylbenzothioamide (**11**) afforded the benzoxazole **12** in 91% yield. The subsequent hydrolysis of compound **12** then afforded the desired product **13** in 92% yield (84% overall). On the other hand, an attempt at the direct synthesis of compound **13** from 4-amino-3-hydroxybenzoic acid, unfortunately, yielded a complex mixture and product **13** was not obtained.

In the next stage, the synthesis of 2-substituted benzimidazoles was examined using the same protocol (Table 3). However, the reaction of *o*-phenylenediamine (**14**) with **2a** in the presence of **6a** did not yield the desired 2-phenylbenzimidazole **16a** and resulted in a complex mixture. Since the generation of acids such as hydrochloric acid was expected in the reaction system, the reaction was examined using *N*-tosyl-*o*-phenylenediamine (**15**) protected with tosyl group instead of the diamine **14**, and the corresponding product **16c** was obtained. The reaction of **15** with various thioamides **2** bearing electron-donating or electron-withdrawing groups on the phenyl rings, sterically hindered aryl groups, heteroaryl groups, and alkyl groups also proceeded efficiently, and the corresponding products **16b**–**i** were obtained in 70–87% yields, similarly to the syntheses of benzoxazoles.

**Table 3:** Synthesis of 2-substituted *N*-tosylbenzimidazoles^a^.

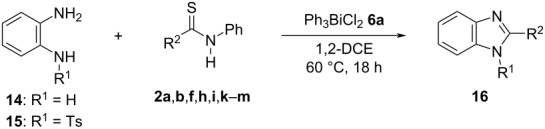			

Product	Yield (%)^b^	Product	Yield (%)^b^

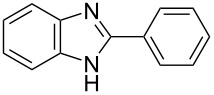 **16a**	0	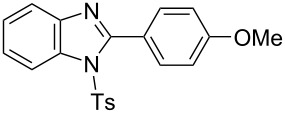 **16b**	76
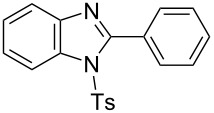 **16c**	85	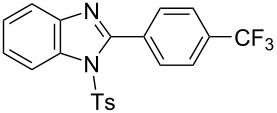 **16d**	79
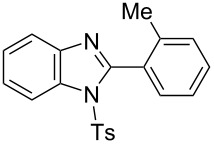 **16e**	87	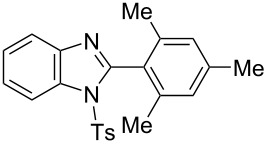 **16f**	75
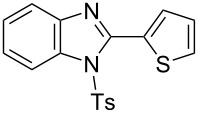 **16g**	70	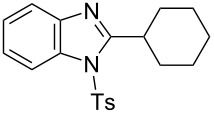 **16h**	72
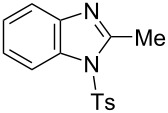 **16i**	73		

^a^Reagents and conditions: **1** (0.5 mmol), **2** (1.0 mmol), and **6a** (1.0 mmol) in 1,2-DCE at 60 °C; ^b^Isolated yield.

**Scheme 3:**
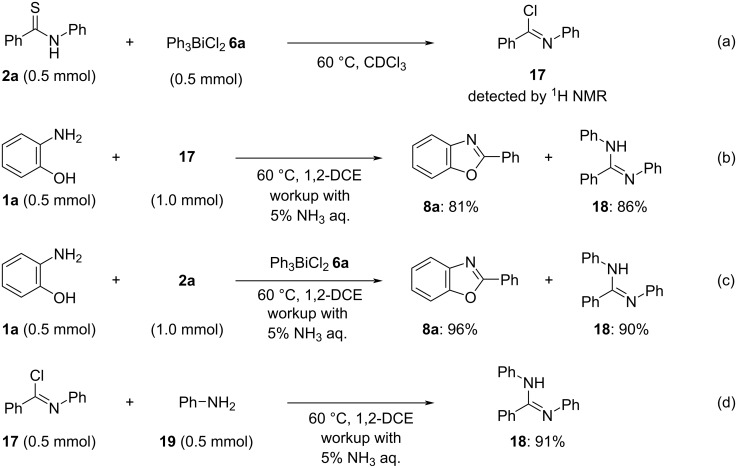
Control experiments.

**Scheme 4:**
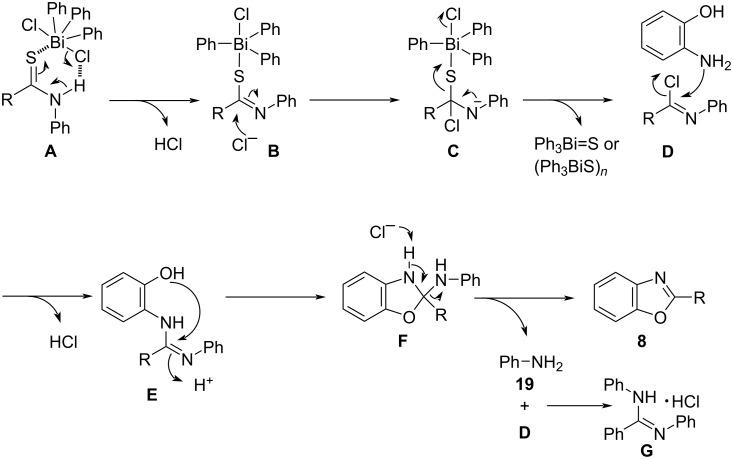
Proposed mechanism.

A control experiment was carried out to investigate the reaction pathway and mechanism. When the reaction of benzothioamide (**2a**) with Ph_3_BiCl_2_ **6a** in chloroform-*d* at 60 °C was monitored by ^1^H NMR spectroscopy, phenylbenzimidoyl chloride (**17**) was observed to be generated (Scheme 3a) (see Supporting Information File 1 for details). When *o*-aminophenol (**1a**) was reacted with **17** [41] in a 1:2 ratio, product **8a** and diphenylbenzamidine (**18**) were obtained in 81% and 86% yields, respectively, after purification by acid–base workup (Scheme 3b). A similar workup was performed for the reaction of **1a** and **2a** in the presence of **6a** under standard conditions, and compounds **8a** and **18** were isolated, respectively, in high yields (Scheme 3c). The reaction of **17** with aniline **19** afforded product **18** in 91% yield (Scheme 3d). These results suggest that the generation of benzamidine **18** by-produces aniline (**19**). On the other hand, aniline generation was not confirmed in the reaction between **1a** and **2a** without an acid–base workup (Table 1, entry 1). Based on the above control experiments and the reaction under study (which required no base; Table 1, entry 18), a possible mechanism for this cyclodesulfurization approach is shown in Scheme 4. The formation of intermediate **A** based on S···Bi [42–43] and Cl···H inter-coordination is anticipated from the reaction of thioamide **2** and Ph_3_BiCl_2_ **6a** as an initial step. With the elimination of hydrochloric acid, intermediate **A** is converted to intermediate **B**. When a base such as Et_3_N was added, hydrochloric acid was trapped and lowered the reaction yield (Table 1, entry 18). The nucleophilic attack of chloride ions on intermediate **B** produces **D** via **C**, which entails isomerization with the elimination of the sulfur-and-bismuth moiety. Aminophenol then reacts with **D** to generate intermediate **F** via **E**, which is converted to the benzoxazole **8**, accompanied by the elimination of **19** by aromatization. The generation of hydrochloric acid was important in this reaction, and the addition of Et_3_N resulted in lower yield because the hydrochloric acid was trapped by the base (Table 1, entry 18). This reaction required two equivalents of thioamide **2** and Ph_3_BiCl_2_ **6a** for aminophenol (Table 1, entries 1, 15–17). The produced aniline **19** reacts with an excessive amount of **D** to form benzamidine hydrochloride **G**. The reaction of **D** with amines may require an excessive amount of **D** due to competition between aminophenol and the byproduct aniline. A similar mechanism is considered for the construction of benzimidazole and thiazole rings. On the other hand, the released bismuth moiety, Ph_3_Bi=S or (Ph_3_BiS)*_n_*, that was expected to be produced in this process, was not confirmed or isolated at this point.

## Conclusion

We developed a novel method for the Ph_3_BiCl_2_-promoted cyclodesulfurization reaction between thioamides and 2-aminophenols, *N*-tosyl-1,2-phenylenediamines, and 2-aminobenzenethiol, and achieved the syntheses of 2-aryl- and 2-alkylbenzazoles, such as oxazoles, imidazoles, and thiazole, in satisfactory yields. The protocol was successfully applied to the synthesis of tafamidis, a clinically used drug for transthyretin amyloid inhibition. The reaction is simple, can be performed in aerobic conditions, and requires no bases. In this system, Ph_3_BiCl_2_ acts as a superior reagent for the desulfurization and chlorination of thioamides into benzimidoyl chloride as a reaction intermediate. On the other hand, the reaction still has the disadvantage of requiring excess amounts of Ph_3_BiCl_2_ and thioamide. Further investigations to expand the cyclodesulfurization reaction to other substrates and the development of a catalytic reaction using bismuth reagent are in progress, and the results will be reported in due course.

## Experimental

### General procedure for the synthesis of 2-arylazoles

A mixture of aminophenol derivatives (**1**, **10** or **15**; 0.5 mmol), *N*-phenylthiobenzamide (**2** or **11**; 0.5 mmol), and Ph_3_BiCl_2_ (**6a**; 1.0 mmol) were well stirred at 60 °C in 1,2-DCE (3.0 mL) for 18 h. After completion of the reaction, the reaction mixture was diluted with H_2_O (20 mL) and CH_2_Cl_2_ (20 mL), and the aqueous phase was extracted with CH_2_Cl_2_ (3 × 30 mL). The combined organic phase was washed with brine (20 mL) and dried over MgSO_4_. Evaporation of the solvent furnished the crude product which was then purified by column chromatography on silica gel.

## Supporting Information

File 1 Experimental procedures, characterization data and copies of spectra.
