# Supplementary material for: One-pot synthesis of 2-arylated and 2-alkylated benzoxazoles and benzimidazoles based on triphenylbismuth dichloride-promoted desulfurization of thioamides
Source: Beilstein J Org Chem. 2022 Oct 18;18:1479–87. doi: 10.3762/bjoc.18.155 (PMC9592962; doi:10.3762/bjoc.18.155)
Supplement: File 1 — Experimental procedures, characterization data and copies of spectra. [file Beilstein_J_Org_Chem-18-1479-s001.pdf]

BEILSTEIN JOURNAL OF ORGANIC CHEMISTRY

Supporting Information

for

One-pot synthesis of 2-arylated and 2-alkylated benzoxazoles and benzimidazoles based on triphenylbismuth dichloride-promoted desulfurization of thioamides

Arisu Koyanagi, Yuki Murata, Shiori Hayakawa, Mio Matsumura and Shuji Yasuike

Beilstein J. Org. Chem. **2022,** *18,* 1479–1487. doi:10.3762/bjoc.18.155

Experimental procedures, characterization data and copies of spectra

License and Terms: This is a supporting information file under the terms of the Creative Commons Attribution License (https://creativecommons.org/licenses/by/4.0). Please note that the reuse, redistribution and reproduction in particular requires that the author(s) and source are credited and that individual graphics may be subject to special legal provisions.
The license is subject to the *Beilstein Journal of Organic Chemistry* terms and conditions: (https://www.beilstein-journals.org/bjoc/terms)

Table of contents

1. General information S1
2. Synthesis of triphenylbismuth dichloride (Ph_3BiCl_2) S1
3. Synthesis of 2-substituted benzoxazoles S2
4. Synthesis of 2-substituted benzimidazoles S9
5. References S12
6. Copies of ^{1}H, ^{13}C, and ^{19}F NMR spectra S13
7. NMR Experiments S50

1. General information

Unless otherwise stated, all reagents and solvents were purchased from commercial suppliers and were used without further purification. ^{1}H NMR (TMS: δ 0.00 ppm, $CHCl_3$: δ 7.26 ppm or DMSO: δ 2.50 ppm as an internal standard), ^{13}C NMR ($CDCl_3$: δ 77.0 ppm or DMSO-d_6: δ 39.5 ppm as an internal standard) and ^{19}F NMR (trifluoromethylbenzene: δ −64.0 ppm as an internal standard) spectra were recorded on a JEOL ECZ-400S (400 MHz, 100 MHz and 376 MHz) spectrometer in $CDCl_3$ and DMSO-d_6. Melting points were measured on a Yanagimoto micro melting point hot stage apparatus and are uncorrected. GC–MS (EI) spectra were recorded on an Agilent 5977E Diff-SST MSD-230V spectrometer. HRMS (ESI) were measured on an Agilent 6230 TOF mass spectrometer. IR spectra were recorded on an FTIR-8400S system from Shimadzu spectrometer and are reported in frequency of absorption (cm^{-1}). Only selected IR absorbencies are reported. All chromatographic separations were accomplished with Silica Gel 60N (Kanto Chemical Co., Inc.). Thin-layer chromatography (TLC) was performed with Macherey-Nagel Sil G25 UV254 pre-coated TLC plates. Aminophenols **1a**–**k**, *N*-phenylthiobenzamide (**2a**) and $BiCl_3$ were purchased from TCI Fine Chemicals, Japan. Thiobenzamides **2b**–**m**[1] and *N*-tosyl-*o*-phenylenediamine (**15**)[2] were prepared according to the reported procedures.

2. Synthesis of triphenylbismuth dichloride (Ph_3BiCl_2).

To a solution of prepared triphenylbismuth[3] (10.0 g, 22.7 mmol) in dry CH_2Cl_2 (60 mL) was added SO_2Cl_2 (2.02 mL, 3.37 g, 25.0 mmol) at 0 °C. The reaction mixture was stirred at room temperature for 1 h until complete consumption of the starting material, as monitored by TLC. The resulting mixture was evaporated under reduced pressure and remaining whitish solids were purified by recrystallization from CH_2Cl_2/hexane (9.98 g, 86%, m.p. 151-153 °C). The product was confirmed by comparison of the melting point (lit. m.p. 149-150 °C) and NMR data with that in the literature [4].

3. Synthesis of 2-substituted benzoxazoles.

A mixture of aminophenol **1** (0.5 mmol), *N*-phenylthiobenzamide **2** (1.0 mmol), and Ph_3BiCl_2 (**6a**: 1.0 mmol) were well stirred at 60 °C in 1,2-DCE (3.0 mL) for 18 h. After completion of the reaction, the reaction mixture was diluted with H_2O (20 mL) and CH_2Cl_2 (20 mL), and the aqueous phase was extracted with CH_2Cl_2 (3 × 30 mL). The combined organic phase was washed with brine (20 mL) and dried over $MgSO_4$. Evaporation of the solvent furnished the crude product. The crude product was then purified by column chromatography on silica gel.

2-Phenylbenzoxazole (8a)[5]

91.7 mg (94%). Colorless needles. m.p. 102-103 °C (from Hexane); R_f = 0.5 (Hexane/EtOAc 9:1). ^{1}H NMR (400 MHz, $CDCl_3$): δ = 8.28-8.24 (m, 2H; Ar-H), 7.79-7.75 (m, 1H; Ar-H), 7.59-7.56 (m, 1H; Ar-H), 7.53-7.48 (m, 3H; Ar-H), 7.37-7.33 (m, 2H; Ar-H) ppm; ^{13}C NMR (100 MHz, $CDCl_3$): δ = 163.2 (C), 150.9 (C), 142.2 (C), 131.7 (CH), 129.0 (CH), 127.7 (CH), 127.3 (C), 125.2 (CH), 124.7 (CH), 120.1 (CH), 110.7 (CH) ppm. MS (EI): *m/z* (%): 195 (100, M^+), 167 (17), 63 (14); HRMS: *m/z* [M^+] calcd for $C_{13}H_9NO$: 195.0684. Found: 195.0681.

2-(4-Methoxyphenyl)benzoxazole (8b)[5]

107 mg (95%). Colorless prisms. m.p. 95-97 °C (from Hexane), R_f = 0.3 (Hexane/EtOAc 9 : 1); ^{1}H NMR (400 MHz, $CDCl_3$): δ = 8.19 (dt, *J* = 8.8, 2.8 Hz, 2H; Ar-H), 7.75-7.73 (m, 1H; Ar-H), 7.57-7.54 (m, 1H; Ar-H), 7.35-7.30 (m, 2H, Ar-H), 7.02 (dt, *J* = 9.2, 2.8 Hz, 2H; Ar-H), 3.89 (s, 3H; OMe) ppm; ^{13}C NMR (100 MHz, $CDCl_3$): δ = 163.3 (C), 162.5 (C), 150.7 (C) 142.2 (C), 129.6 (CH), 124.8 (CH), 124.6 (CH), 119.7 (CH), 114.5 (CH), 110.5 (CH), 55.6 (CH_3) ppm. MS (EI): *m/z* (%): 225 (100, M^+), 210 (31), 182 (37), 127 (6); HRMS: *m/z* [M^+] calcd for $C_{14}H_{11}NO_2$: 225.0790. Found: 225.0786.

2-(*p*-Tolyl)benzoxazole (8c)[6]

101 mg (97%). Colorless prisms. m.p. 111-112 °C (from Hexane), R_f = 0.4 (Hexane/EtOAc 9 : 1); ^{1}H NMR (400 MHz, $CDCl_3$): δ = 8.14 (d, *J* = 8.4 Hz, 2H; Ar-H), 7.78-7.74 (m, 1H; Ar-H), 7.59-7.55 (m, 1H; Ar-H), 7.36-7.25 (m, 4H; Ar-H), 2.44 (s, 3H; Me) ppm; ^{13}C NMR (100 MHz, $CDCl_3$): δ = 163.4 (C), 150.8 (C), 142.3 (C), 142.2 (C), 129.8 (CH), 127.7 (CH), 125.0 (CH), 124.6 (CH), 124.5 (C), 120.0 (CH), 110.6 (CH), 21.8 (CH_3) ppm. MS (EI): *m/z* (%): 209 (100, M^+), 180 (8), 91 (4). HRMS: *m/z* [M^+] calcd for $C_{14}H_{11}NO$: 209.0841. Found: 209.0838.

2-(4-Chlorophenyl)benzoxazole (8d)[5]

113 mg (99%). Pale yellow prisms. m.p. 143-145 °C (from Hexane), R_f = 0.3 (Hexane/EtOAc 9:1); ^{1}H NMR (400 MHz, $CDCl_3$): δ = 8.19 (dt, J = 8.8, 2.8 Hz, 2H; Ar-H), 7.78-7.74 (m, 1H; Ar- H), 7.60-7.56 (m, 1H; Ar-H), 7.50 (dt, J = 8.8, 2.4 Hz, 2H; Ar-H), 7.36 (dt, J = 9.2, 4.4 Hz, 2H; Ar-H) ppm; ^{13}C NMR (100 MHz, $CDCl_3$): δ = 162.2 (C), 150.8 (C), 142.0 (C), 137.9 (C), 129.4 (CH), 129.0 (CH), 125.7 (C), 125.5 (CH), 124.9 (CH), 120.2 (CH), 110.8 (CH) ppm. MS (EI): *m/z* (%): 229 (100, M^+), 201 (11), 166 (4). HRMS: *m/z* [M^+] calcd for $C_{13}H_8ClNO$: 229.0294. Found: 229.0297.

2-(4-Bromophenyl)benzoxazole (8e)[5]

126 mg (92%). Yellow needles. m.p. 158-161 °C (from Hexane), R_f = 0.5 (Hexane/EtOAc 9:1); ^{1}H NMR (400 MHz, $CDCl_3$): δ = 8.11 (dt, J = 8.8, 1.6 Hz, 2H; Ar-H), 7.78-7.74 (m, 1H; Ar-H), 7.65 (dt, J = 8.4, 2.0 Hz, 2H; Ar-H), 7.59-7.55 (m, 1H; Ar-H), 7.38-7.33 (m, 2H; Ar-H) ppm; ^{13}C NMR (100 MHz, $CDCl_3$): δ = 162.3 (C), 150.9 (C), 142.1 (C), 132.4 (CH), 129.1 (CH), 126.4 (C), 126.2(C), 125.5 (CH), 124.9 (CH), 120.2 (CH), 110.8 (CH) ppm. MS (EI): *m/z* (%): 273 (100, M^+), 245 (9), 194 (12), 166 (9). HRMS: *m/z* [M^+] calcd for $C_{13}H_8BrNO$: 272.9789. Found: 272.9785.

2-[4-(Trifluoromethyl)phenyl]benzoxazole (8f)[6]

104 mg (79%). Colorless needles. m.p. 135-138 °C (from Hexane), R_f = 0.4 (Hexane/EtOAc 9:1). ^{1}H NMR (400 MHz, $CDCl_3$): δ = 8.38 (d, J = 7.6 Hz, 2H; Ar-H), 7.82-7.78 (m, 3H; Ar-H), 7.64-7.61 (m, 1H; Ar-H), 7.42-7.36 (m, 2H; Ar-H) ppm; ^{13}C NMR (100 MHz, $CDCl_3$): δ = 161.6 (C), 151.0 (C), 142.0 (C), 133.1 (q, $^2J_{C,F}$ = 32.6 Hz, C), 130.6 (C), 128.0 (CH), 126.0 (q, $^3J_{C,F}$ = 3.8 Hz, CH), 125.2 (CH), 125.1 (CH), 123.8 (q, $^1J_{C,F}$ = 271 Hz, C), 120.5 (CH), 110.9 (CH) ppm; ^{19}F NMR (376 MHz, $CDCl_3$): δ = -62.9 (s) ppm. MS (EI): *m/z* (%): 263 (100, M^+), 235 (13), 63 (19). HRMS: *m/z* [M^+] calcd for $C_{14}H_8F_3NO$: 263.0558. Found: 263.0557.

2-(4-Nitrophenyl)benzoxazole (8g)[7]

124 mg (99%). Orange prisms. m.p. 255-258 °C (from CH_2Cl_2-EtOH), R_f = 0.8 (Hexane/EtOAc 9:1); ^{1}H NMR (400 MHz, $CDCl_3$): δ = 8.43 (dt, J = 9.2, 2.0 Hz, 2H; Ar-H), 8.38 (dt, J = 9.2, 2.0 Hz, 2H; Ar-H), 7.84-7.82 (m, 1H; Ar-H), 7.65-7.62 (m, 1H; Ar-H), 7.46-7.39 (m, 2H; Ar-H) ppm; ^{13}C NMR (100 MHz, $CDCl_3$): δ = 160.8 (C), 151.1 (C), 149.5 (C), 142.0 (C), 132.9 (C), 128.5 (CH), 126.5 (C), 125.4 (C), 124.4 (CH), 120.8 (CH), 111.1 (CH) ppm. IR (KBr): $\tilde{\nu}$ = 3093, 2360, 1521, 1450, 1348, 1338, 1057, 854 cm^{-1}. MS (EI): *m/z* (%): 240 (100, M^+), 210 (45), 194 (27), 116 (8). HRMS: *m/z* [M^+] calcd for $C_{13}H_8N_2O_3$: 240.0535. Found: 240.0539.

2-(*o*-Tolyl)benzoxazole (8h)[5]

93.1 mg (89%). Colorless needles. m.p. : 65-67 °C (from Hexane), R_f = 0.5 (Hexane/EtOAc 9:1); ^{1}H (400 MHz, $CDCl_3$): δ = 8.18-8.16 (m, 1H; Ar-H), 7.81-7.79 (m, 1H; Ar-H), 7.60-7.58 (m, 1H; Ar-H), 7.43-7.40 (m, 1H; Ar-H), 7.38-7.32 (m, 4H; Ar-H), 2.81 (s, 3H; Me); ^{13}C NMR (100 MHz, $CDCl_3$): δ = 163.5 (C), 150.4 (C), 142.2 (C), 139.0 (C), 131.9 (CH), 131.0 (CH), 130.1 (CH), 126.4 (C), 126.2 (CH), 125.1 (CH), 124.5 (CH), 120.3 (CH), 110.6 (CH), 22.4 (CH_3) ppm. MS (EI): *m/z* (%): 209 (100, M^+), 180 (40), 152 (8), 116 (5). HRMS: *m/z* [M^+] calcd for $C_{14}H_{11}NO$: 209.0841. Found: 209.0837.

2-Mesitylbenzoxazole (8i)[8]

92.5 mg (78%). Pale yellow oil. R_f = 0.5 (Hexane/EtOAc 9:1); ^{1}H NMR (400 MHz, $CDCl_3$): δ = 7.84-7.80 (m, 1H; Ar-H), 7.60-7.56 (m, 1H; Ar-H), 7.40-7.56 (m, 2H; Ar-H), 6.97 (s, 2H; Ar-H), 2.35 (s, 3H; Me), 2.29 (s, 6H; Me) ppm; ^{13}C NMR (100 MHz, $CDCl_3$): δ = 163.4 (C), 150.7 (C), 141.7 (C), 140.4 (C), 138.6 (C), 128.8 (CH), 125.1 (C), 125.0 (CH), 124.3 (CH), 120.3 (CH), 110.7 (CH), 21.5 (CH_3), 20.5 (CH_3) ppm. MS (EI): *m/z* (%): 237 (100, M^+), 222 (22), 208 (21), 194 (7). HRMS: *m/z* [M^+] calcd for $C_{16}H_{15}NO$: 237.1154. Found: 237.1156.

2-(Naphthalen-1-yl)benzoxazole (8j)[9]

111 mg (91%). Yellow plates. m.p. 104-107 °C (from Hexane), R_f = 0.6 (Hexane/EtOAc 9:1); ^{1}H NMR (400 MHz, $CDCl_3$): δ = 9.46 (d, *J* = 8.8 Hz, 1H; Ar-H), 8.43 (dd, *J* = 6.0, 1.0 Hz,1H; Ar-H), 8.03 (d, *J* = 8.0 Hz, 1H; Ar-H), 7.94 (d, *J* = 8.0 Hz, 1H; Ar-H), 7.90-7.88 (m, 1H; Ar-H), 7.71 (ddd, *J* = 8.4, 6.8, 1.6 Hz, 1H; Ar-H), 7.67-7.59 (m, 3H; Ar-H), 7.42-7.39 (m, 2H; Ar-H) ppm; ^{13}C NMR (100 MHz, $CDCl_3$): δ = 162.9 (C), 150.3 (C), 142.4 (C), 134.1 (C), 132.5 (CH), 130.8 (C), 129.5 (CH), 128.8 (CH), 128.1 (CH), 126.6 (CH), 126.4 (CH), 125.4 (CH), 125.1 (CH), 124.6 (CH), 123.7 (C), 120.4 (CH), 110.7 (CH) ppm. MS (EI): *m/z* (%): 245 (100, M^+), 216 (9), 153 (8). HRMS: *m/z* [M^+] calcd for $C_{17}H_{11}NO$: 245.0841. Found: 245.0839.

2-(Thiophen-2-yl)benzoxazole (8k)[5]

96.7 mg (96%). Pale yellow prisms. m.p. 99-102 °C (from Hexane), R_f = 0.5 (Hexane/EtOAc 9:1); ^{1}H NMR (400 MHz, $CDCl_3$): δ = 7.91 (dd, *J* = 3.6, 0.8 Hz, 1H; Ar-H), 7.75-7.71 (m, 1H; Ar-H), 7.56-7.53 (m, 2H; Ar-H), 7.36-7.31 (m, 2H; Ar-H), 7.18 (dd, *J* = 3.6, 0.8 Hz, 1H; Ar-H) ppm; ^{13}C NMR (100 MHz, $CDCl_3$): δ = 159.2 (C), 150.5 (C), 142.1 (C), 130.4 (CH), 130.1 (CH), 129.7 (C), 128.4 (CH), 125.2 (CH), 124.9 (CH), 119.9 (CH), 110.6 (CH) ppm. MS (EI): *m/z* (%): 201 (100, M^+), 173 (9), 92 (3). HRMS: *m/z* [M^+] calcd for $C_{11}H_7NOS$: 201.0248. Found: 201.0244.

2-Cyclohexylbenzoxazole (8l)[10]

97.7 mg (97%). Yellow oil. R_f = 0.5 (Hexane/EtOAc 9:1); ^{1}H NMR (400 MHz, $CDCl_3$): δ = 7.68-7.66 (m, 1H; Ar-H), 7.47-7.45 (m, 1H; Ar-H), 7.30-7.26 (m, 2H; Ar-H), 2.98-2.92 (m, 1H; Cy), 2.18-2.14 (m, 2H; Cy), 1.89-1.83 (m, 2H; Cy), 1.75-1.64 (m, 3H; Cy), 1.47-1.25 (m, 3H; Cy) ppm; ^{13}C NMR (100 MHz, $CDCl_3$): δ = 170.6 (C), 150.6 (C), 141.1 (C), 124.5 (CH), 124.2 (CH), 119.7 (CH), 110.4 (CH), 38.0 (CH), 30.6 (CH_2), 25.9 (CH_2), 25.8 (CH_2) ppm. MS (EI): *m/z* (%): 201 (12, M^+), 172 (18), 146(100), 120 (6). HRMS: *m/z* [M^+] calcd for $C_{13}H_{15}NO$: 201.1154. Found: 201.1151.

2-Methylbenzoxazole (8m)[11]

53.3 mg (80%). Colorless oil, R_f = 0.5 (Hexane/EtOAc 9:1). ^{1}H NMR (400 MHz, $CDCl_3$): δ = 7.66-7.62 (m, 1H; Ar-H), 7.47-7.43 (m, 1H; Ar-H), 7.28-7.25 (m, 2H; Ar-H), 2.63 (s, 3H; Me) ppm; ^{13}C NMR (100 MHz, $CDCl_3$): δ = 163.9 (C), 151.1 (C), 141.6 (C), 124.6 (CH), 124.2 (CH), 119.5 (CH), 110.3 (CH), 14.7 (CH_3) ppm. MS (EI): *m/z* (%): 133 (100, M^+), 104 (20), 63 (21). HRMS: *m/z* [M^+] calcd for C_8H_7NO: 133.0528. Found: 133.0530.

5-Methoxy-2-phenylbenzoxazole (8n)[5]

113 mg (99%). Colorless needles. m.p. 65-68 °C (from Hexane), R_f = 0.4 (Hexane/EtOAc 9:1); ^{1}H NMR (400 MHz, $CDCl_3$): δ = 8.24-8.21 (m, 2H; Ar-H), 7.52-7.44 (m, 3H; Ar-H), 7.45 (*d*, *J* = 8.7 Hz, 1H; Ar-H), 7.28-7.25 (m, 1H; Ar-H), 6.94 (dd, *J* = 9.1, 2.7 Hz, 1H; Ar-H), 3.87 (s, 3H; Me) ppm; ^{13}C NMR (100 MHz, $CDCl_3$): δ = 163.9 (C), 157.5 (C), 145.5 (C), 142.9 (C), 131.6 (CH), 129.1 (CH), 127,6 (CH), 127.3 (C), 113.9 (CH), 110.9 (CH),102.9 (CH), 56.1 (CH_3) ppm. MS (EI): *m/z* (%): 225 (100, M^+), 210 (71), 107 (22). HRMS: *m/z* [M^+] calcd for $C_{14}H_{11}NO_2$: 225.0790. Found: 225.0788.

5-Methyl-2-phenylbenzoxazole (8o)[6]

87.5 mg (84%). Colorless plates. m.p. 96-98 °C (from Hexane), R_f = 0.4 (Hexane/EtOAc 9:1); ^{1}H NMR (400 MHz, $CDCl_3$): δ = 8.25-8.23 (m, 2H; Ar-H), 7.53-7.50 (m, 3H; Ar-H), 7.49-7.44 (m, 1H; Ar-H), 7.45 (d, *J* = 8.7 Hz; 1H, Ar-H), 7.16 (dd, *J* = 8.2, 0.9 Hz, 1H; Ar-H), 2.48 (s, 3H; Me) ppm. ^{13}C NMR (100 MHz, $CDCl_3$): δ = 163.2 (C), 149.0 (C), 142.2 (C), 134.6 (C), 131.6 (CH), 129.0 (CH), 127.7 (CH), 127.3 (C), 126.4 (CH), 120.0 (CH), 110.1 (CH), 21.7 (CH_3) ppm. MS (EI): *m/z* (%): 209 (100, M^+), 180 (9), 105 (5). HRMS: *m/z* [M^+] calcd for $C_{14}H_{11}NO$: 209.0841. Found: 209.0845.

5-Bromo-2-phenylbenzoxazole (8p)[6]

129 mg (94%). Red prisms. m.p. 106-108 °C (from Hexane), R_f = 0.4 (Hexane/EtOAc 9:1); ^{1}H NMR (400 MHz, $CDCl_3$): δ = 8.25-8.23 (m, 2H; Ar-H), 7.90 (s, 1H; Ar-H), 7.58-7.50 (m, 3H; Ar-H), 7.46 (s, 2H; Ar-H) ppm. ^{13}C NMR (100 MHz, $CDCl_3$): δ = 164.3 (C), 149.9 (C), 143.8 (C), 132.1 (CH), 129.1 (CH), 128.2 (CH), 127.9 (CH), 126.7 (C), 123.1 (CH), 117.5 (C), 112.0 (CH) ppm. MS (EI): *m/z* (%): 273 (100, M^+), 245 (11), 143 (6). HRMS: *m/z* [M^+] calcd for $C_{13}H_8BrNO$: 272.9789. Found: 272.9792.

2-Phenyl-5-(trifluoromethyl)benzoxazole (8q)[6]

130 mg (99%). Colorless plates. m.p. 75-77 °C (from Hexane), R_f = 0.5 (Hexane/EtOAc 9:1); ^{1}H NMR (400 MHz, $CDCl_3$): δ = 8.26 (dd, *J* = 9.6, 1.8 Hz, 2H; Ar-H), 8.04 (s, 1H; Ar-H), 7.68 (d, *J* = 8.2 Hz, 1H; Ar-H), 7.61 (dd, *J* = 6.4, 1.6 Hz, 1H; Ar-H), 7.58-7.52 (m, 3H; Ar-H) ppm; ^{13}C NMR (100 MHz, $CDCl_3$): δ = 164.9 (C), 152.6 (C), 142.3 (C), 132.3 (CH), 129.2 (CH), 128.0 (CH), 127.4 (q, $^1J_{C,F}$ = 271 Hz, C), 126.6 (C), 124.3 (q, $^2J_{C,F}$ = 36.4 Hz; C), 122.4 (q, $^3J_{C,F}$ = 2.9 Hz; CH), 117.8 (q, $^3J_{C,F}$ = 3.8 Hz; CH), 111.2 (CH) ppm; ^{19}F NMR (376 MHz, $CDCl_3$): δ = -61.0 (s) ppm. MS (EI): *m/z* (%): 263 (100, M^+), 235 (16), 132 (7). HRMS: *m/z* [M^+] calcd for $C_{14}H_8F_3NO$: 263.0558. Found: 263.0555.

5-Nitro-2-phenylbenzoxazole (8r)[12]

132 mg (99%). Colorless needles. m.p. 167-169 °C (from Hexane), R_f = 0.4 (Hexane/EtOAc 8:2). ^{1}H NMR (400 MHz, $CDCl_3$): δ = 8.64 (d, *J* = 2.3 Hz, 1H; Ar-H), 8.32 (dd, *J* = 8.7, 2.3 Hz, 1H; Ar-H), 8.27-8.25 (m, 2H; Ar-H), 7.68 (d, *J* = 9.2 Hz, 1H; Ar-H), 7.63-7.53 (m, 3H; Ar-H) ppm; ^{13}C NMR (100 MHz, $CDCl_3$): δ = 166.1 (C), 154.4 (C), 145.5 (C), 142.7 (C), 132.8 (CH), 129.3 (CH), 128.2 (CH), 126.1 (C), 121.3 (CH), 116.4 (CH), 110.9 (CH) ppm. IR (KBr): $\tilde{\nu}$ = 3099, 2341, 1616, 1527, 1448, 1350, 1338, 1265, 1238, 1066, 1022, 819 cm^{-1}. MS (EI): *m/z* (%): 240 (100, M^+), 210 (19), 194 (38), 91 (34). HRMS: *m/z* [M^+] calcd for $C_{13}H_8N_2O_3$: 240.0535. Found: 240.0539.

4-Methyl-2-phenylbenzoxazole (8s)[5]

99.3 mg (95%). Reddish needles. m.p. 83-85 °C (from Hexane), R_f = 0.4 (Hexane/EtOAc 9:1). ^{1}H NMR (400 MHz, $CDCl_3$): δ = 8.28-8.25 (m, 2H; Ar-H), 7.53-7.50 (m, 3H; Ar-H), 7.40 (d, *J* = 8.2 Hz, 1H; Ar-H), 7.23 (t, *J* = 8.0 Hz, 1H; Ar-H), 7.14 (dt, *J* = 7.3, 0.9 Hz, 1H; Ar-H), 2.68 (s, 3H; Me) ppm; ^{13}C NMR (100 MHz, $CDCl_3$): δ = 162.4 (C), 150.6 (C), 141.5 (C), 131.4 (CH), 130.7 (CH), 129.0 (CH), 127.7 (CH), 127.5 (C), 125.2 (CH), 124.9 (CH), 108.0 (CH), 16.8 (CH_3) ppm. MS (EI): *m/z* (%): 209 (100, M^+), 180 (18), 105 (11), 78 (31). HRMS: *m/z* [M^+] calcd for $C_{14}H_{11}NO$:209.0841. Found: 209.0842.

6-Methyl-2-phenylbenzoxazole (8t)[5]

98.1 mg (94%). Colorless plates. m.p. 83-84 °C (from Hexane), R_f = 0.4 (Hexane/EtOAc 9:1). ^{1}H NMR (400 MHz, $CDCl_3$): δ = 8.25-8.20 (m, 2H; Ar-H), 7.63 (d, J = 8.2 Hz, 1H; Ar-H), 7.53-7.45 (m, 3H; Ar-H), 7.38 (s, 1H; Ar-H), 7.16 (dd, J = 7.8, 0.9 Hz, 1H; Ar-H), 2.50 (s, 3H; Me) ppm; ^{13}C NMR (100 MHz, $CDCl_3$): δ = 162.7 (C), 151.1 (C), 140.0 (C), 135.7 (C), 131.5 (CH), 129.0 (CH), 127.6 (CH), 127.4 (C), 126.0 (CH), 119.4 (CH), 110.9 (CH), 22.0 (CH_3) ppm. MS (EI): m/z (%): 209 (100, M^+), 180 (15), 105 (12), 78 (32). HRMS: m/z [M^+] calcd for $C_{14}H_{11}NO$: 209.0841. Found: 209.0837.

7-Methyl-2-phenyl-benzoxazole (8u)[13]

57.8 mg (55%). Brown oil. R_f = 0.4 (Hexane/EtOAc 9:1). ^{1}H NMR (400 MHz, $CDCl_3$): δ = 8.29-8.24 (m, 2H; Ar-H), 7.59 (d, J = 7.8 Hz, 1H; Ar-H), 7.54-7.51 (m, 3H; Ar-H), 7.24 (t, J = 7.3 Hz, 1H; Ar-H), 7.14 (d, J = 7.8 Hz, 1H; Ar-H), 2,60 (s, 3H; CH_3) ppm; ^{13}C NMR (100 MHz, $CDCl_3$): δ = 162. 8 (C), 150.1 (C), 141.8 (C), 131.5 (CH), 129.0 (CH), 127.7 (CH), 127.5 (C), 126.2 (CH), 124.6 (CH), 121.3 (C), 117.4 (CH), 15.4 (CH_3) ppm. MS (EI): m/z (%): 209 (100, M^+), 106 (27), 78 (27). HRMS: m/z [M^+] calcd for $C_{13}H_8ClNO$: 209.0841. Found: 209.0840.

2-Phenylnaphtho[2,3-*d*]oxazole (8v)[5]

103 mg (84%). Pale yellow needles. m.p. 202-204 °C (from Hexane), R_f = 0.3 (Hexane/EtOAc 9:1). ^{1}H NMR (400 MHz, $CDCl_3$): δ = 8.35-8.33 (m, 2H; Ar-H), 8.20 (s, 1H; Ar-H), 8.02-7.95 (m, 3H; Ar-H), 7.61-7.53 (m, 3H; Ar-H), 7.52-7.47 (m, 2H; Ar-H) ppm. ^{13}C NMR (100 MHz, $CDCl_3$): δ = 165.1 (C), 149.8 (C), 142.0 (C), 132.3 (CH), 131.9 (C), 131.7 (C), 129.1 (CH), 128.7 (CH), 128.3 (CH), 128.0 (CH), 127.0 (C), 125.6 (CH), 124.9 (CH), 117.4 (CH), 106.5 (CH) ppm. MS (EI): m/z (%): 245 (100, M^+), 217 (13), 140 (9), 114 (30). HRMS: m/z [M^+] calcd for $C_{17}H_{11}NO$: 245.0841. Found: 245.0845.

2-Phenylanthra[2,3-*d*]oxazole (8w)

71.0 mg (48%). Yellow needles. mp : 254-257 °C (from Toluene), R_f = 0.5 (Hexane/EtOAc 8:2). ^{1}H NMR (400 MHz, $CDCl_3$): δ = 8.62 (s, 1H; Ar-H), 8.55 (s, 1H; Ar-H), 8.38-8.35 (m, 3H; Ar-H), 8.08 (s, 1H; Ar-H), 8.02-8.00 (m, 2H; Ar-H), 7.62-7.55 (m, 3H; Ar-H), 7.48-7.42 (m, 2H; Ar-H) ppm; ^{13}C NMR (100 MHz, $CDCl_3$): δ = 165.8 (C), 149.9 (C), 132.5 (CH), 131.5 (C), 130.9 (C), 130.38 (C), 130.36 (C), 129.1×2 (CH, C), 128.5 (CH), 128.1 (CH), 127.8 (CH), 127.0 (CH), 126.9 (C), 126.1 (CH), 125.5 (CH), 125.2 (CH), 117.0 (CH), 105.4 (CH) ppm. IR (KBr): $\tilde{v}$ = 3047, 2359, 1616, 1546, 1485, 1448, 1261, 1188, 1047, 1020, 895 cm^{-1}. MS (EI): m/z (%): 295 (100, M^+), 190 (9), 163 (34). HRMS: m/z [M^+] calcd for $C_{21}H_{13}NO$: 295.0997. Found: 295.0995.

2-Phenylbenzthiazole (9)[12]

98.3 mg (93%). Pale yellow needles. m.p. 122-125 °C, (from Hexane), R_f = 0.6 (Hexane/EtOAc 8:2); ^{1}H NMR (400 MHz, $CDCl_3$): δ = 8.10-8.07 (m, 3H; Ar-H), 7.90 (d, J = 7.2 Hz, 1H; Ar-H), 7.51-7.47 (m, 4H; Ar-H), 7.38 (t, J = 8.4 Hz, 1H; Ar-H) ppm; ^{13}C NMR (100 MHz, $CDCl_3$): δ = 168.2 (C), 154.3 (C), 135.2 (C), 133.7 (C), 131.1 (CH), 129.2 (CH), 127.7 (CH), 126.5 (CH), 125.3 (CH), 123.4 (CH), 121.8 (CH) ppm. MS (EI): m/z (%): 211 (100, M^+), 184 (4), 108 (20). HRMS: m/z [M^+] calcd for $C_{13}H_9NS$: 211.0456. Found: 211.0460.

Methyl 2-(3,5-dichlorophenyl)benzoxazole-6-carboxylate (12)[14]

A mixture of methyl 4-amino-3-hydroxybenzoate (**10**: 0.5 mmol), *N*-phenylthiobenzamide derivative (**11**: 1.0 mmol), and Ph_3BiCl_2 (**6a**: 1.0 mmol) were well stirred at 60 °C in 1,2-DCE (3.0 mL) for 18 hr. After completion of the reaction, the reaction mixture was diluted with H_2O (20 mL) and CH_2Cl_2 (20 mL), and aqueous phase was extracted with CH_2Cl_2 (3 × 30 mL). The combined organic phase was washed with brine (20 mL) and dried over $MgSO_4$. Evaporation of the solvent furnished the crude product. The crude product was then purified by silica gel column chromatography to give desired product (**12**).

148 mg (91%). Colorless prisms. m.p. 165-168 °C (from CH_2Cl_2-Hexane), R_f = 0.40 (Hexane/EtOAc 8:2); ^{1}H NMR (400 MHz, $CDCl_3$): δ = 8.28 (d, J = 0.8 Hz, 1H; Ar-H), 8.16 (d, J = 1.6 Hz, 2H; Ar-H), 8.12 (dd, J = 8.0, 1.2 Hz, 1H; Ar-H), 7.80 (d, J = 8.0 Hz, 1H; Ar-H), 7.55 (t, J = 1.6 Hz, 1H; Ar-H), 3.97 (s, 3H; OMe) ppm; ^{13}C NMR (100 MHz, $CDCl_3$): δ = 166.5 (C), 163.0 (C), 150.6 (C), 145.6 (C), 136.1 (CH), 132.0 (CH), 129.5 (C), 128.1 (C), 126.9 (CH), 126.3 (CH), 120.2 (CH), 112.6 (CH), 52.7 (CH_3) ppm. IR (KBr): $\tilde{\nu}$ = 3078, 2360, 1730, 1546, 1435, 1413, 1286, 1267, 1242, 1230, 987, 860 cm^{-1}. MS (EI): m/z (%): 321 (57, M^+), 290 (100), 234 (30), 145 (18). HRMS: m/z [M^+] calcd for $C_{15}H_9Cl_2NO_3$: 320.9959. Found: 320.9991.

Tafamidis (13)[12]

Benzoxazole derivatives (**12**: 0.5 mmol) was dissolved in a mixture of THF (3.0 mL), MeOH (1.0 mL), and H_2O (1.0 mL) and treated with anhydrous LiOH (2.0 mmol) at room temperature for 12 h. The reaction mixture was acidified to pH 4.0 with 1N HCl aq. and extracted with EtOAc. The combined organic layer was dried over $MgSO_4$, filtered and concentrated to give the product **13**.

140 mg (92%). Colorless prisms. m.p. 260 °C (decomp. from THF-H_2O), R_f = 0.2 (EtOAc). ^{1}H NMR (400 MHz, DMSO-d_6): δ = 13.2 (brs, 1H; OH), 8.25 (d, J = 1.2 Hz, 1H; Ar-H), 8.12 (d, J = 2.4 Hz, 2H; Ar-H), 8.00 (dd, J = 7.6, 1.2 Hz, 1H; Ar-H), 7.94-7.90 (m, 1H; Ar-H), 7.89 (d, J = 8.8 Hz, 1H; Ar-H) ppm; ^{13}C NMR (100 MHz, DMSO-d_6): δ = 167.2 (C), 162.6 (C), 150.6 (C),

145.3 (C), 135.8 (C), 132.3 (CH), 129.8 (C), 129.3 (C), 127.1 (CH), 126.5 (CH), 120.6 (CH), 112.8 (CH) ppm. IR (KBr): $\tilde{v}$ = 2924, 2360, 1697, 1546, 1437, 1419, 1307, 1296, 1273, 862, 773 cm^{-1}. HRMS: *m/z* [M-H$^-$] calcd for $C_{14}H_6Cl_2NO_3$: 305.9730. Found: 305.9734.

4. Synthesis of 2-substituted benzimidazoles.

A mixture of *N*-tosyl-*o*-phenylenediamine (**15**: 0.5 mmol), *N*-phenylthiobenzamide (**2**: 1.0 mmol), and Ph_3BiCl_2 (**6a**: 1.0 mmol) were well stirred at 60 °C in 1,2-DCE (3.0 mL) for 18 h. After completion of the reaction, the reaction mixture was diluted with H_2O (20 mL) and CH_2Cl_2 (20 mL), and aqueous phase was extracted with CH_2Cl_2 (3 × 30 mL). The combined organic phase was washed with brine (20 mL) and dried over $MgSO_4$. Evaporation of the solvent furnished the crude product. The crude product was then purified by column chromatography on silica gel.

2-(4-Methoxyphenyl)-1-tosyl-1*H*-benzimidazole (16b)[15]

143 mg (76%). Colorless plates. m.p. 119-122 °C (from CH_2Cl_2-Hexane), R_f = 0.4 (Hexane/EtOAc 7:3). ^{1}H NMR (400 MHz, $CDCl_3$): δ = 8.18 (dd, *J* = 8.8, 2.0 Hz, 1H; Ar-H), 7.68 (dd, *J* = 7.6, 0.8 Hz, 1H; Ar-H), 7.58 (dt, *J* = 8.8, 2.8 Hz, 2H; Ar-H), 7.42-7.34 (m, 2H; Ar-H), 7.29 (d, *J* = 8.4 Hz, 2H; Ar-H), 7.07 (d, *J* = 8.0 Hz, 2H; Ar-H), 6.97 (dt, *J* = 8.8, 2.4 Hz, 2H; Ar-H), 3.89 (s, 3H; OMe), 2.30 (s, 3H; Me) ppm; ^{13}C NMR (100 MHz, $CDCl_3$): δ = 161.6 (C), 154.4 (C), 145.7 (C), 142.9 (C), 135.1 (C), 134.1 (C), 132.6 (CH), 129.8 (CH), 127.1 (CH), 125.4 (CH), 122.3 (C), 120.3 (CH), 115.5 (CH), 113.2 (CH), 55.6 (CH_3), 21.8 (CH_3) ppm. IR (KBr): $\tilde{v}$ = 3007, 2360, 1600, 1491, 1440, 1390, 1249, 1190, 1174, 1074, 1022, 1003, 833 cm^{-1}. MS (EI): *m/z* (%): 378 (20), 223 (100), 180 (9), 133 (17). HRMS: *m/z* [M$^+$] calcd for $C_{21}H_{18}N_2O_3S$: 378.1038. Found: 378.1042.

2-Phenyl-1-tosyl-1*H*-benzimidazole (16c)[15]

148 mg (85%). Colorless needles. m.p. 100-103 °C (from CH_2Cl_2-Hexane), R_f = 0.4 (Hexane/EtOAc 7:3). ^{1}H NMR (400 MHz, $CDCl_3$): δ = 8.19 (d, *J* = 7.6 Hz, 1H; Ar-H), 7.72 (d, *J* = 7.6 Hz, 1H, Ar-H), 7.61-7.58 (m, 2H; Ar-H), 7.56-7.54 (m, 1H; Ar-H), 7.49-7.36 (m, 4H; Ar-H), 7.31 (d, *J* = 8.4 Hz, 2H; Ar-H), 7.08 (d, *J* = 8.0 Hz, 2H; Ar-H), 2.32 (s, 3H; Me) ppm; ^{13}C NMR (100 MHz, $CDCl_3$): δ = 154.2 (C), 145.8 (C), 142.7 (C), 135.1 (C), 134.0 (C), 131.0 (CH), 130.6 (CH), 130.2 (C), 129.8 (CH), 127.8 (CH), 127.1 (CH), 125.6 (CH), 125.4 (CH), 120.5 (CH), 115.3 (CH), 21.7 (CH_3) ppm. IR (KBr): $\tilde{v}$ = 3055, 2360, 1597, 1448, 1388, 1303, 1192, 1176, 1120, 1084, 1033, 1010, 769 cm^{-1}. MS (EI): *m/z* (%): 348 (40, M$^+$), 193 (100), 90 (28). HRMS: *m/z* [M$^+$] calcd for $C_{20}H_{16}N_2O_2S$: 348.0932. Found: 348.0935.

2-[(4-Trifluoromethyl)phenyl]-1-tosyl-1*H*-benzimidazole (16d)

165 mg (79%). Pale yellow needles. m.p. 167-169 °C (from CH_2Cl_2-Hexane), R_f = 0.4 (Hexane/EtOAc 8:2). ^{1}H NMR (400 MHz, $CDCl_3$): δ = 8.19 (d, J = 8.0 Hz, 1H; Ar-H), 7.77-7.71 (m, 5H; Ar-H), 7.46 (td, J = 8.0, 7.2, Hz, 1H; Ar-H), 7.41 (t, J = 7.2 Hz, 1H; Ar-H), 7.33 (d, J = 8.8 Hz, 2H; Ar-H), 7.11 (d, J = 8.4 Hz, 2H; Ar-H), 2.33 (s, 3H; Me) ppm; ^{13}C NMR (100 MHz, $CDCl_3$): δ = 152.5 (C), 146.2 (C), 142.7 (C), 134.9 (C), 133.9 (C), 133.7 (C), 132.4 (q; $^2J_{C,F}$ = 32.6 Hz, C), 131.4 (CH), 130.0 (CH), 127.0 (CH), 126.1 (CH), 125.7 (CH), 124.7 (q; $^3J_{C,F}$ = 3.4 Hz, CH), 124.1 (q; $^1J_{C,F}$ = 271 Hz, C), 120.8 (CH), 115.3 (CH), 21.8 (CH_3) ppm; ^{19}F NMR (376 MHz, $CDCl_3$): δ = -62.7 (s) ppm. IR (KBr): $\tilde{\nu}$ = 3057, 2360, 1448, 1410, 1384, 1325, 1305, 1274, 1188, 1176, 1159, 1109, 1076, 1062, 850 cm^{-1}. MS (EI): *m/z* (%): 461 (47, M^+), 261 (34), 155 (82), 91 (100). HRMS: *m/z* [M^+] calcd for $C_{21}H_{15}F_3N_2O_2S$: 416.0806. Found: 416.0804.

2-*o*-Tolyl-1-tosyl-1*H*-benzimidazole (16e)[16]

158 mg (87%). Colorless needles. m.p. 153-155 °C (from CH_2Cl_2-Hexane), R_f = 0.3 (Hexane/EtOAc 8:2). ^{1}H NMR (400 MHz, $CDCl_3$): δ = 8.22 (d, J = 8.8 Hz, 1H; Ar-H), 7.75 (d, J = 6.8 Hz, 1H; Ar-H), 7.47-7.38 (m, 5H; Ar-H), 7.26-7.21 (m, 2H; Ar-H), 7.16 (t, J = 8.4 Hz, 3H; Ar-H), 2.36 (s, 3H; Me), 2.05 (s, 3H; Me) ppm; ^{13}C NMR (100 MHz, $CDCl_3$): δ = 152.5 (C), 146.0 (C), 142.4 (C), 139.1 (C), 135.4 (C), 133.3 (C), 130.8 (CH), 130.5 (CH), 130.0 (C), 129.94 (CH), 129.87 (CH), 127.5 (CH), 125.5 (CH), 125.0 (CH), 124.9 (CH), 120.6 (CH), 114.6 (CH), 21.8 (CH_3), 20.0 (CH_3) ppm. IR (KBr): $\tilde{\nu}$ = 2920, 2342, 1543, 1450, 1365, 1303, 1269, 1255, 1192, 1174, 1072, 767 cm^{-1}. MS (EI): *m/z* (%): 362 (3, M^+), 298 (21), 207 (100), 91 (14). HRMS: *m/z* [M^+] calcd for $C_{21}H_{18}N_2O_2S$: 362.1089. Found: 362.1092.

2-Mesityl-1-tosyl-1*H*-benzimidazole (16f)[15]

146 mg (75%). Colorless prisms. m.p. 162-165 °C (from Hexane), R_f = 0.5 (Hexane/EtOAc 8:2). ^{1}H NMR (400 MHz, $CDCl_3$): δ = 8.26 (d, J = 7.2 Hz, 1H; Ar-H), 7.76 (d, J = 6.8 Hz, 1H; Ar-H), 7.48-7.38 (m, 4H; Ar-H), 7.17 (d, J = 8.4 Hz, 2H; Ar-H), 6.87 (s, 2H; Ar-H), 6.87 (s, 2H; Ar-H), 2.38 (s, 3H; Me), 2.36 (s, 3H; Me), 1.75 (s, 6H; Me) ppm; ^{13}C NMR (100 MHz, $CDCl_3$): δ = 151.9 (C), 146.0 (C), 142.4 (C), 140.0 (C), 138.8 (C), 135.3 (C), 133.2 (C), 129.8 (CH), 128.0 (CH), 127.8 (CH), 126.9 (C), 125.3 (CH), 124.7 (CH), 120.6 (CH), 114.3 (CH), 21.9 (CH_3), 21.6 (CH_3), 20.0 (CH_3) ppm. IR (KBr): $\tilde{\nu}$ = 2920, 2359, 1610, 1545, 1448, 1383, 1251, 1230, 1190, 1180, 1165, 1124, 1091, 1060, 1014, 812 cm^{-1}. MS (EI): *m/z* (%): 390 (4, M^+), 326 (22), 235 (100), 91 (11). HRMS: *m/z* [M^+] calcd for $C_{23}H_{22}N_2O_2S$: 390.1402. Found: 390.1405.

2-(Thiophen-2-yl)-1-tosyl-1*H*-benzimidazole (16g)

124 mg (70%). Colorless oil. R_f = 0.4 (Hexane/EtOAc 8:2). ^{1}H NMR (400 MHz, $CDCl_3$): δ = 8.20 (d, J = 8.0 Hz, 1H; Ar-H), 7.80 (d, J = 3.2 Hz, 1H; Ar-H), 7.70 (dd, J = 5.6, 2.0 Hz, 1H; Ar-H), 7.53 (d, J = 4.8 Hz, 1H; Ar-H), 7.43-7.34 (m, 4H; Ar-H), 7.18 (dd, J = 3.6, 1.2 Hz, 1H; Ar-H), 7.08 (d, J = 8.4 Hz, 2H; Ar-H), 2.31 (s, 3H; Me) ppm; ^{13}C NMR (100 MHz, $CDCl_3$): δ = 147.9 (C), 145.9 (C), 142.6 (C), 134.8 (C), 134.3 (C), 133.4 (CH), 130.2 (C), 130.0 (CH), 129.9 (CH), 127.6 (CH), 127.0 (CH), 125.7 (CH), 125.6 (CH), 120.4 (CH), 115.5 (CH), 21.8 (CH_3) ppm. IR (neat): $\tilde{\nu}$ = 3074, 2357, 1556, 1487, 1448, 1417, 1387, 1298, 1273, 1255, 1232, 1215, 1151, 1120, 1089, 1062, 1041 cm^{-1}. MS (EI): *m/z* (%): 354 (25, M^+), 199 (100), 172 (16), 90 (27). HRMS: *m/z* [M^+] calcd for $C_{18}H_{14}N_2O_2S_2$: 354.0497. Found: 354.0495.

2-Cyclohexyl-1-tosyl-1*H*-benzimidazole (16h)[15]

127 mg (72%). Colorless prisms. m.p. 125-128 °C (from Hexane), R_f = 0.5 (Hexane/EtOAc 8:2). ^{1}H NMR (400 MHz, $CDCl_3$): δ = 8.05-8.03 (m, 1H; Ar-H), 7.74 (d, J = 8.0 Hz, 2H; Ar-H), 7.67-7.65 (m, 1H; Ar-H), 7.34-7.30 (m, 2H; Ar-H), 7.26 (d, J = 8.4 Hz, 2H; Ar-H), 3.51-3.45 (m, 1H; Cy), 2.38 (s, 3H; Me), 1.93-1.83 (m, 4H; Cy), 1.77-1.62 (m, 3H; Cy), 1.46-1.30 (m, 3H; Cy) ppm; ^{13}C NMR (100 MHz, $CDCl_3$): δ = 159.8 (C), 145.9 (C), 142.2 (C), 136.0 (C), 133.0 (C), 130.3 (CH), 126.7 (CH), 124.8 (CH), 124.7 (CH). 119.9 (C), 114.1 (C), 38.3 (CH), 32.7 (CH_2), 26.5 (CH_2), 25.9 (CH_2), 21.8 (CH_3) ppm. IR (KBr): $\tilde{\nu}$ = 2929, 2848, 2359, 1597, 1452, 1369, 1246, 1228, 1193, 1165, 1120, 1045, 744 cm^{-1}. MS (EI): *m/z* (%): 354 (4, M^+), 286 (13), 199 (100), 145 (20). HRMS: *m/z* [M^+] calcd for $C_{20}H_{22}N_2O_2S$: 354.1402. Found: 354.1439.

2-Methyl-1-tosyl-1*H*-benzimidazole (16i)[17]

105 mg (73%). Colorless prisms. m.p. 122-124 °C (from Acetone-Hexane), R_f = 0.4 (Hexane/EtOAc 7:3). ^{1}H NMR (400 MHz, $CDCl_3$): δ = 8.02-8.00 (m, 1H; Ar-H), 7.80 (d, J = 8.8 Hz, 2H; Ar-H), 7.62-7.60 (m, 1H; Ar-H), 7.36-7.27 (m, 4H; Ar-H), 2.80 (s, 3H; Me), 2.38 (s, 3H; Me) ppm; ^{13}C NMR (100 MHz, $CDCl_3$): δ = 151.5 (C), 146.1 (C), 142.0 (C), 135.5 (C), 133.3 (C), 130.4 (CH), 126.9 (CH), 124.9 (CH), 124.7 (CH), 119.8 (CH), 113.6 (CH), 21.8 (CH_3), 17.1 (CH_3) ppm. IR (KBr): $\tilde{\nu}$ = 2924, 2360, 1545, 1454, 1384, 1371, 1247, 1172, 1149, 1087, 999 cm^{-1}. MS (EI): *m/z* (%): 286 (63), 155 (57), 131 (17), 91 (100). HRMS: *m/z* [M^+] calcd for $C_{15}H_{14}N_2O_2S$: 286.0776. Found: 286.0780.

5. References

1. Xu, Z-M.; Li, H-X.; Young, D. J.; Zhu D-L.; Li, H-Y.; Lang, J-P. *Org. Lett.* **2019**, *21,* 237–241.
2. Saha, S.; Moorthy, J. N. *Tetrahedron Lett.* **2010**, *51,* 912–916.
3. Bresien, J.; Hering-Junghans, C.; Schulz, A.; Thomas, M.; Villinger, A. *Organometallics* **2018**, *37*, 2571–2580.
4. Moiseev, M. D.; Malysheva, Y. B.; Shavyrin, A. S.; Kurskii, Y. A.; Gushchin, A. V. *J. Organomet. Chem*., **2005**, *690*, 3652–3663.
5. Ueda, S.; Nagasawa, H. *Angew. Chem. Int. Ed.* **2008**, *47,* 6411–6413.
6. Shi, W-M.; Li, X-H.; Liang, C.; Mo, D-L. *Adv. Synth. Catal.* **2017**, *359,* 4129–4135.
7. Layek, S.; Agrahari, B.; Kumar, A.; Dege, N.; Pathak, D. D. *Inorg. Chim. Acta.* **2020**, *500,* 119222.
8. Liu, N.; Wang, Z-X. *J. Org. Chem.* **2011**, *76,* 10031–10038.
9. Zhou, Q.; Liu, S.; Ma, M.; Cui, H-Z.; Hong, X.; Huang, S.; Zhang, J-F.; Hou, X-F. *Synthesis* **2018**, *50,* 1315–1322.
10. Kantam, M. L.; Venkanna, G. T.; Kumar, K. B. S.; Balasubrahmanyam, V.; Bhargava, S. *Synlett* **2009**, *11,* 1753–1756.
11. Li, K-L.; Du, Z-B.; Guo, C-C.; Chen, Q-Y. *J. Org. Chem.* **2009**, *74,* 3286–3292.
12. Shi, Y.; Zhou, Q.; Du, F.; Fu, Y.; Du, Y.; Fang, T.; Chen, G. *Tetrahedron Lett.* **2019**, *60,* 151082.
13. Boissarie, P. J.; Hamilton, Z. E.; Lang, S.; Murphy, J. A.; Suckling, C. J. *Org. Lett.* **2011**, *13,* 6256–6259.
14. Kumar, D.; Pilania, M.; Arun, V.; Pooniya, S. *Org. Biomol. Chem.* **2014**, *12,* 6340–6344.
15. Hu, Z.; Zhao, T.; Wang, M.; Wu, J.; Yu, W.; Chang, J. *J. Org. Chem.* **2017**, *82,* 3152–3158.
16. Xu, L.; Wang, L.; Feng, Y.; Li, Y.; Yang, L.; Cui, X. *Org. Lett.* **2017**, *19,* 4343–4346.
17. Kljajic, M.; Puschnig, J. G.; Weber, H.; Breinbauer, R. *Org. Lett.* **2017**, *19,* 126–129.

6. Copies of ^{1}H, ^{13}C and ^{19}F NMR spectra

^{1}H NMR of **8a**

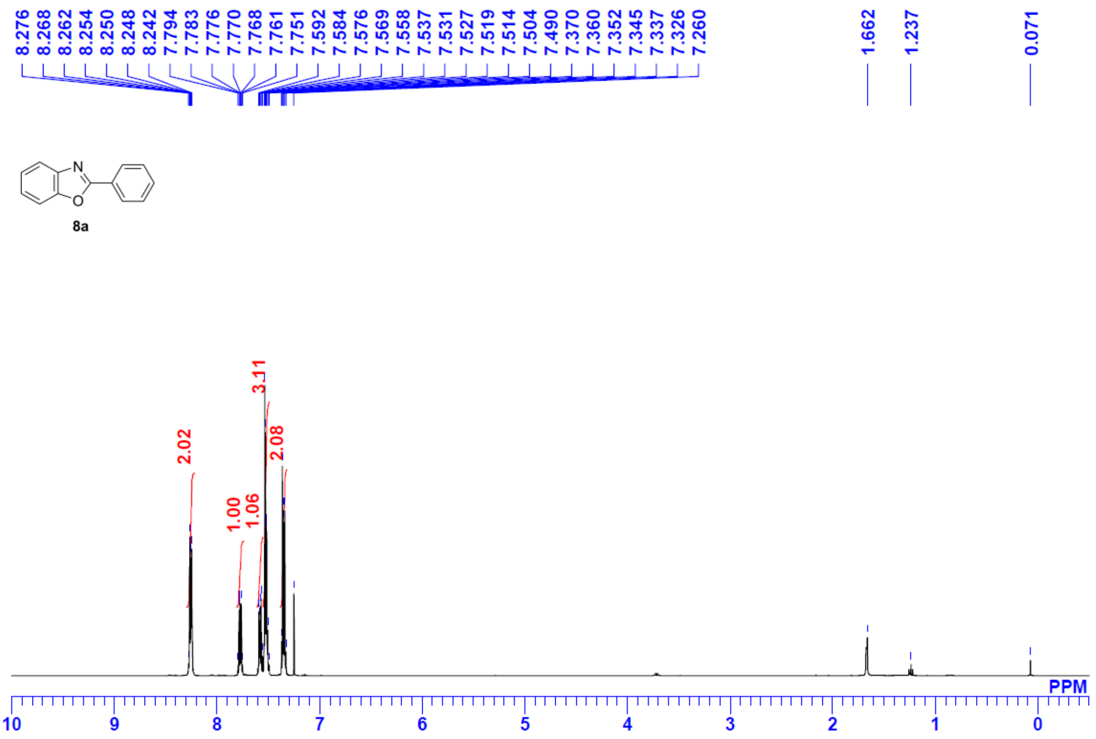

^{13}C NMR of **8a**

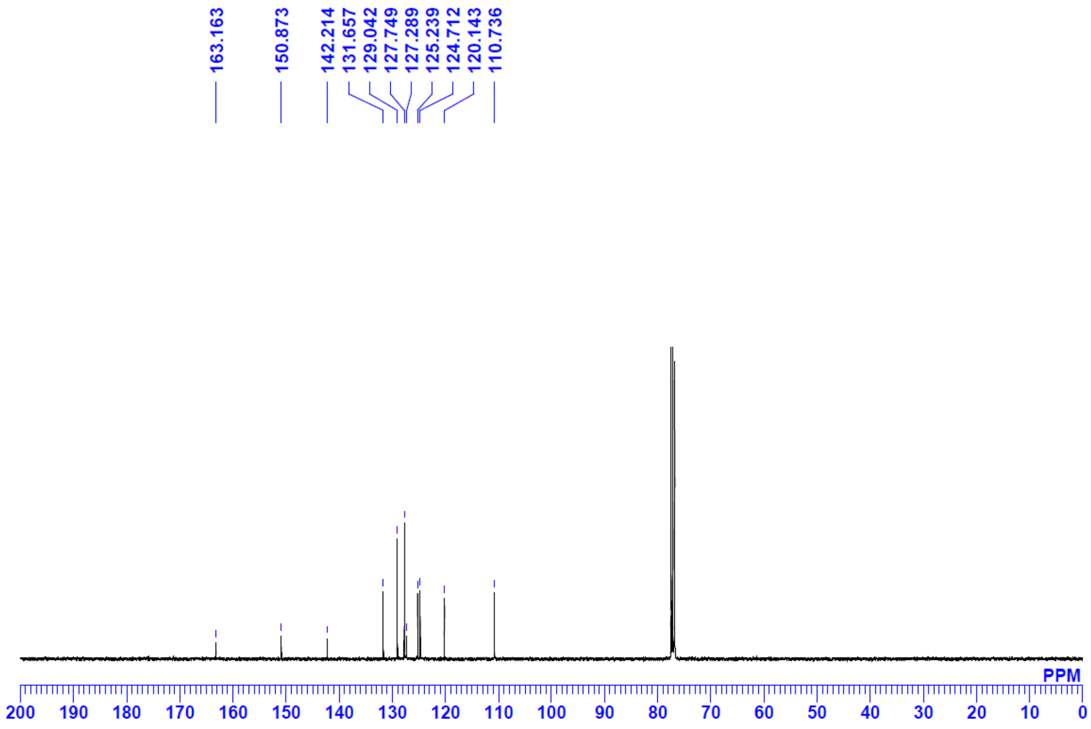

^{1}H NMR of **8b**

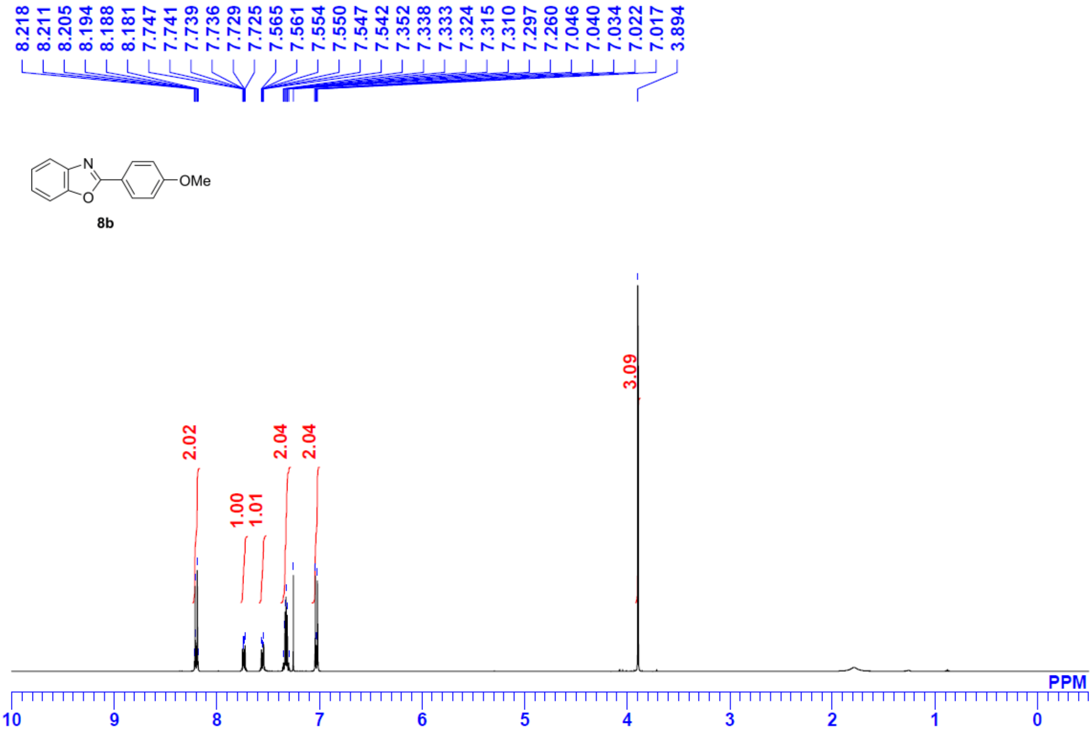

^{13}C NMR of **8b**

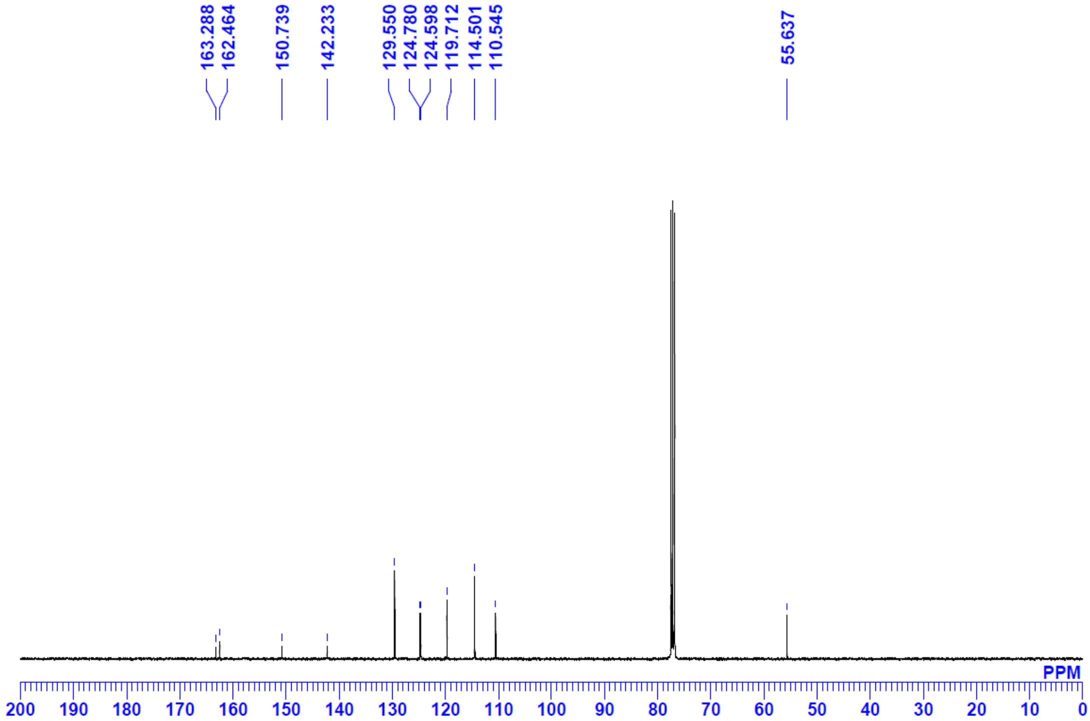

^{1}H NMR of **8c**

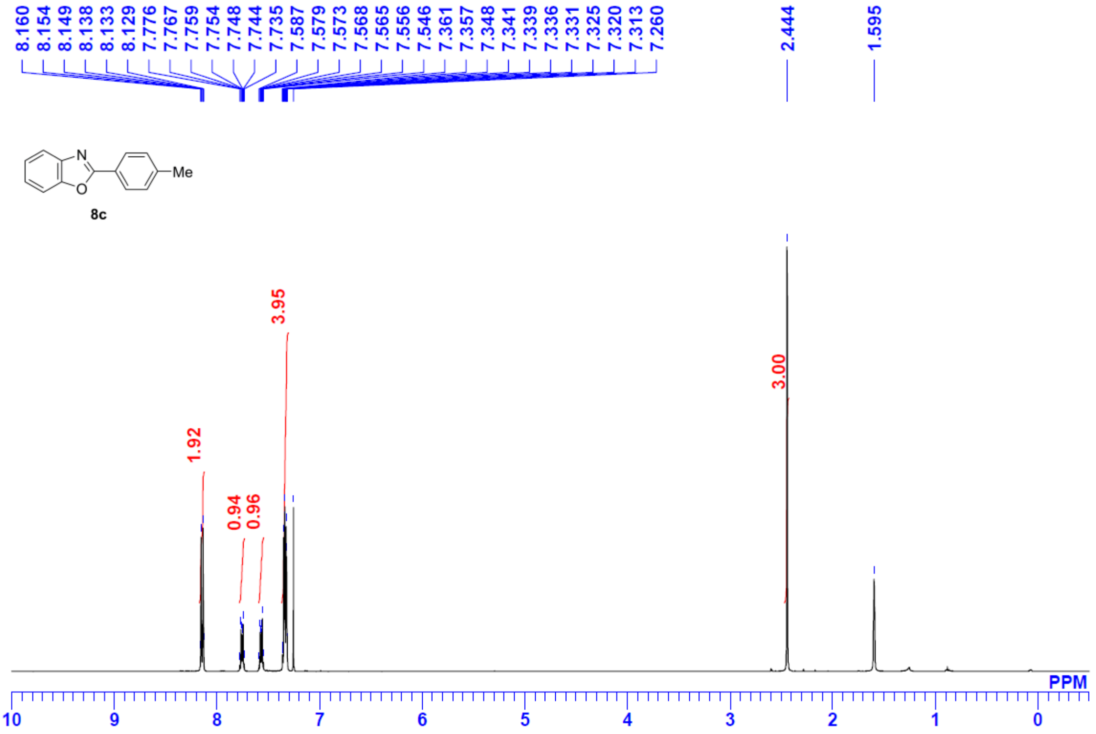

^{13}C NMR of **8c**

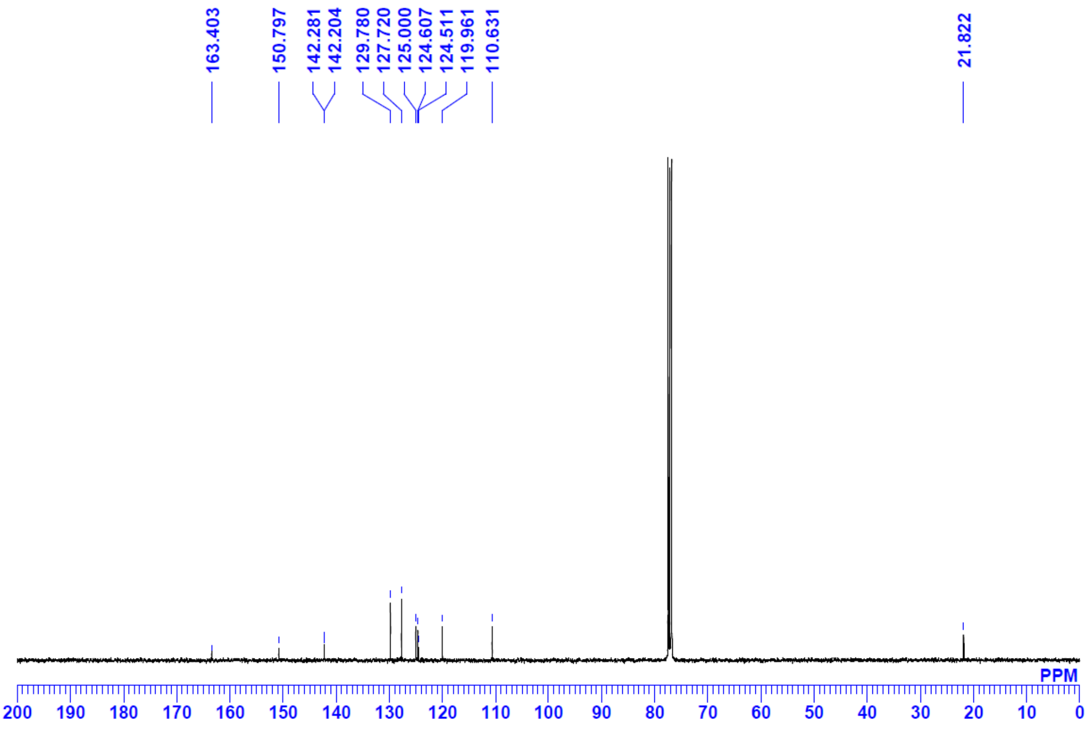

^{1}H NMR of **8d**

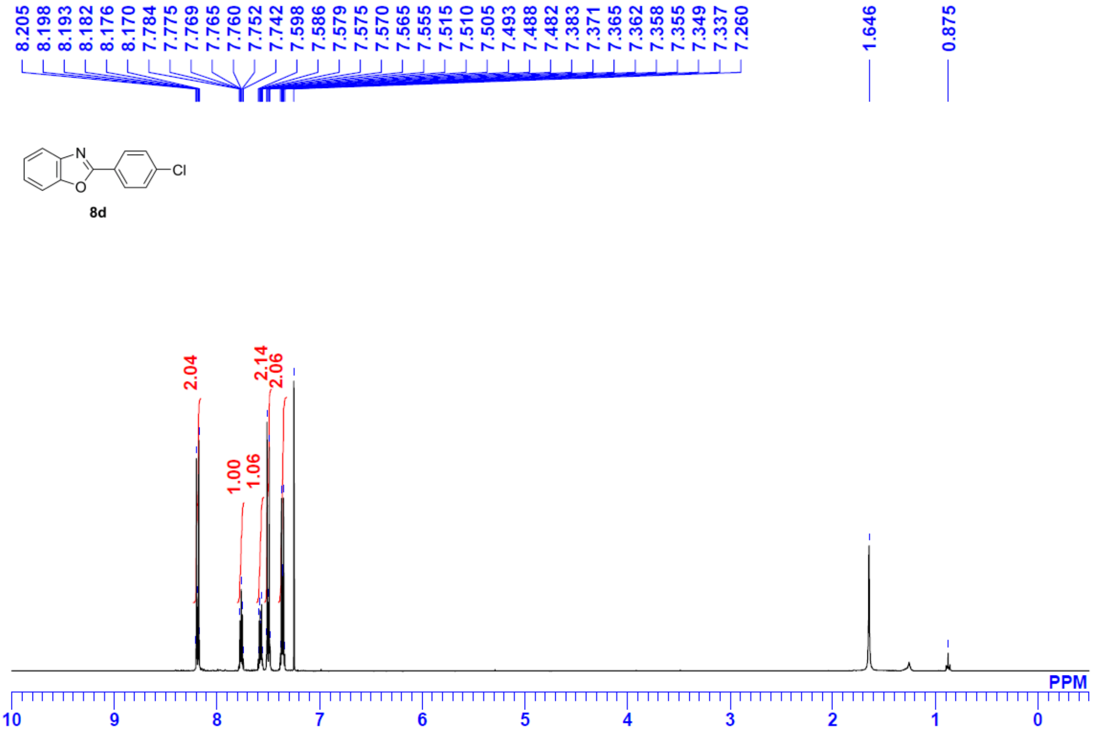

^{13}C NMR of **8d**

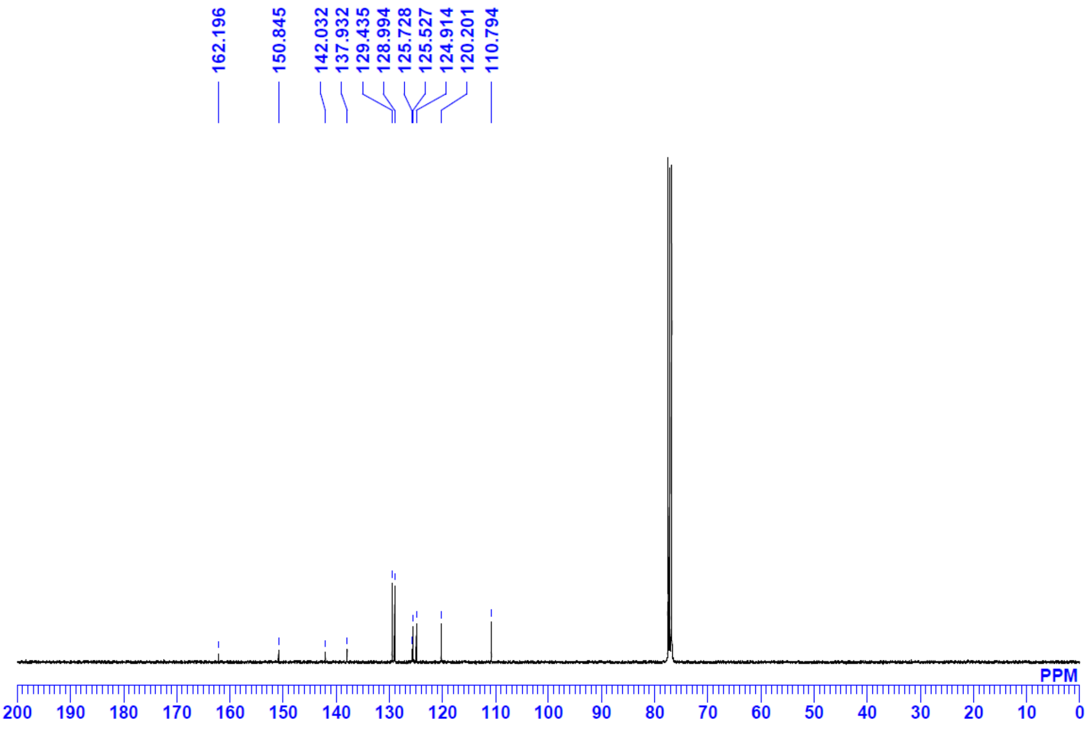

^{1}H NMR of **8e**

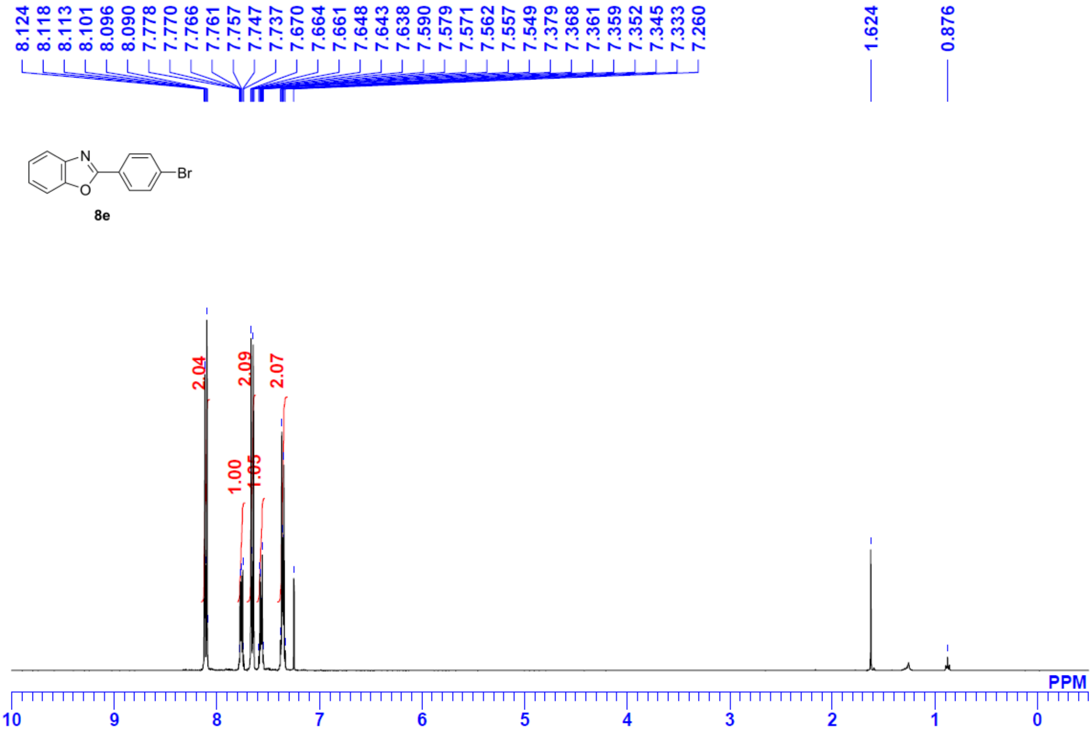

^{13}C NMR of **8e**

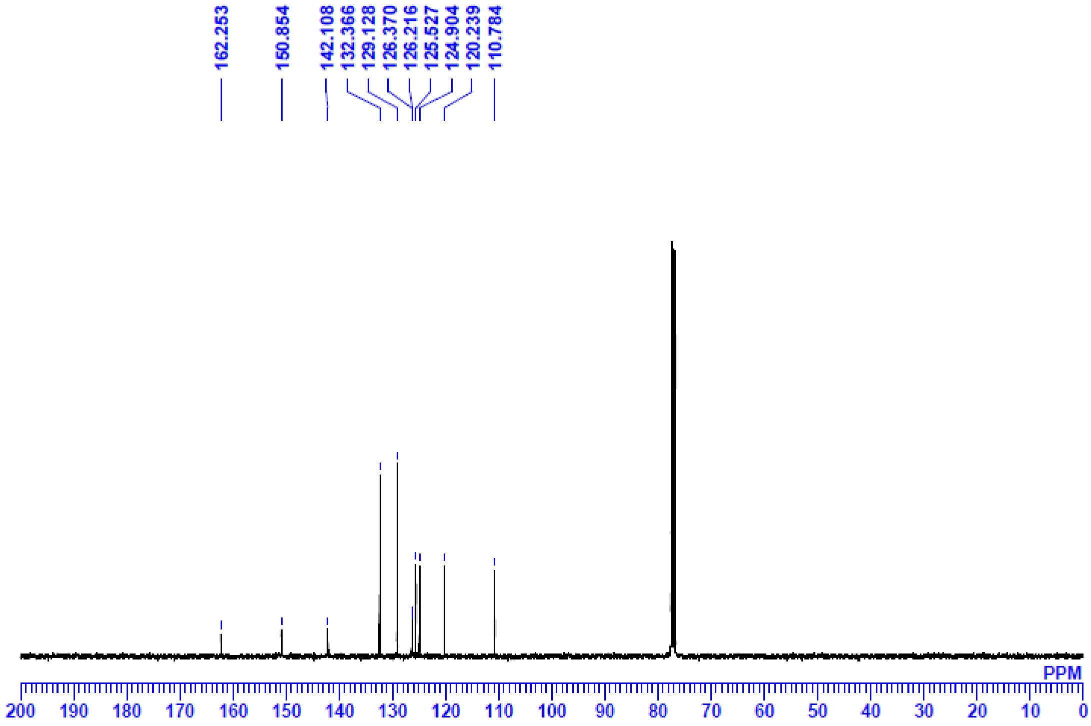

^{1}H NMR of **8f**

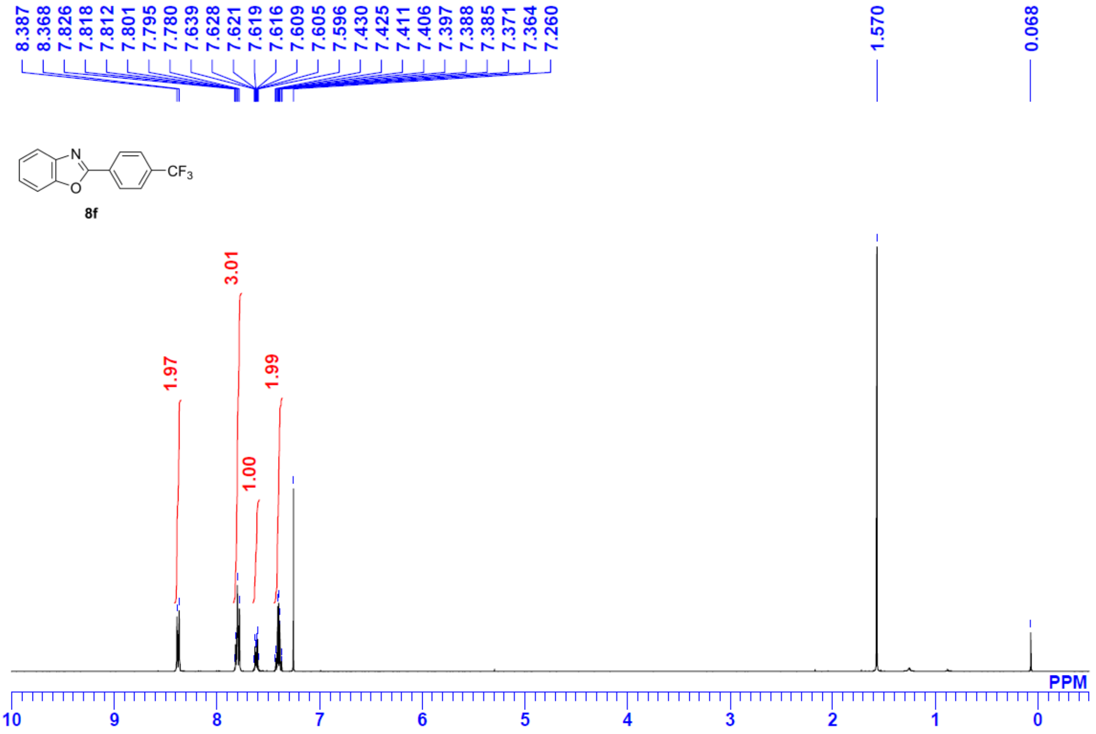

^{13}C NMR of **8f**

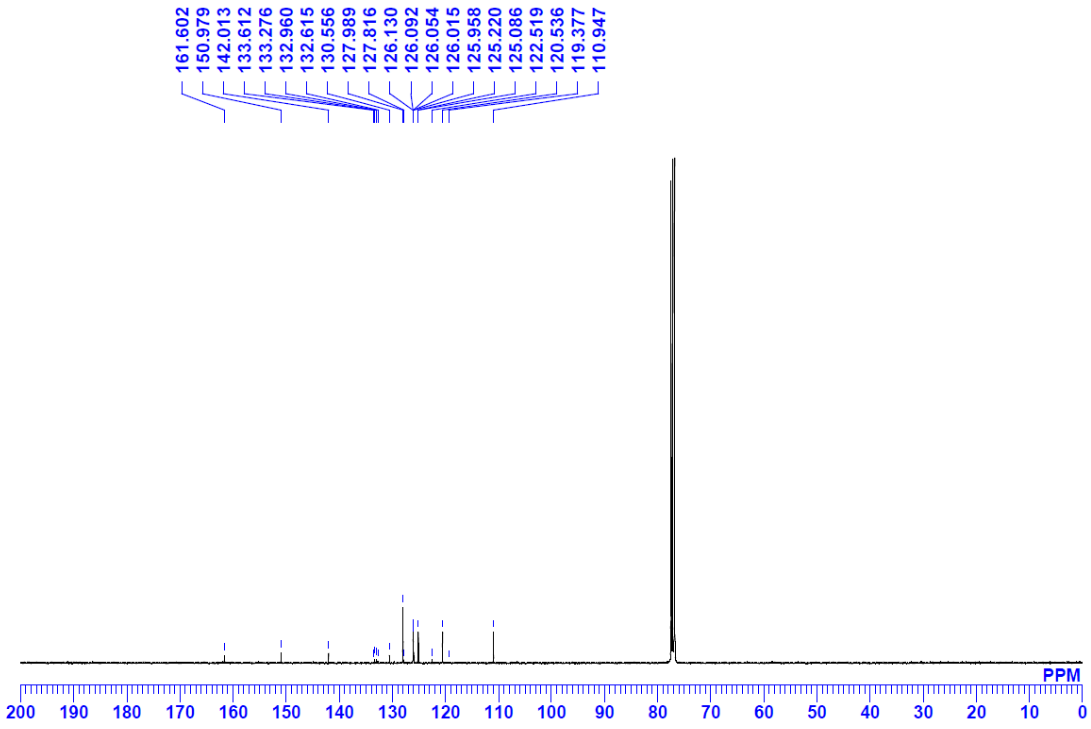

^{19}F NMR of **8f**

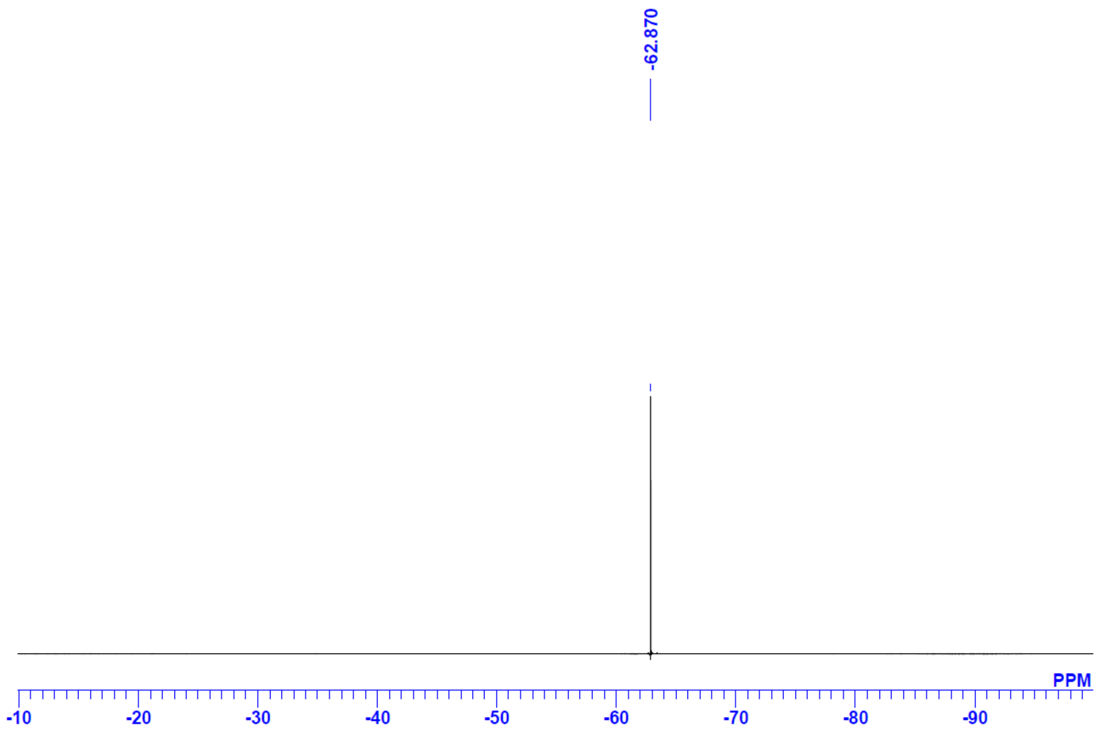

^{1}H NMR of **8g**

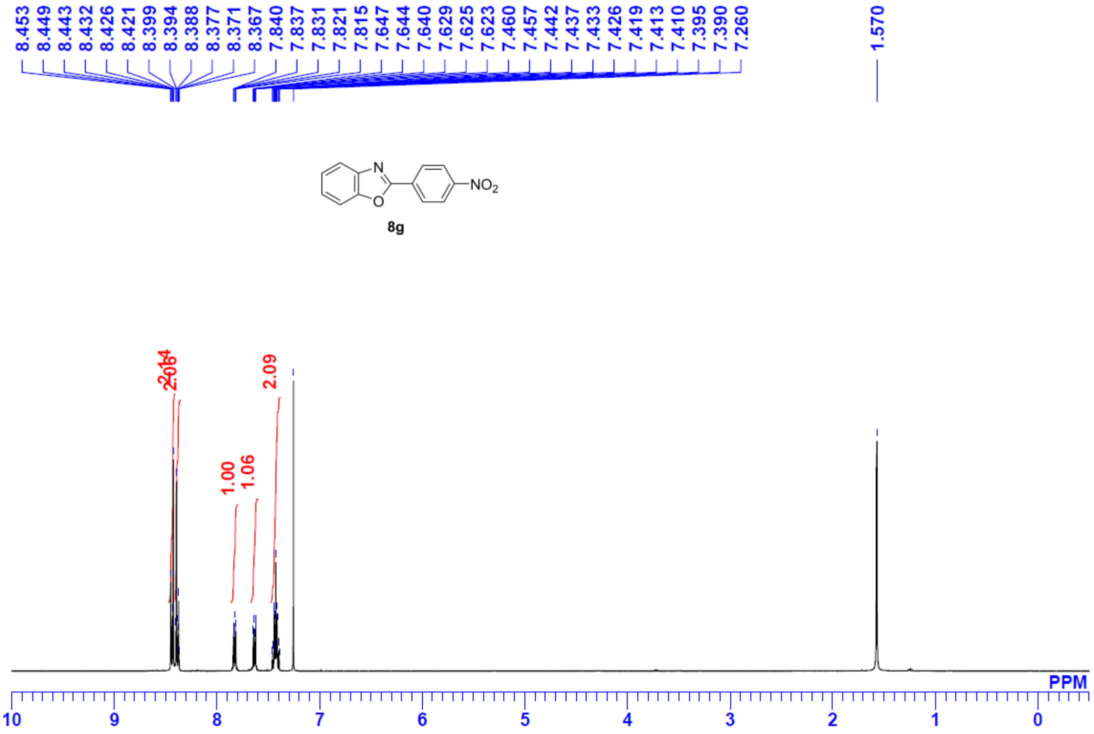

^{13}C NMR of **8g**

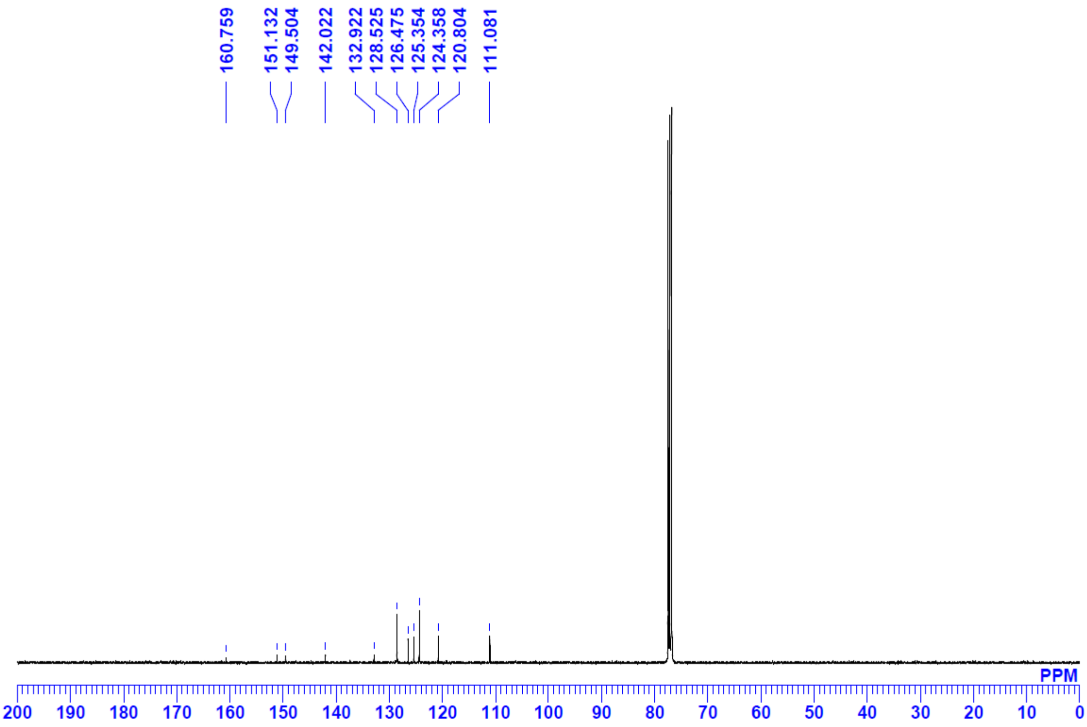

^{1}H NMR of **8h**

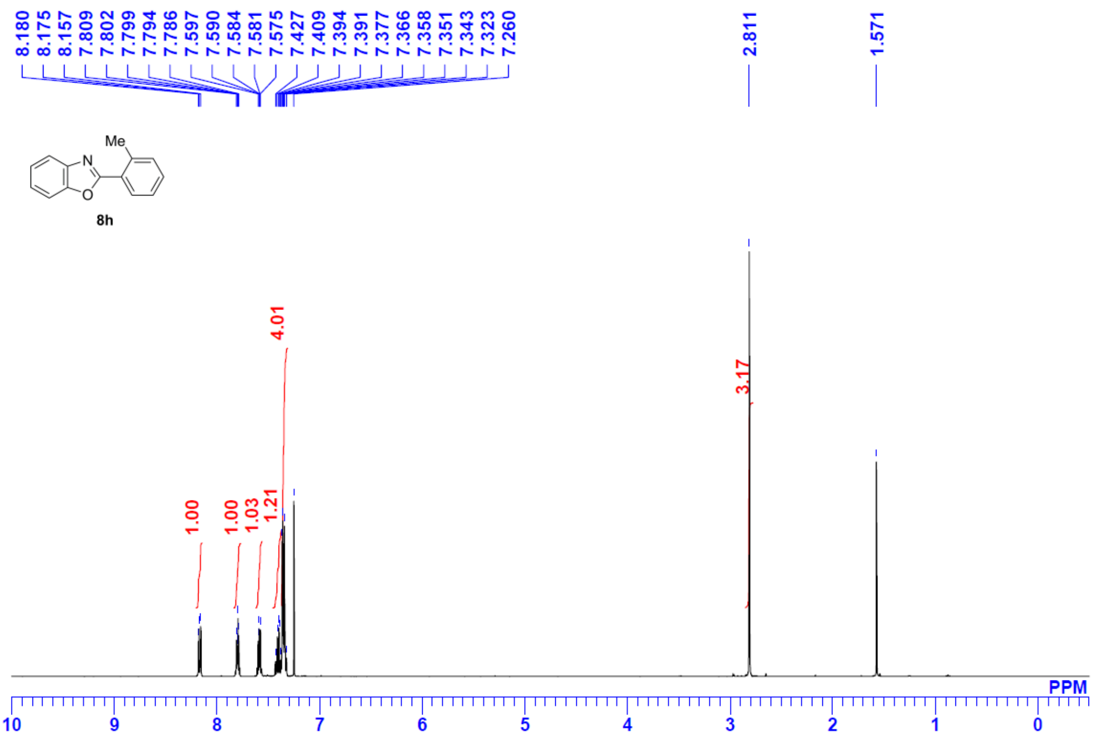

^{13}C NMR of **8h**

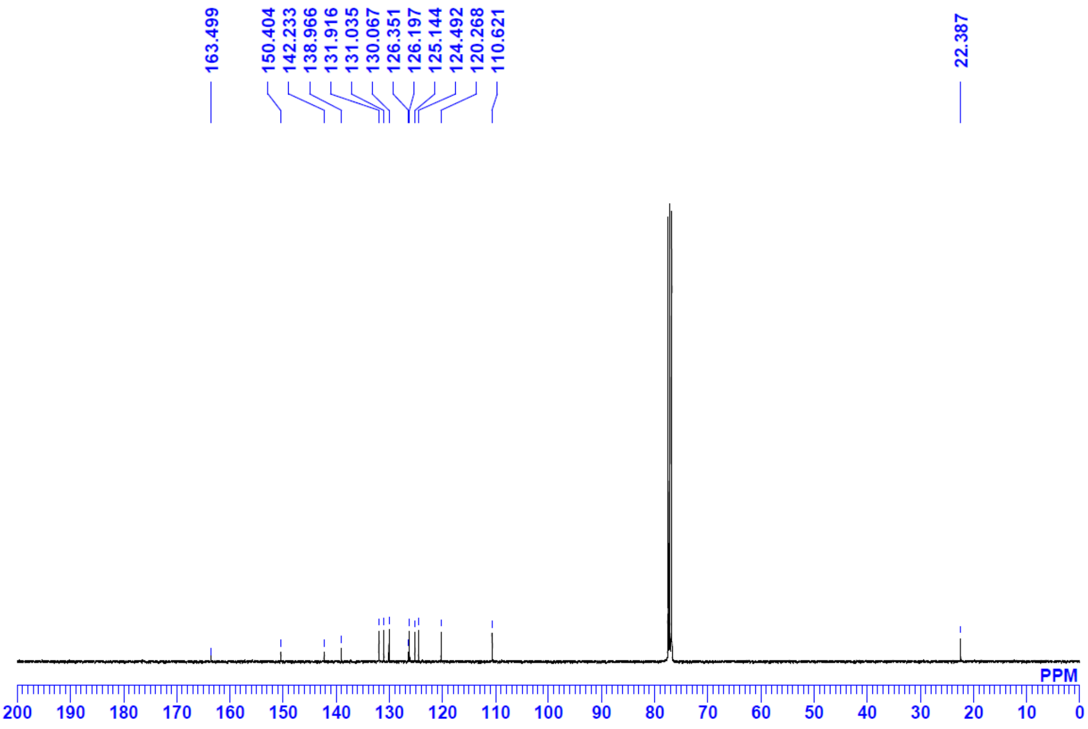

^{1}H NMR of **8i**

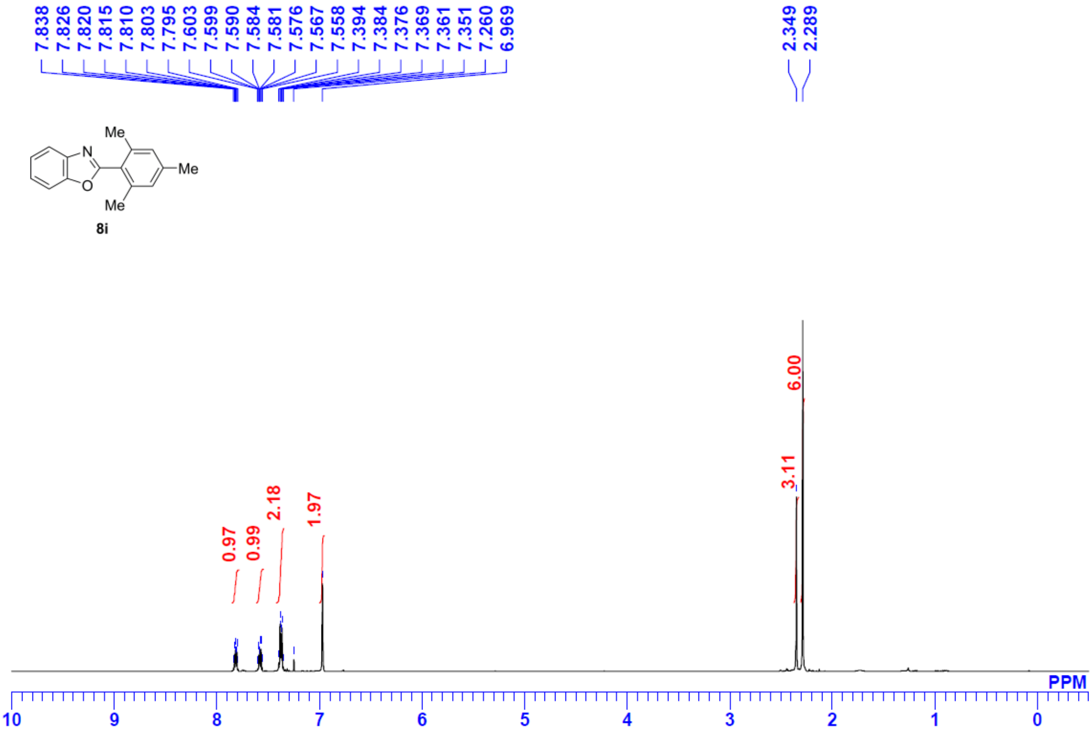

^{13}C NMR of **8i**

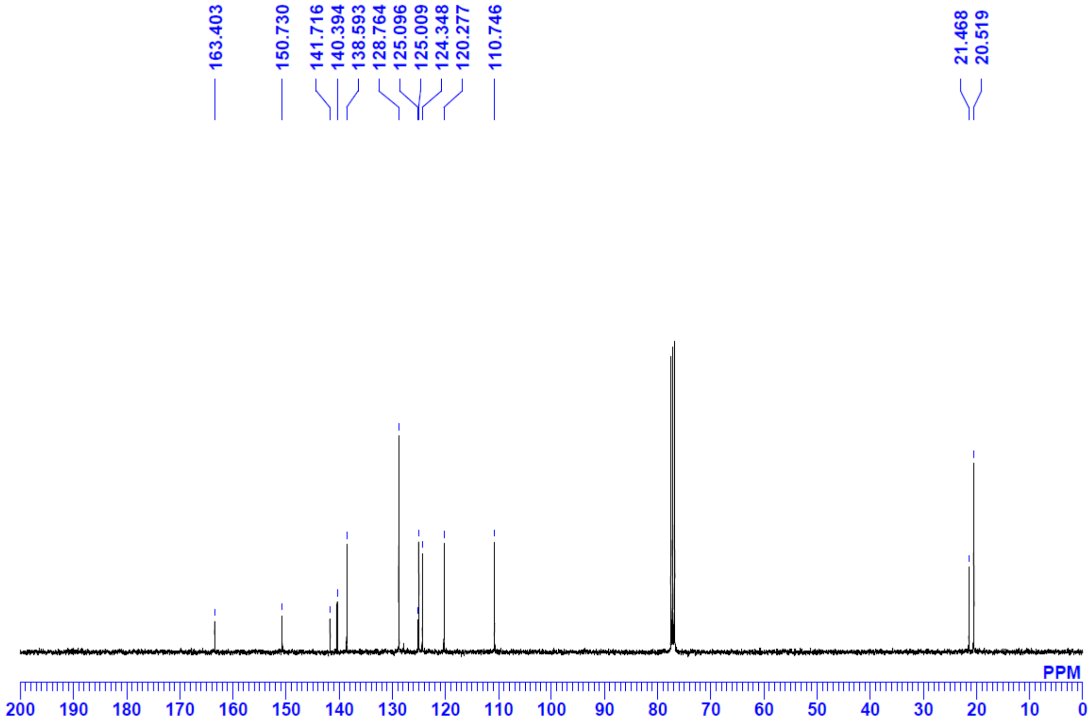

^{1}H NMR of **8j**

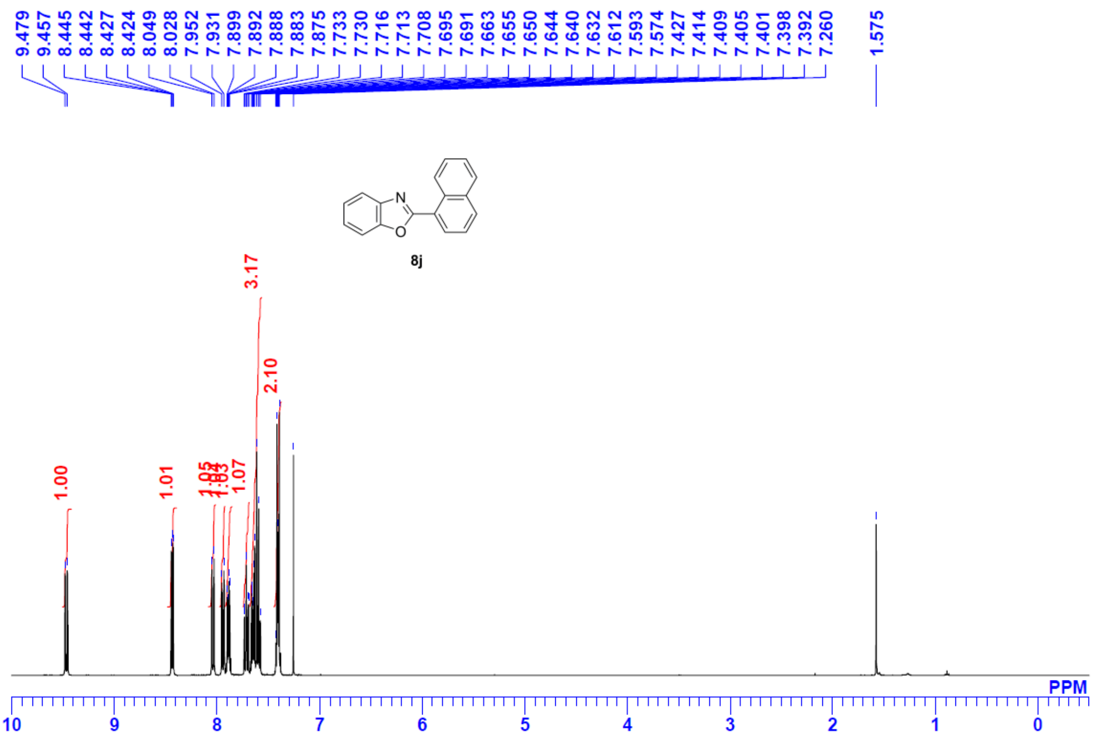

^{13}C NMR of **8j**

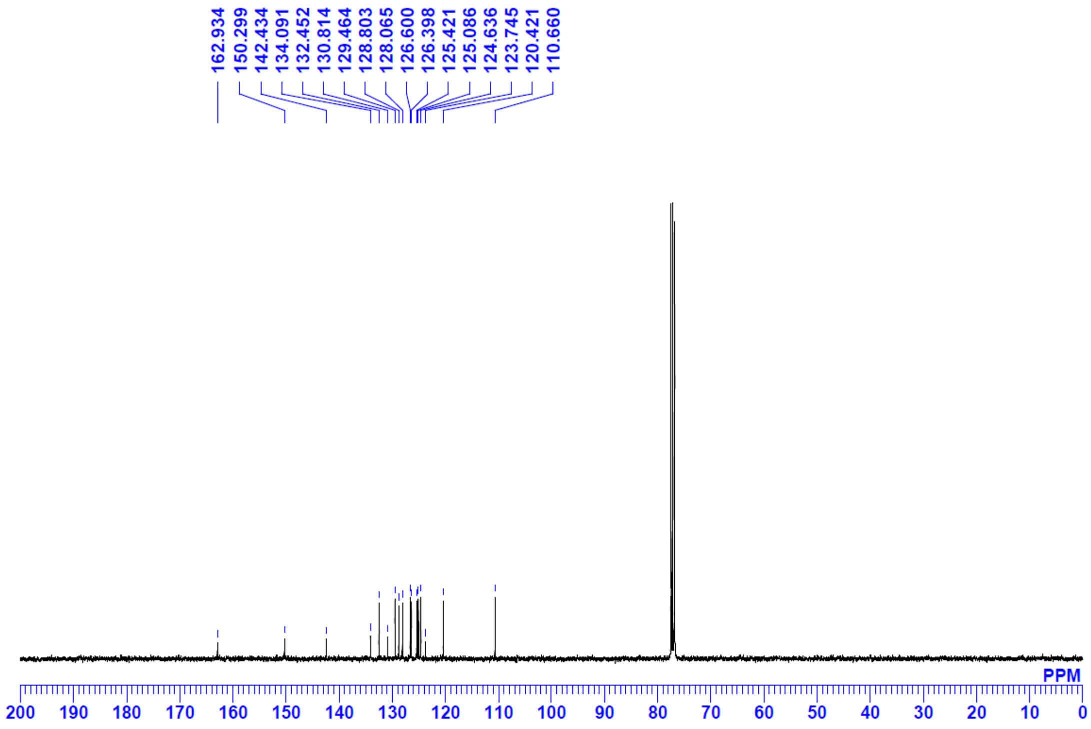

^{1}H NMR of **8k**

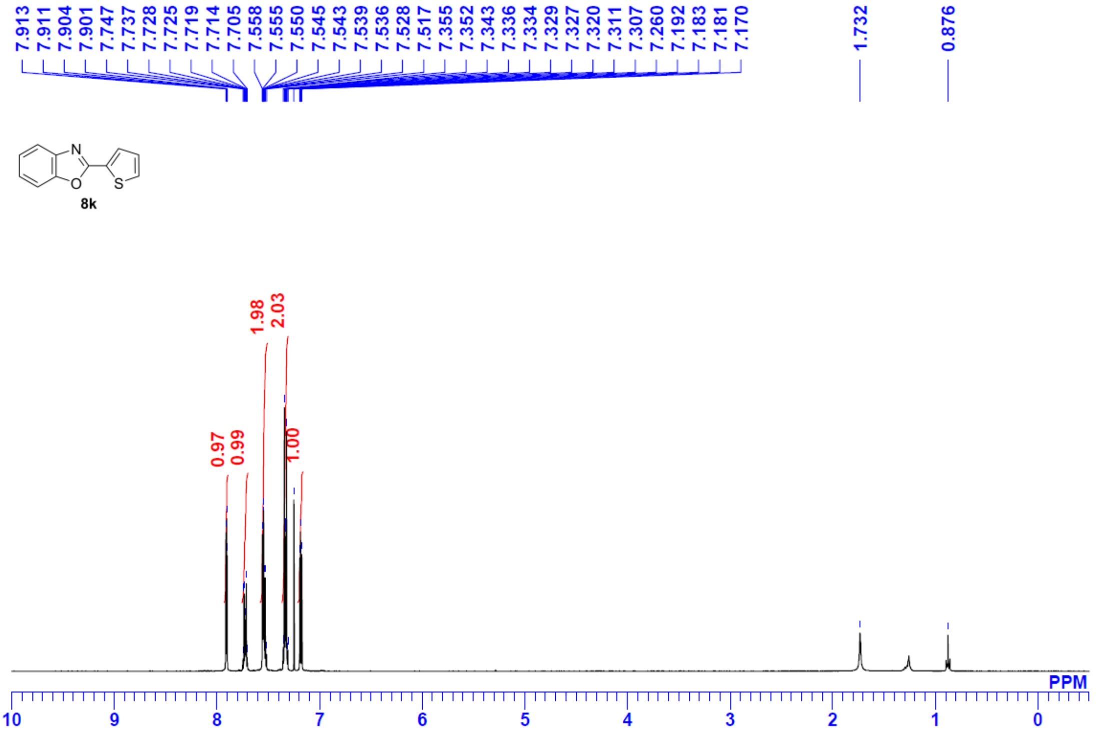

^{13}C NMR of **8k**

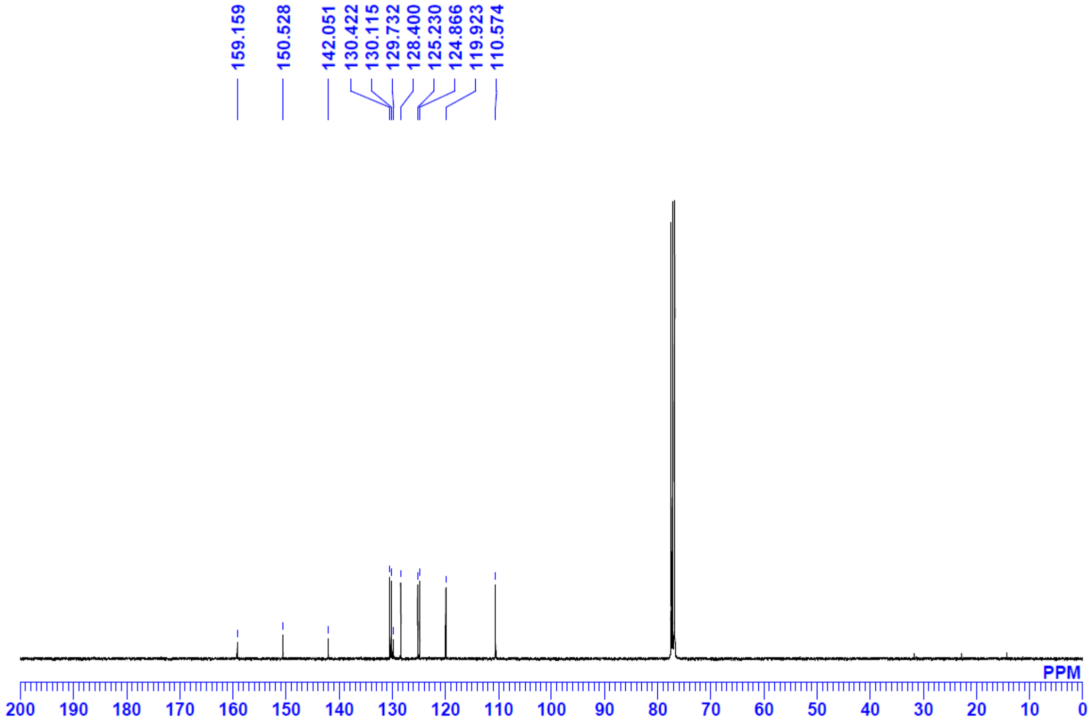

^{1}H NMR of **8l**

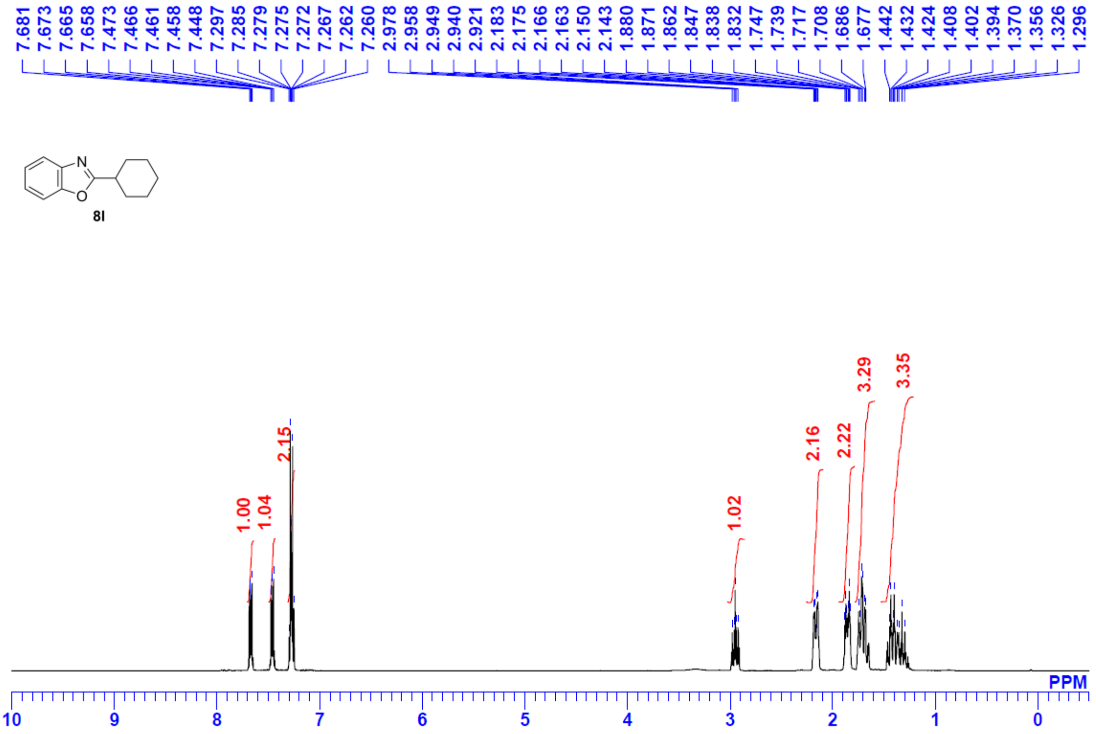

8l

^{13}C NMR of **8l**

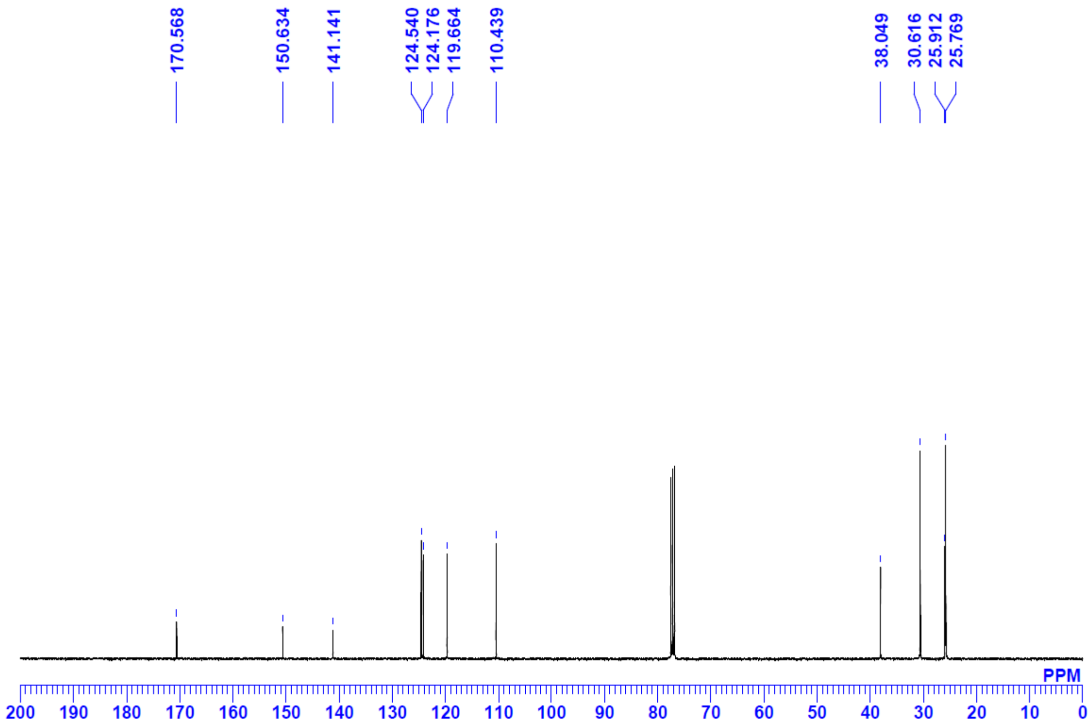

^{1}H NMR of **8m**

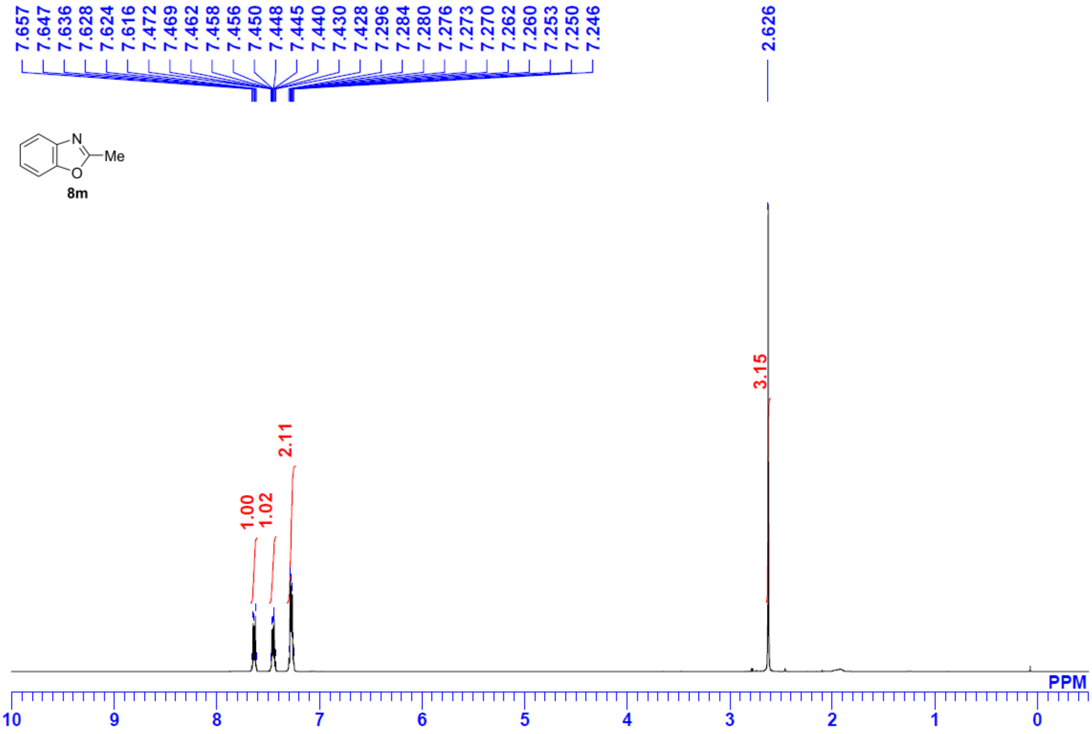

^{13}C NMR of **8m**

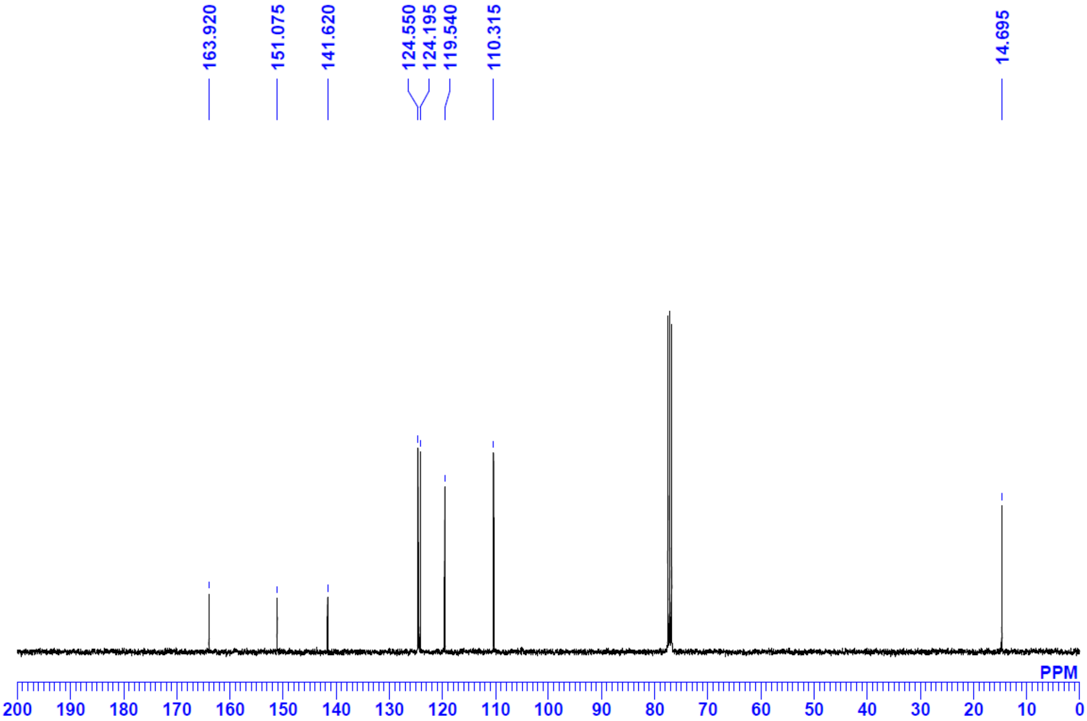

^{1}H NMR of **8n**

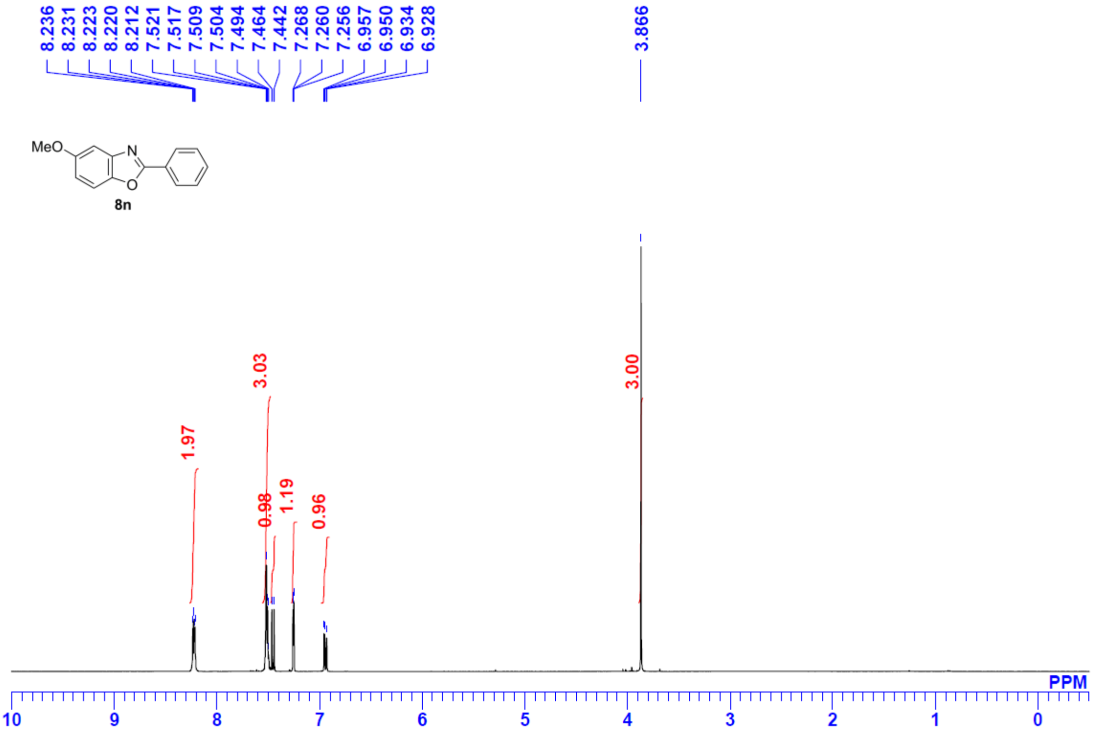

^{13}C NMR of **8n**

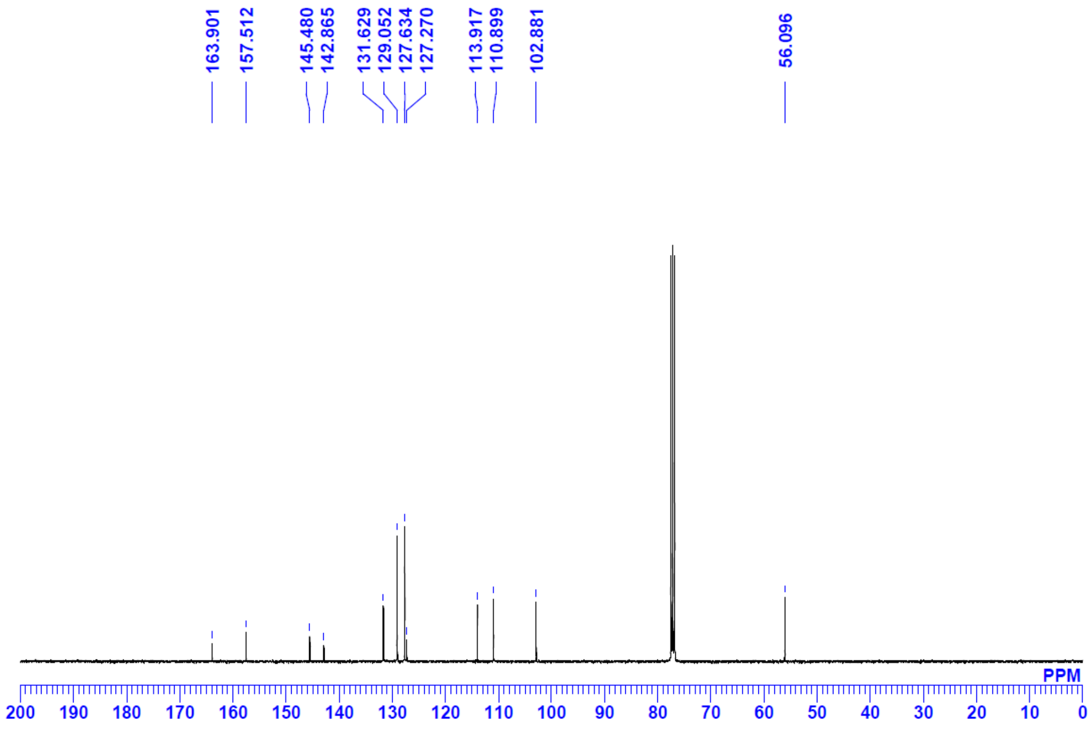

^{1}H NMR of **8o**

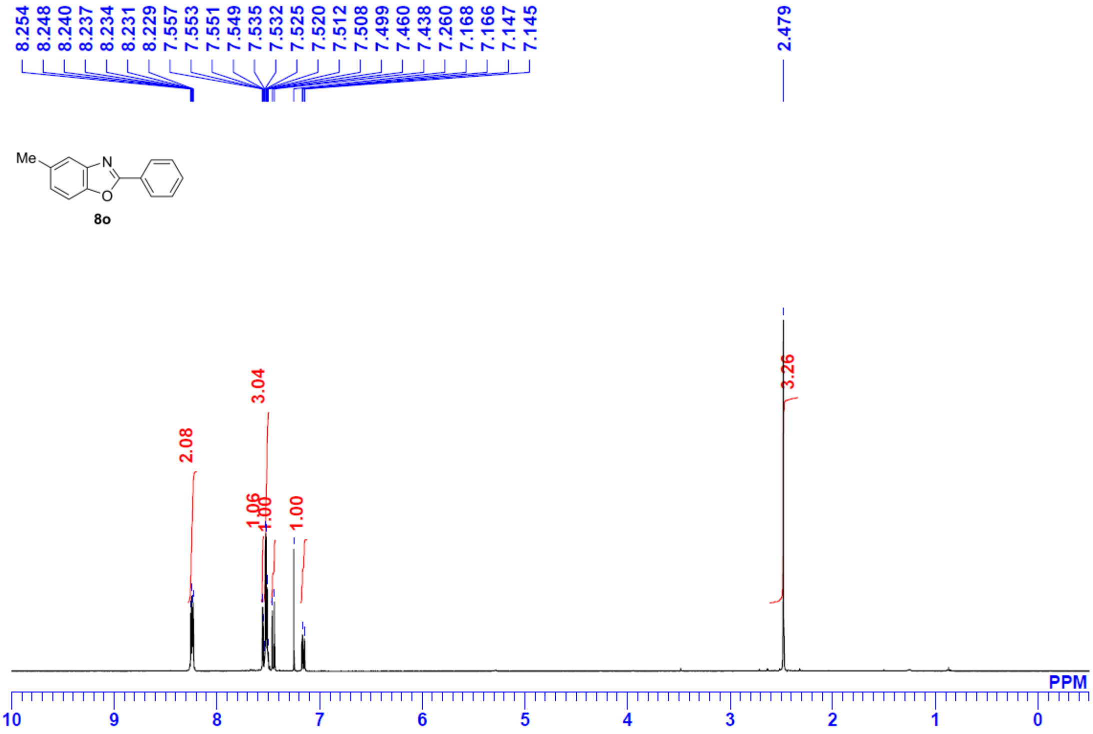

^{13}C NMR of **8o**

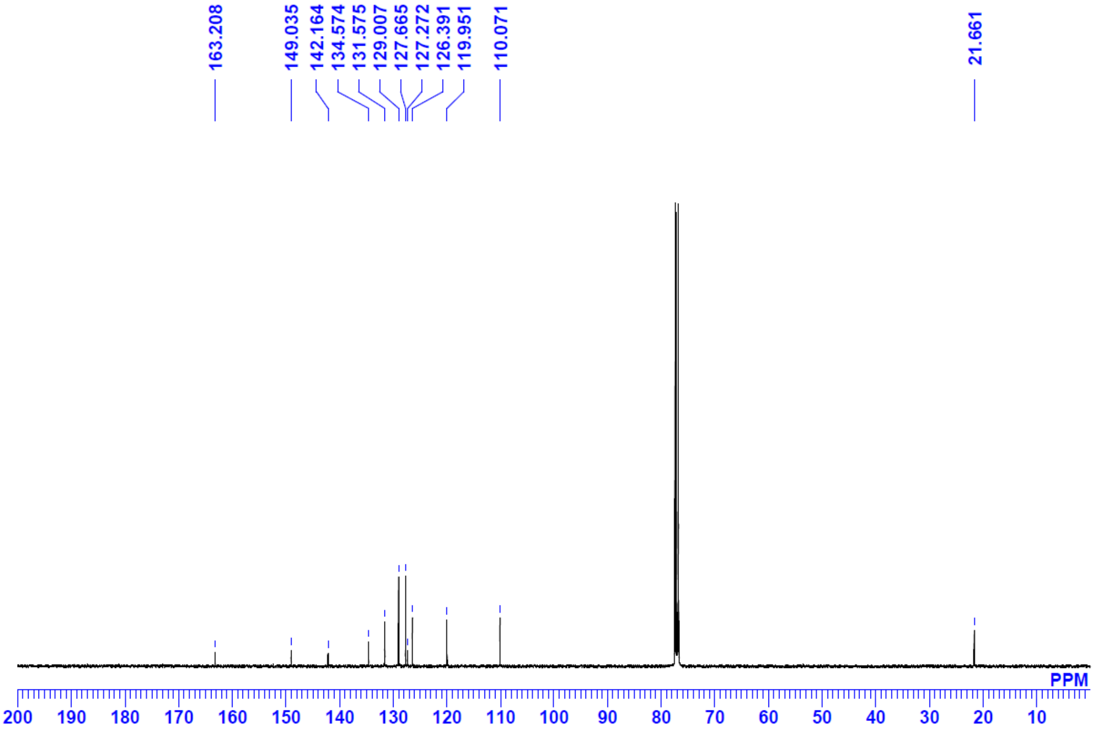

^{1}H NMR of **8p**

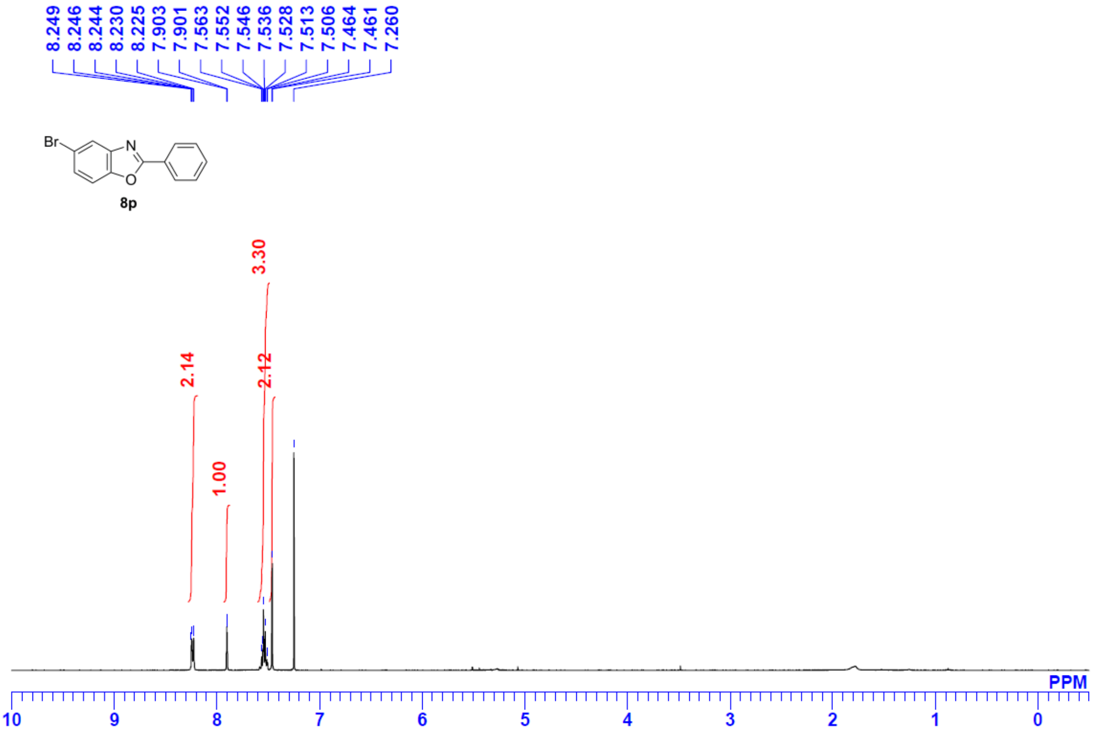

^{13}C NMR of **8p**

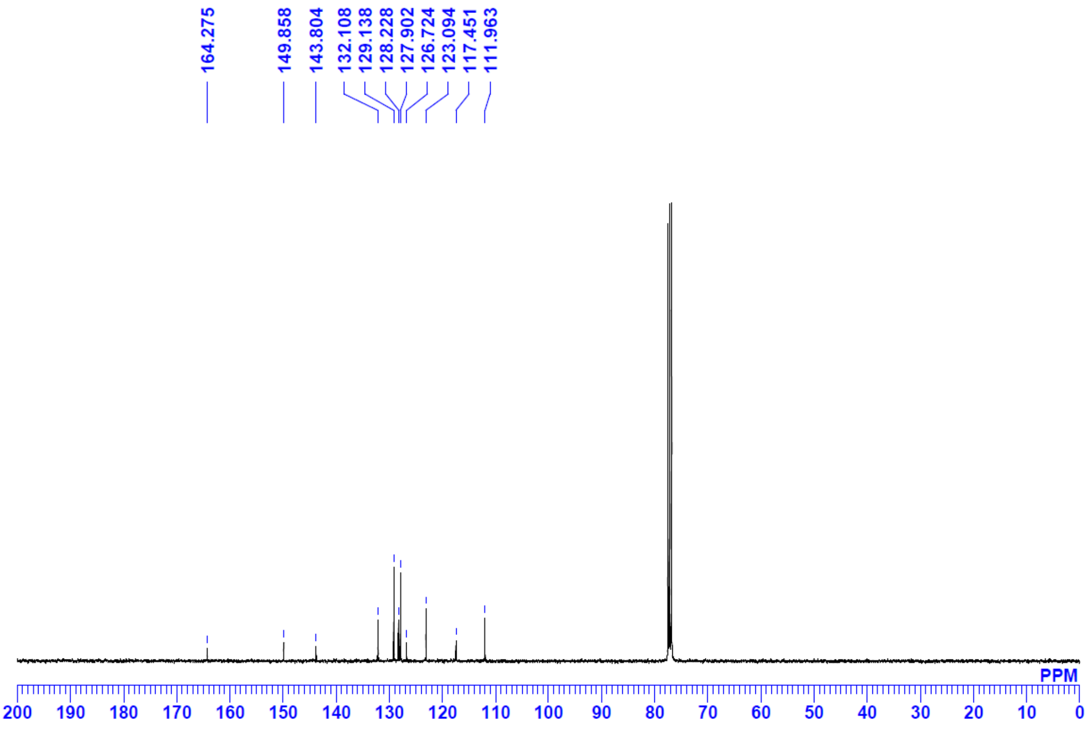

^{1}H NMR of **8q**

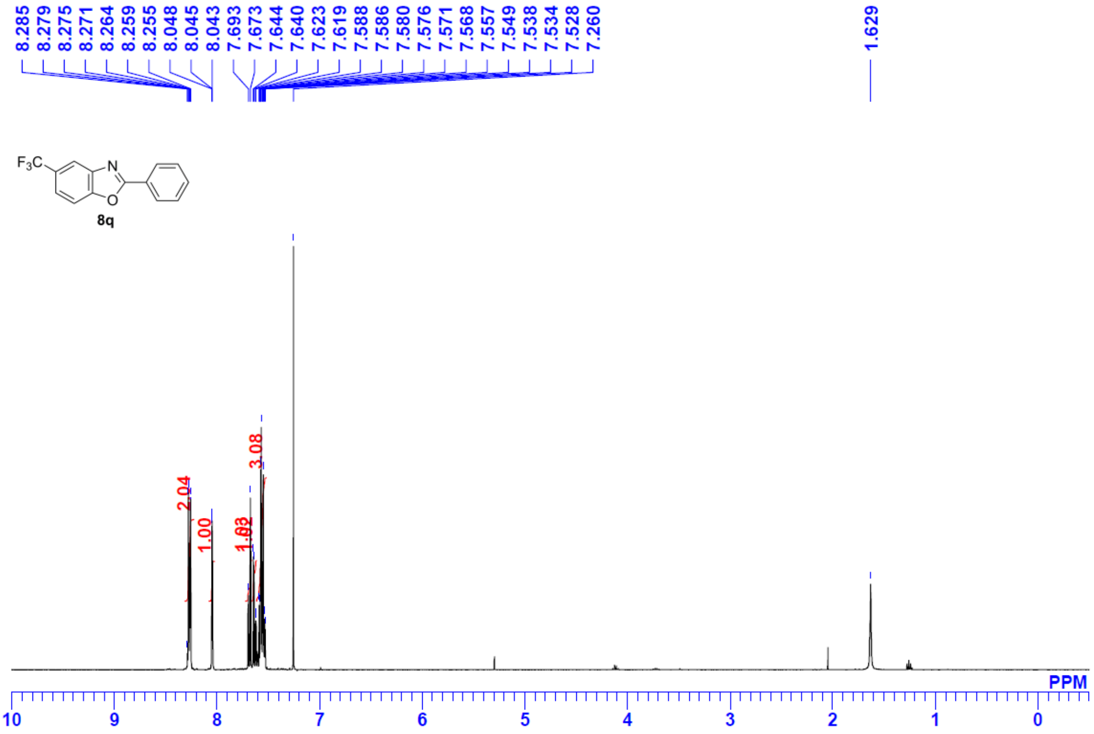

^{13}C NMR of **8q**

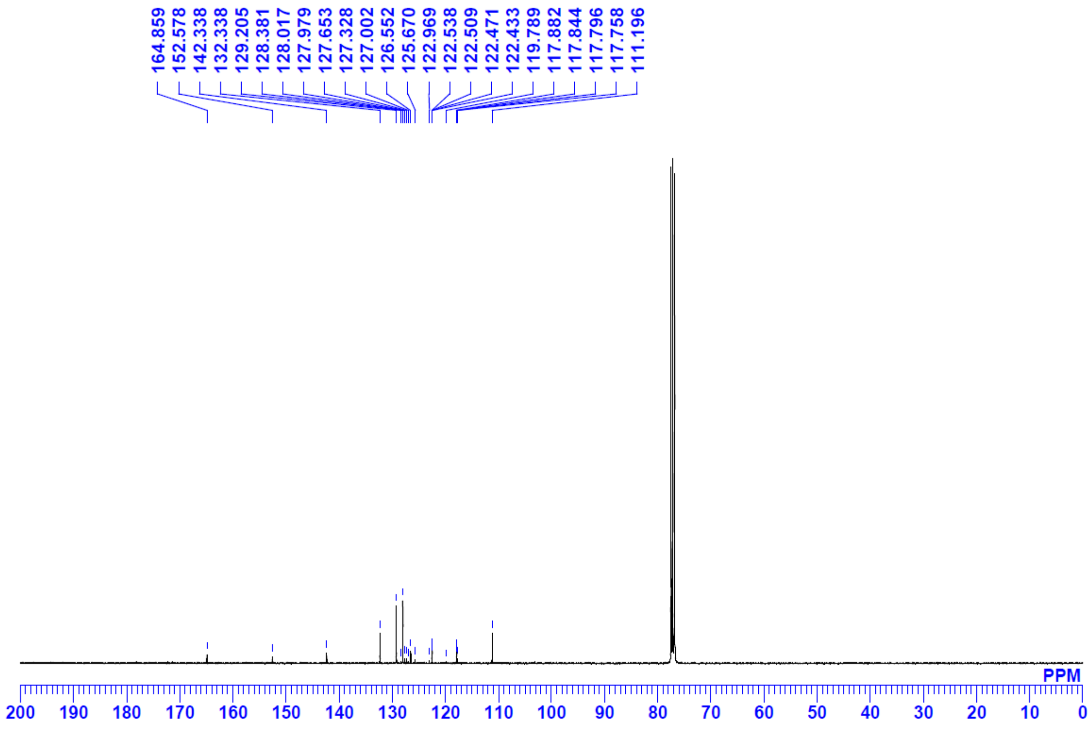

^{19}F NMR of **8q**

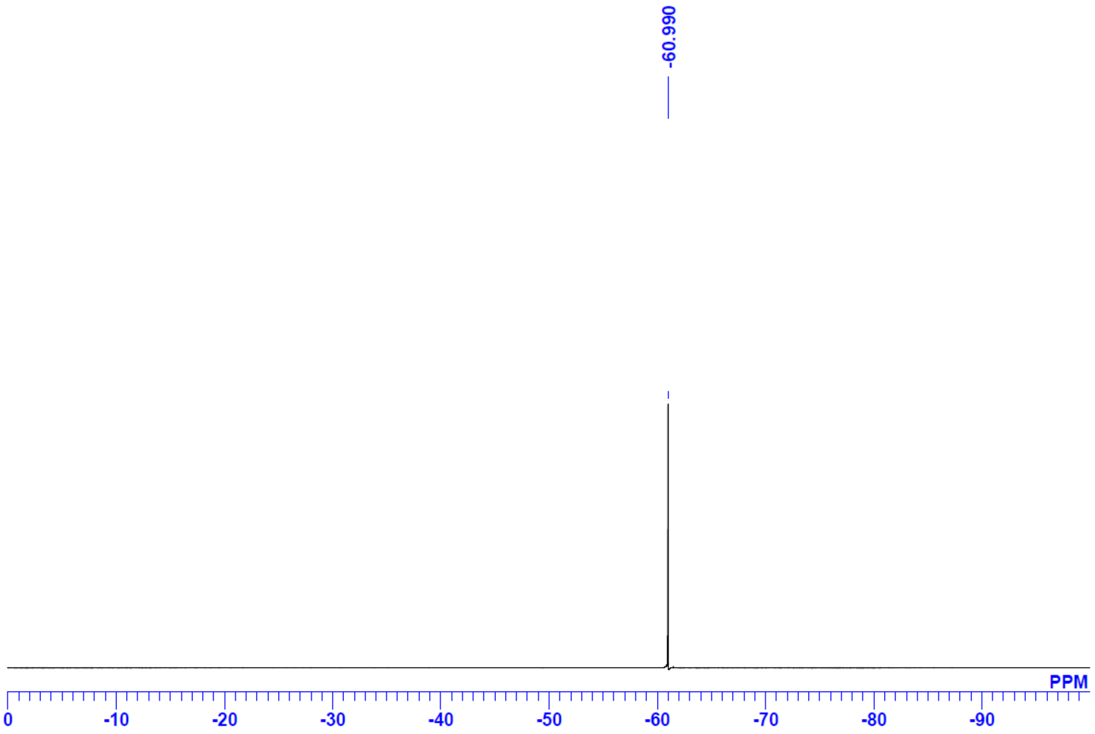

^{1}H NMR of **8r**

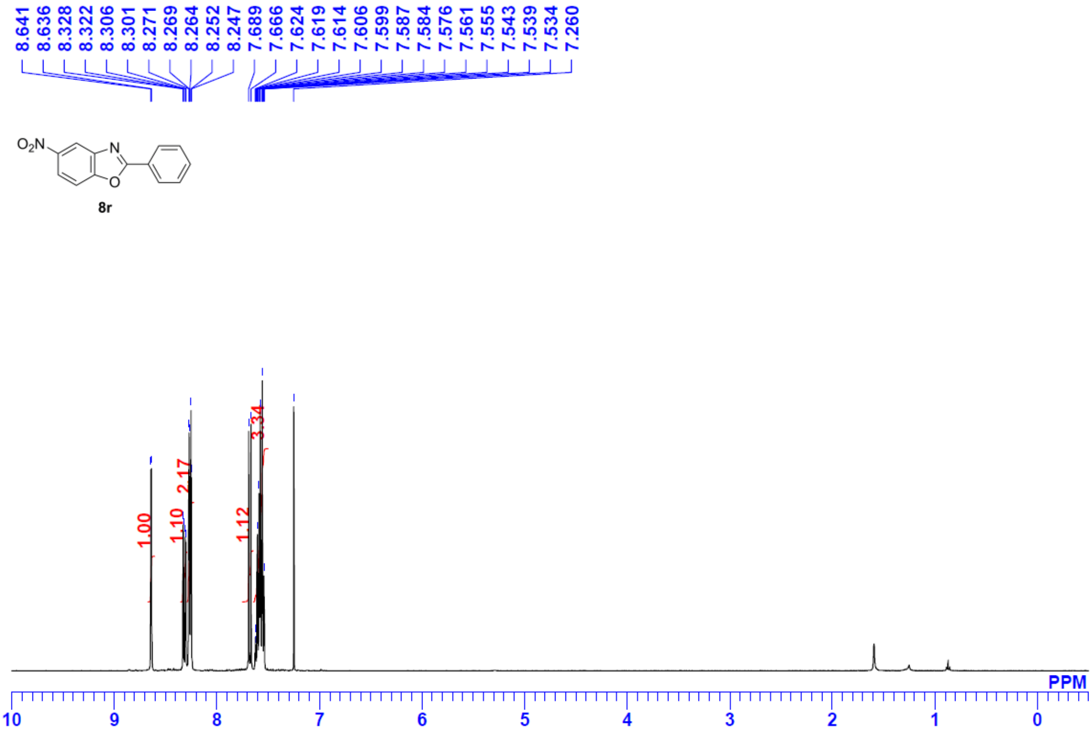

^{13}C NMR of **8r**

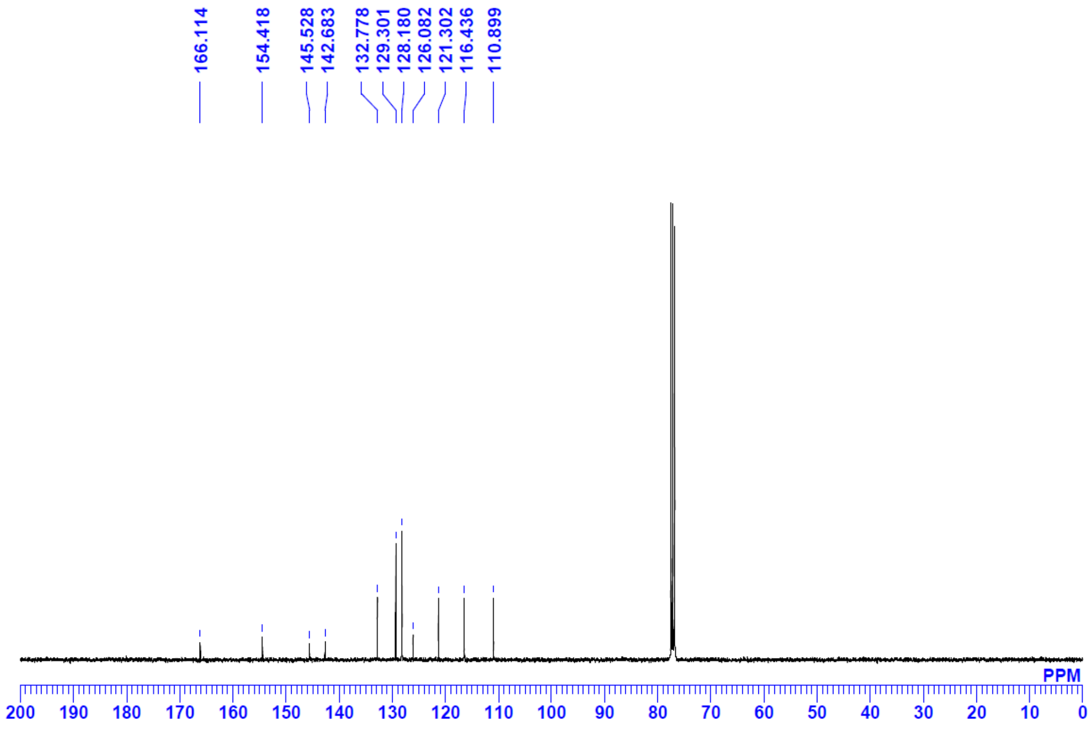

^{1}H NMR of **8s**

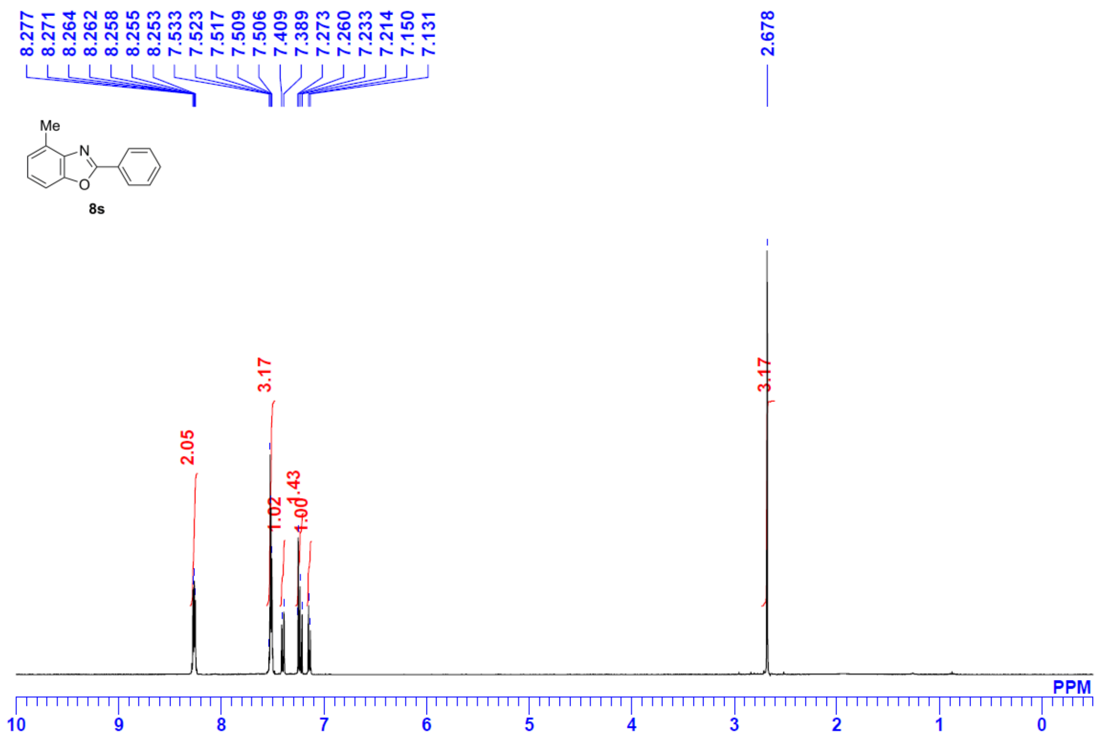

^{13}C NMR of **8s**

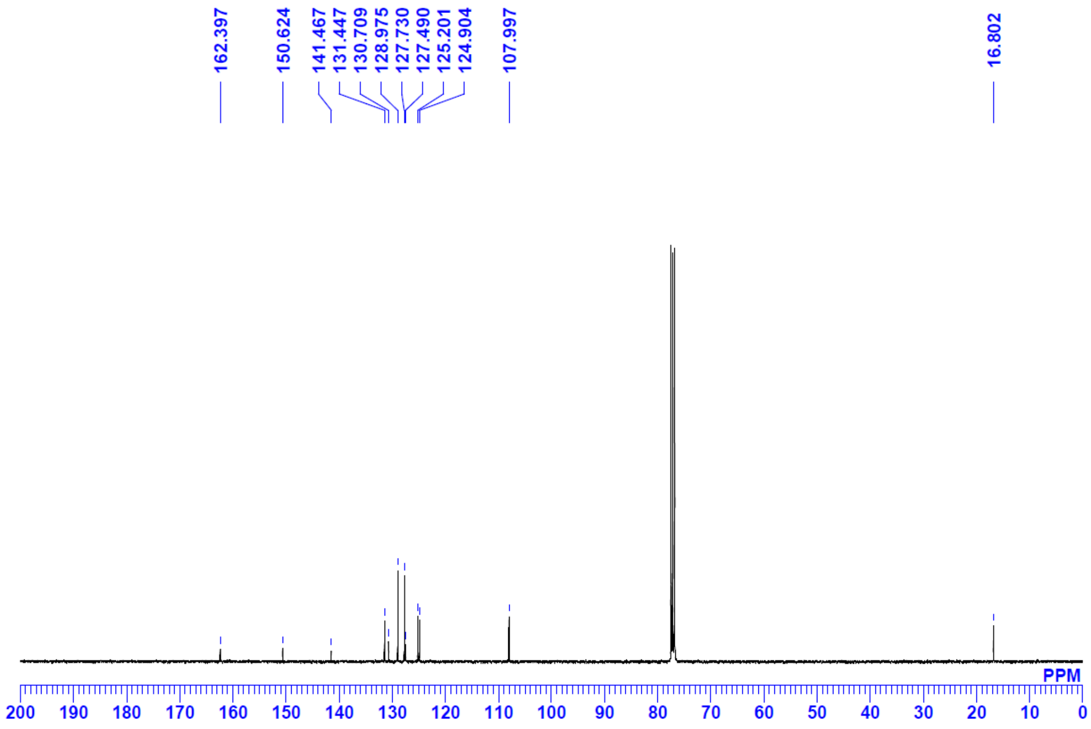

^{1}H NMR of **8t**

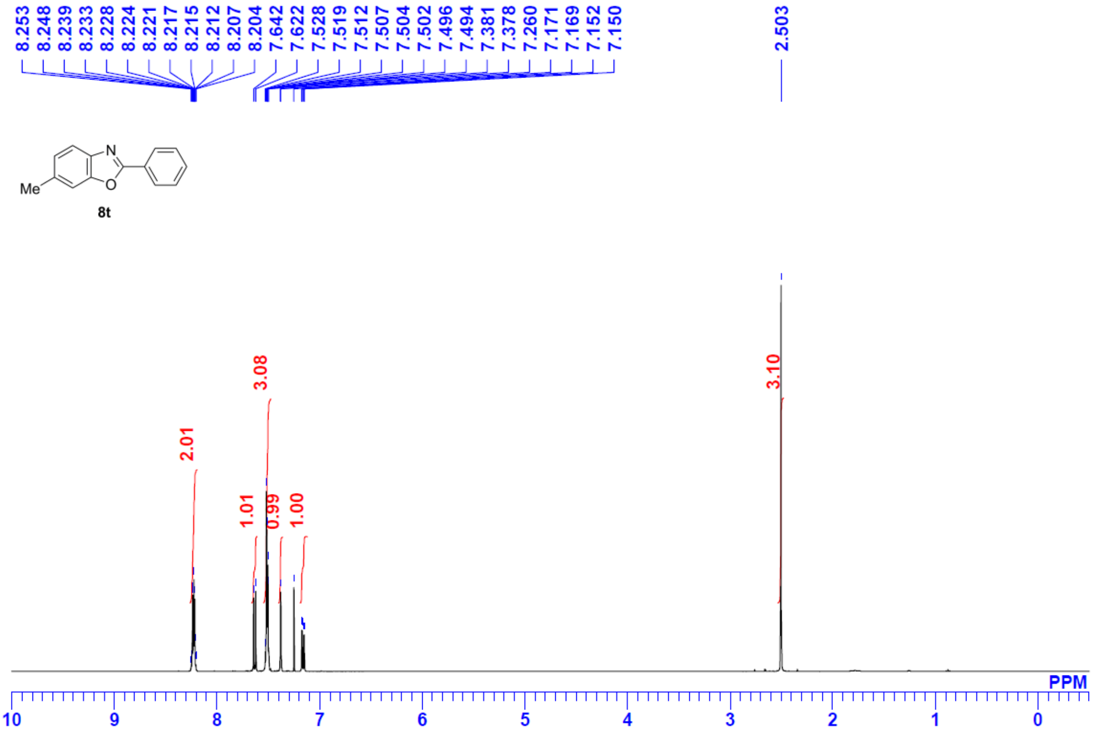

^{13}C NMR of **8t**

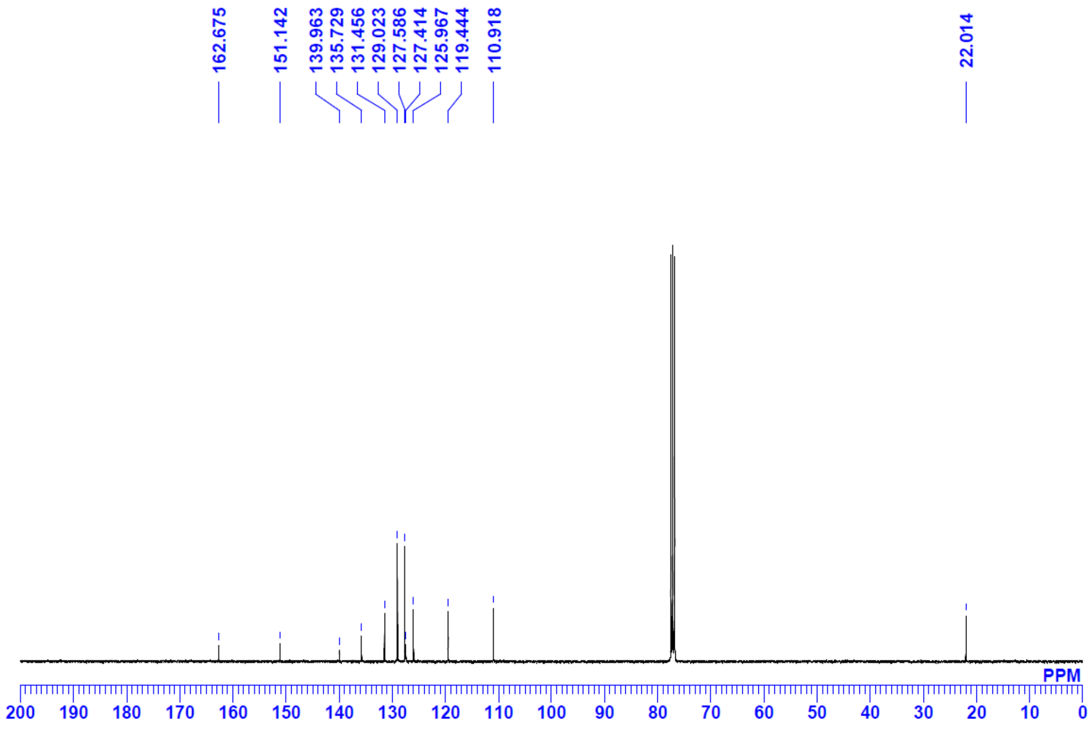

^{1}H NMR of **8u**

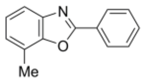

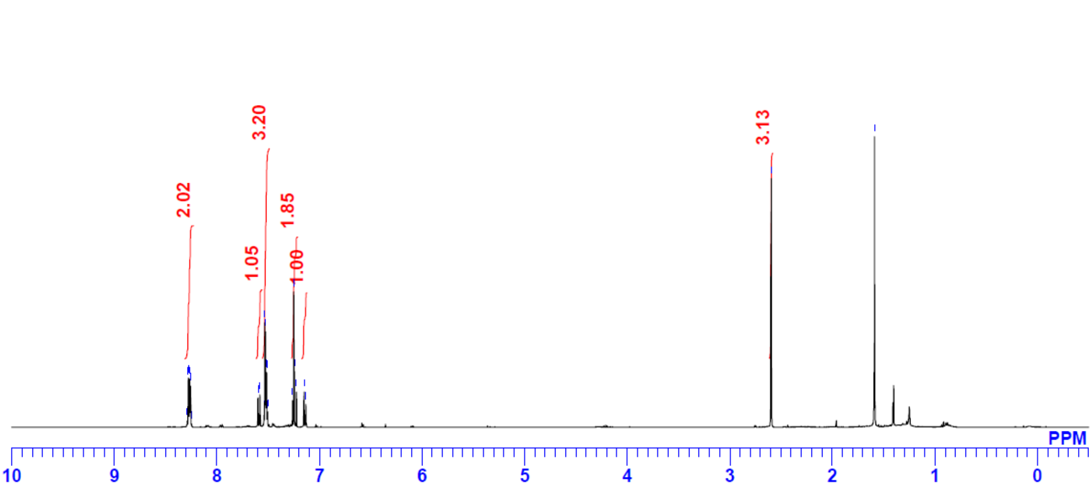

^{13}C NMR of **8u**

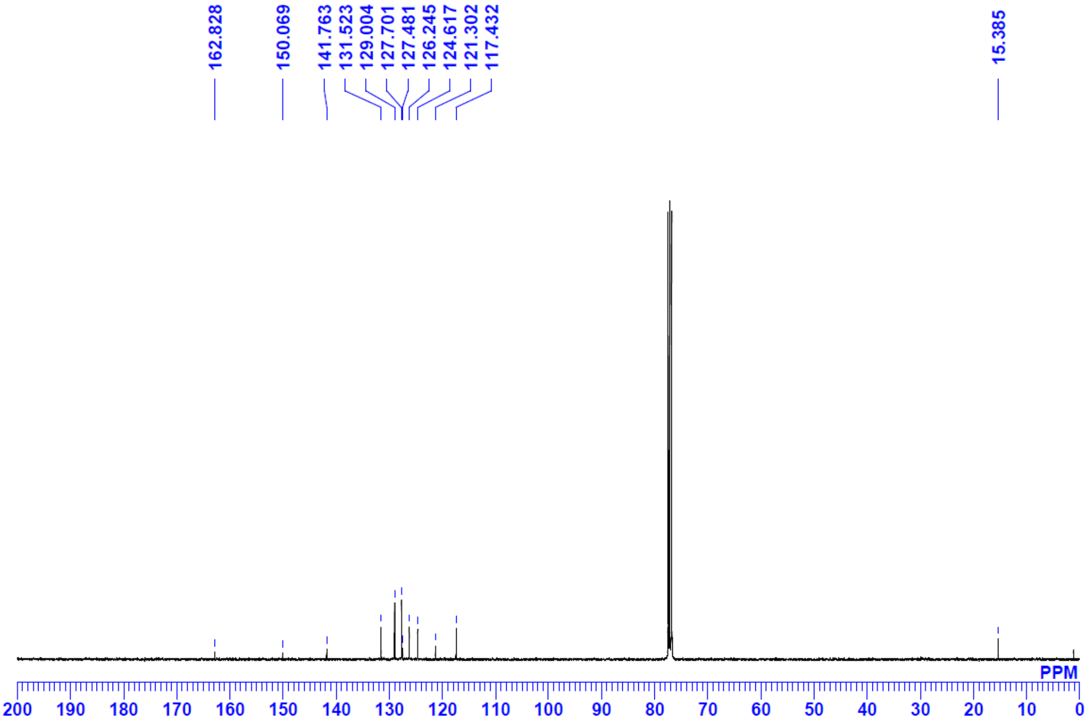

^{1}H NMR of **8v**

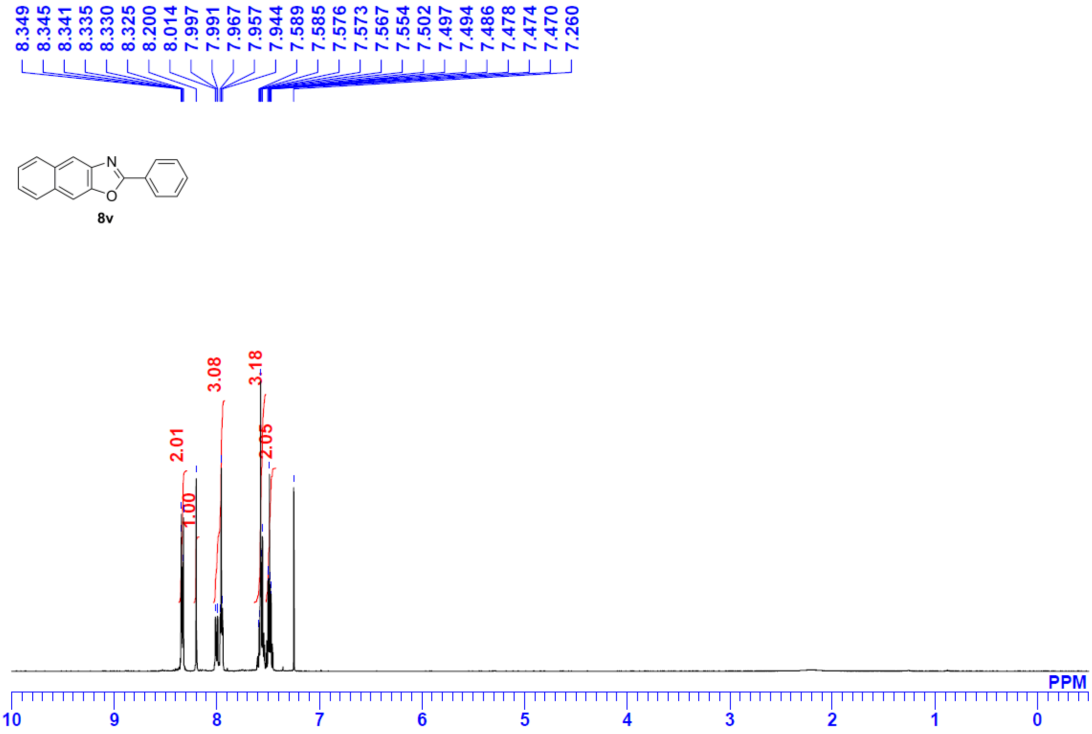

^{13}C NMR of **8v**

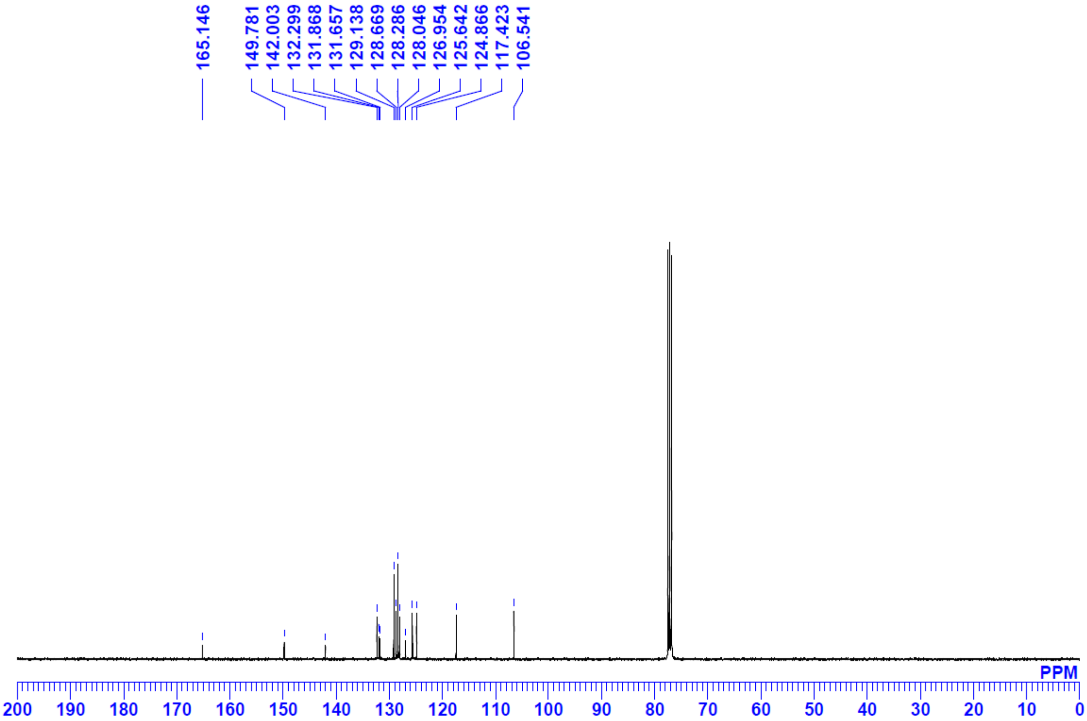

^{1}H NMR of **8w**

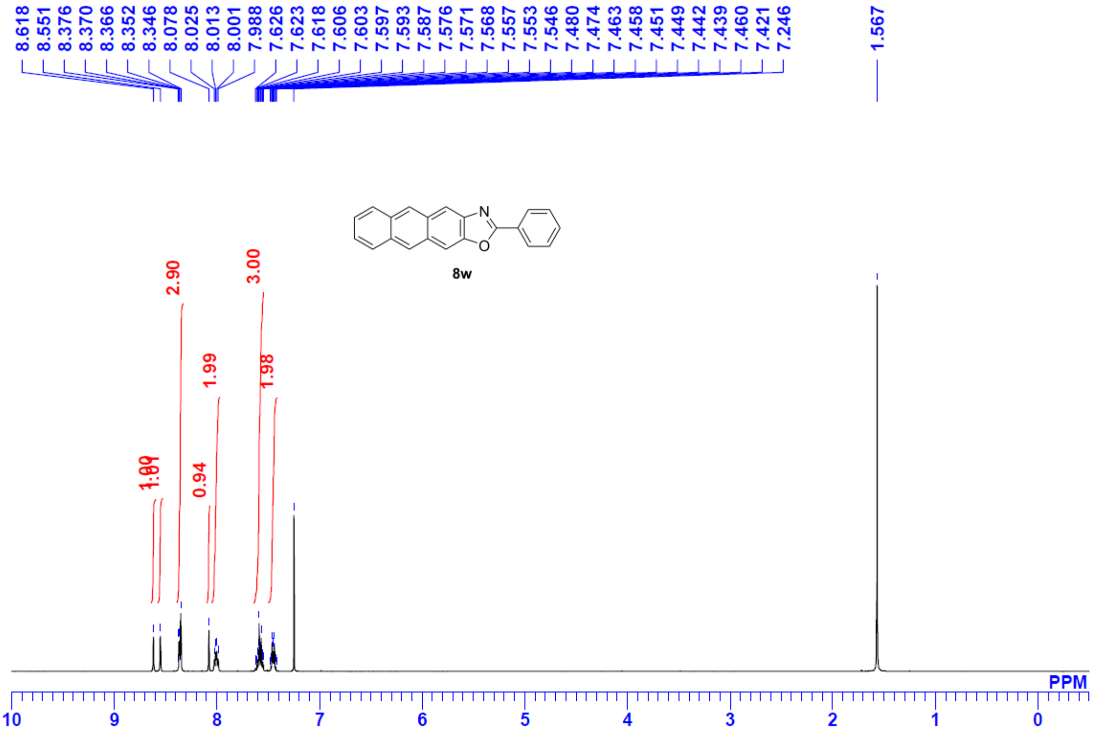

^{13}C NMR of **8w**

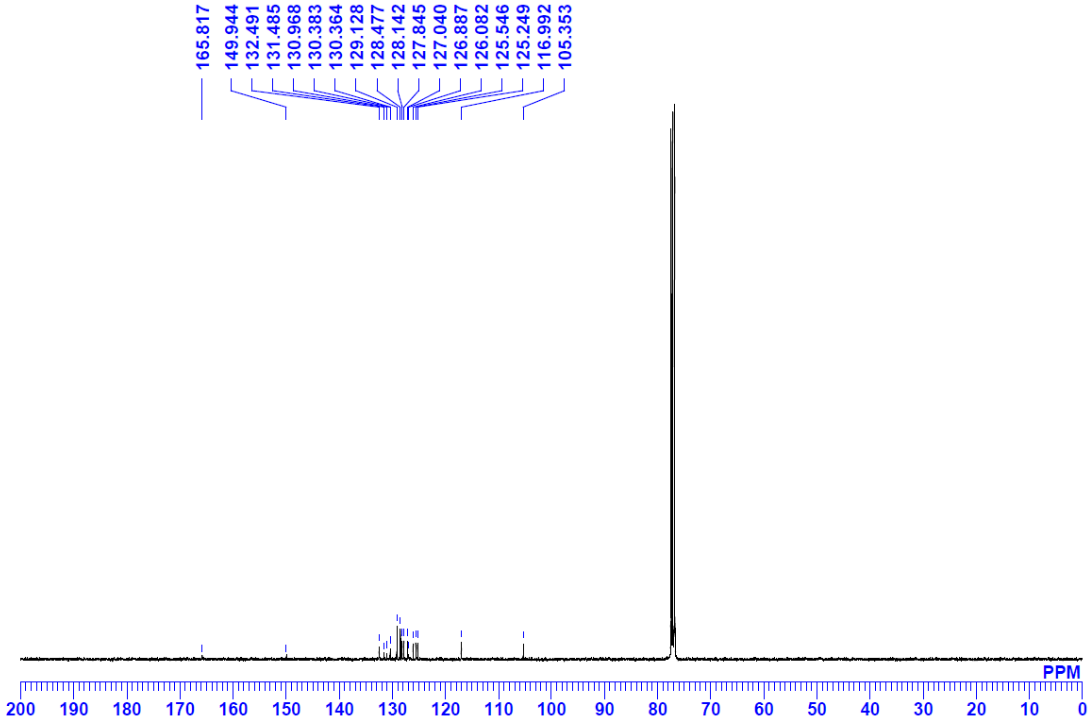

^{1}H NMR of **9**

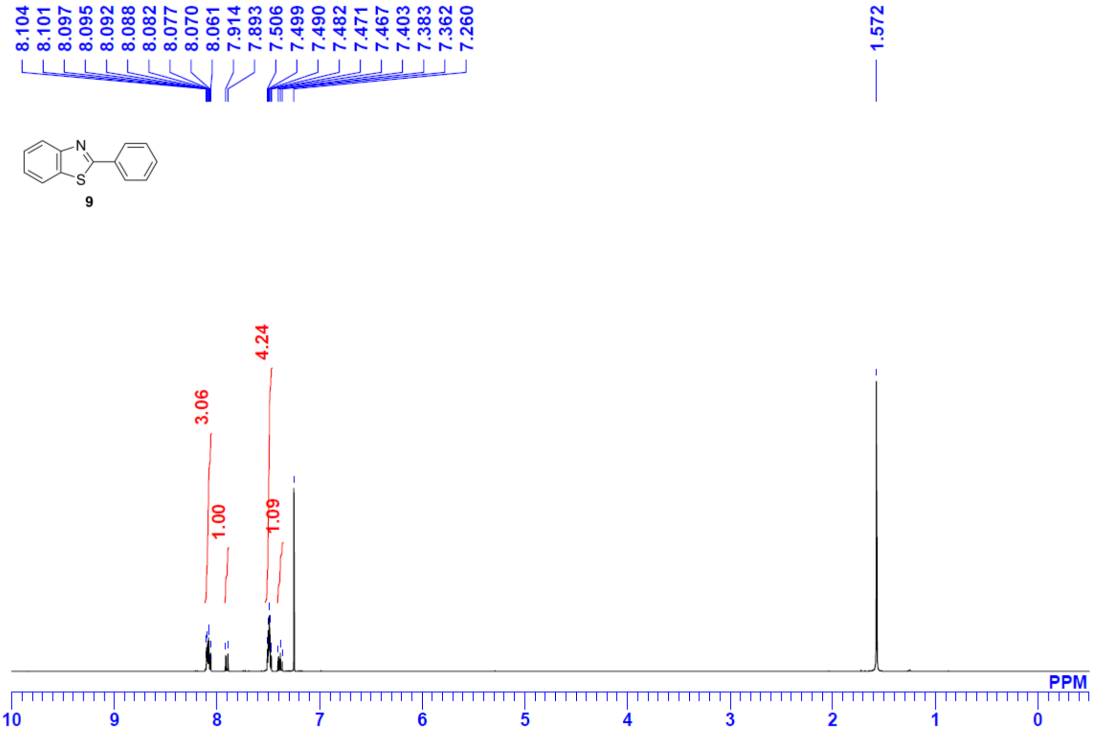

^{13}C NMR of **9**

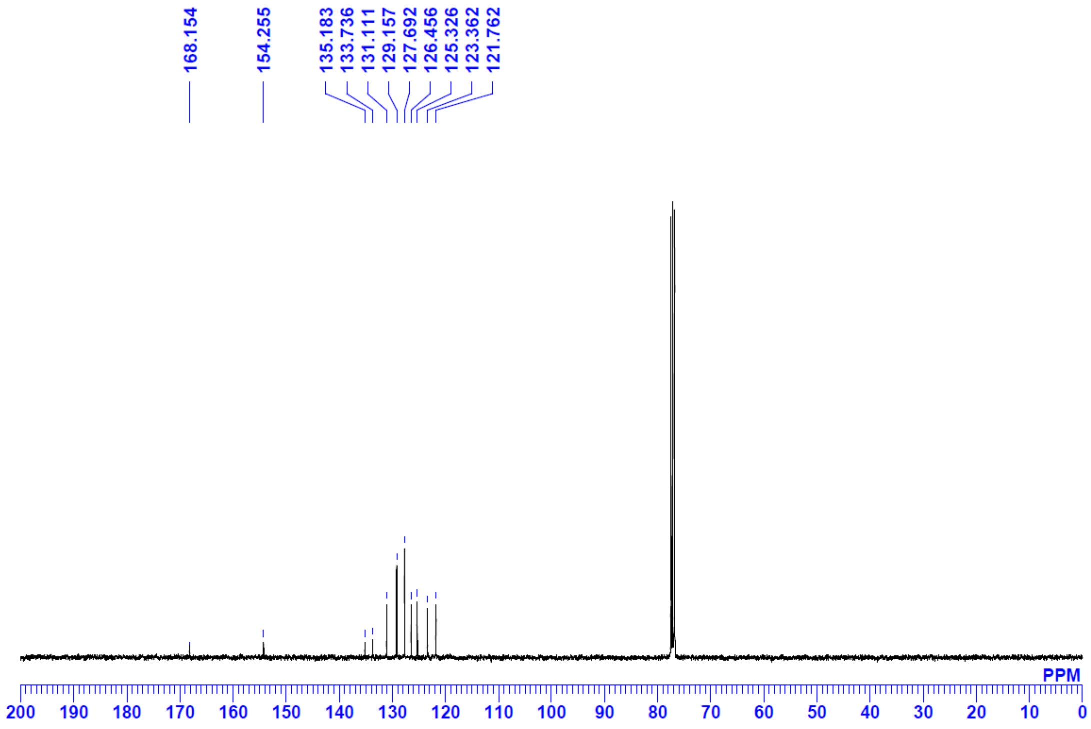

^{1}H NMR of **12**

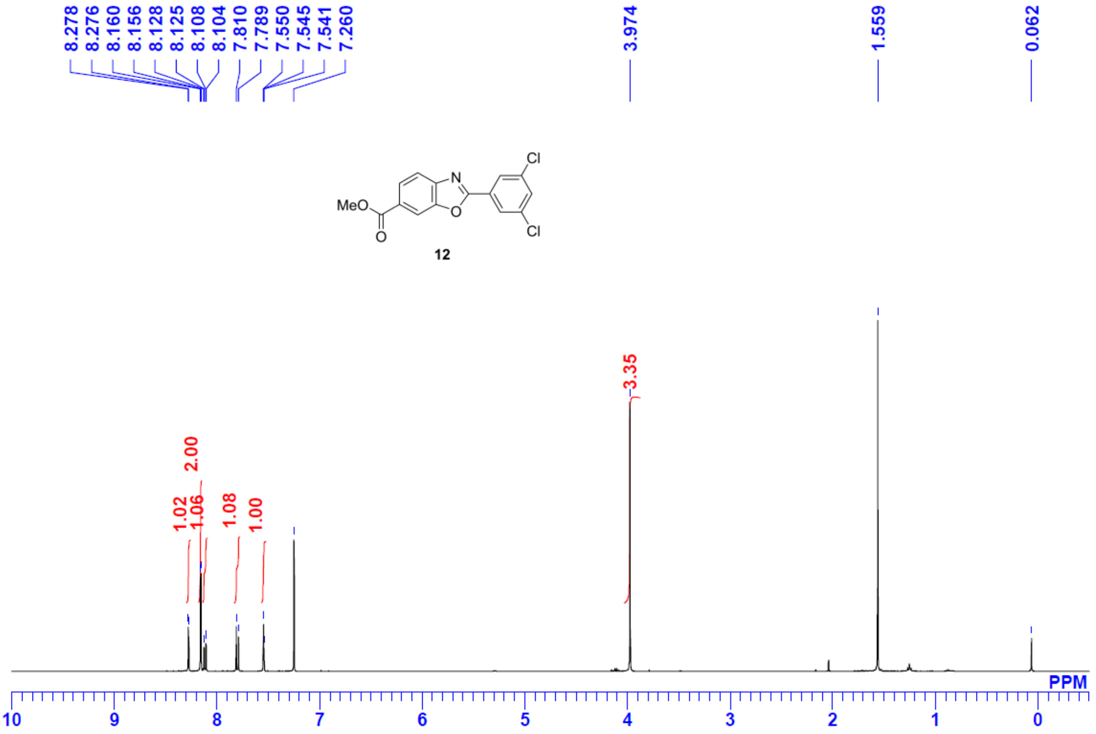

^{13}C NMR of **12**

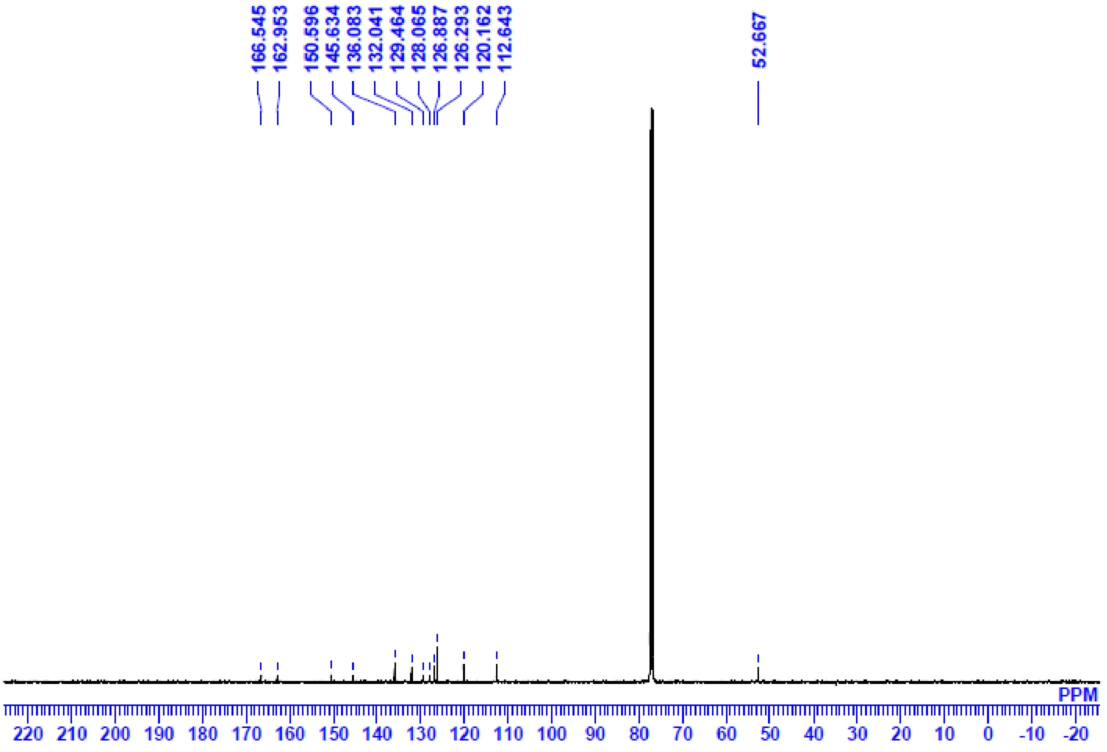

^{1}H NMR of **13 (Tafamidis)**

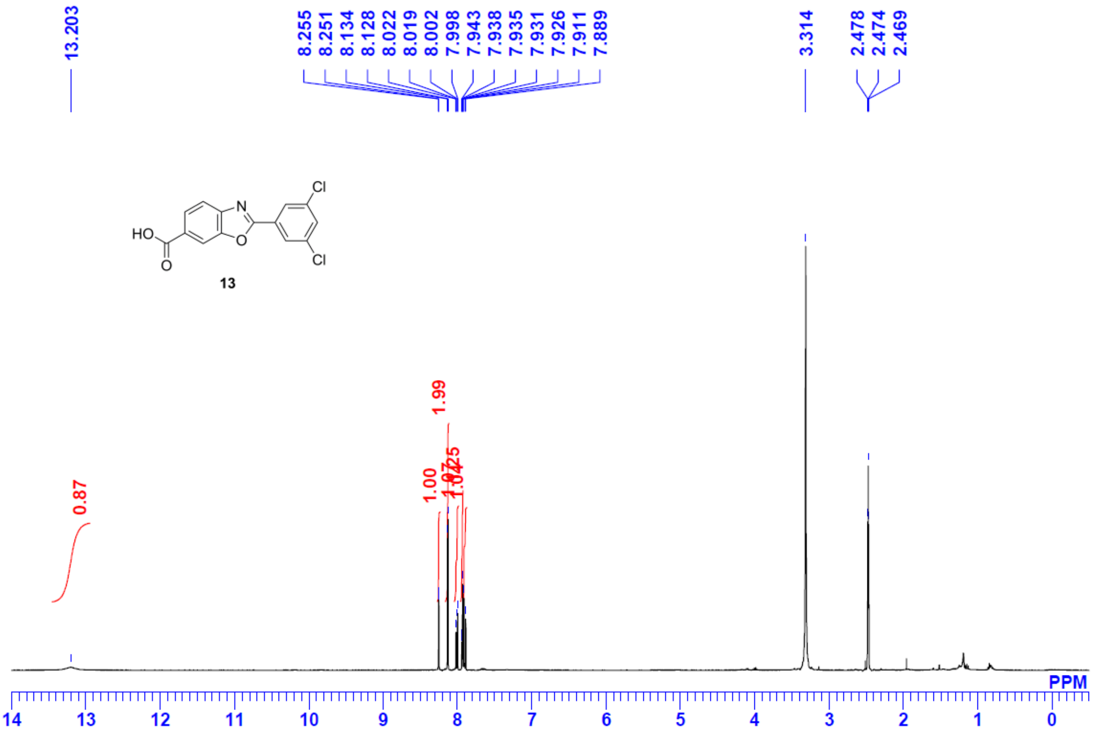

^{13}C NMR of **13 (Tafamidis)**

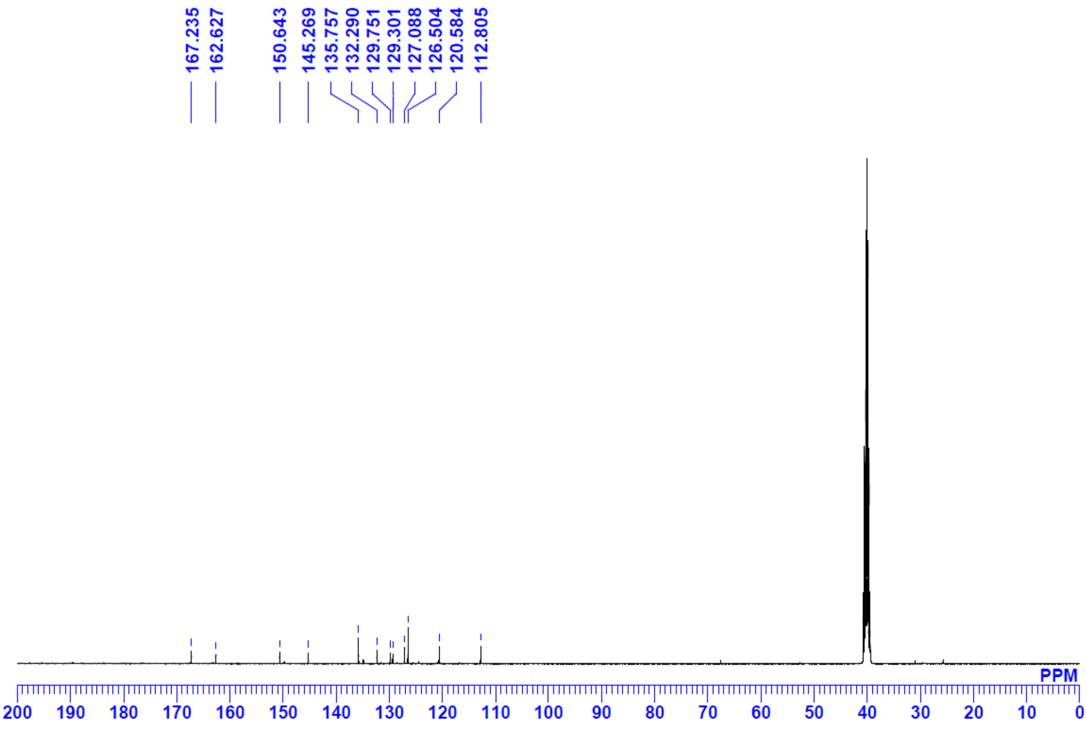

^{1}H NMR of **16b**

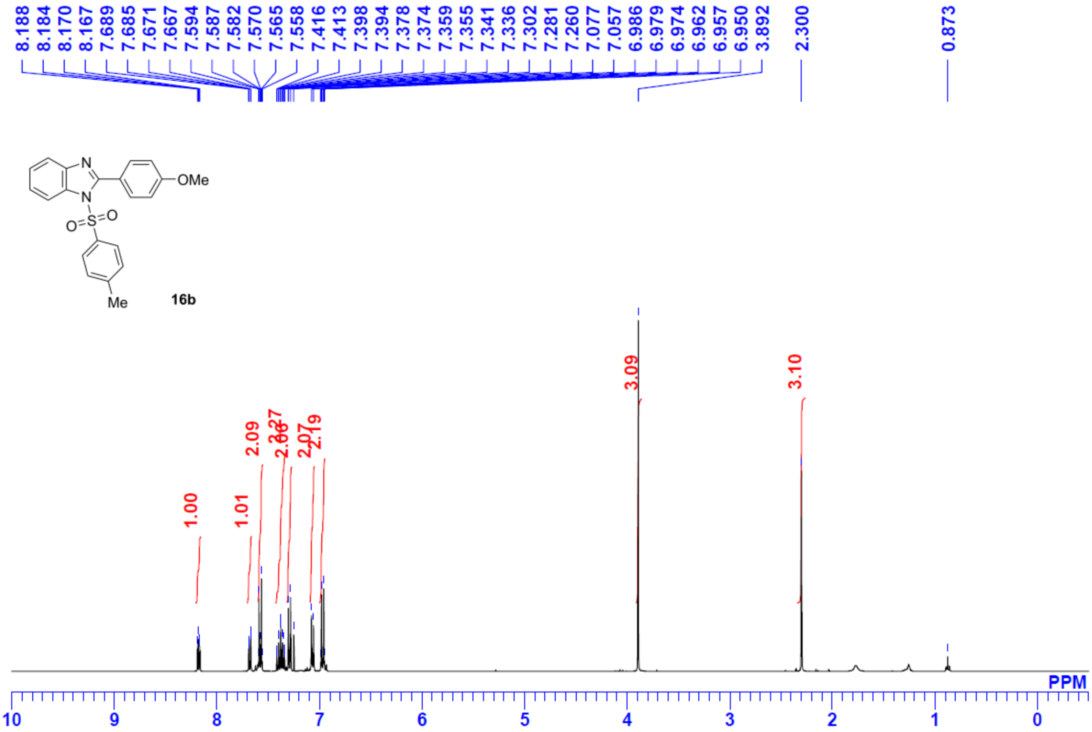

^{13}C NMR of **16b**

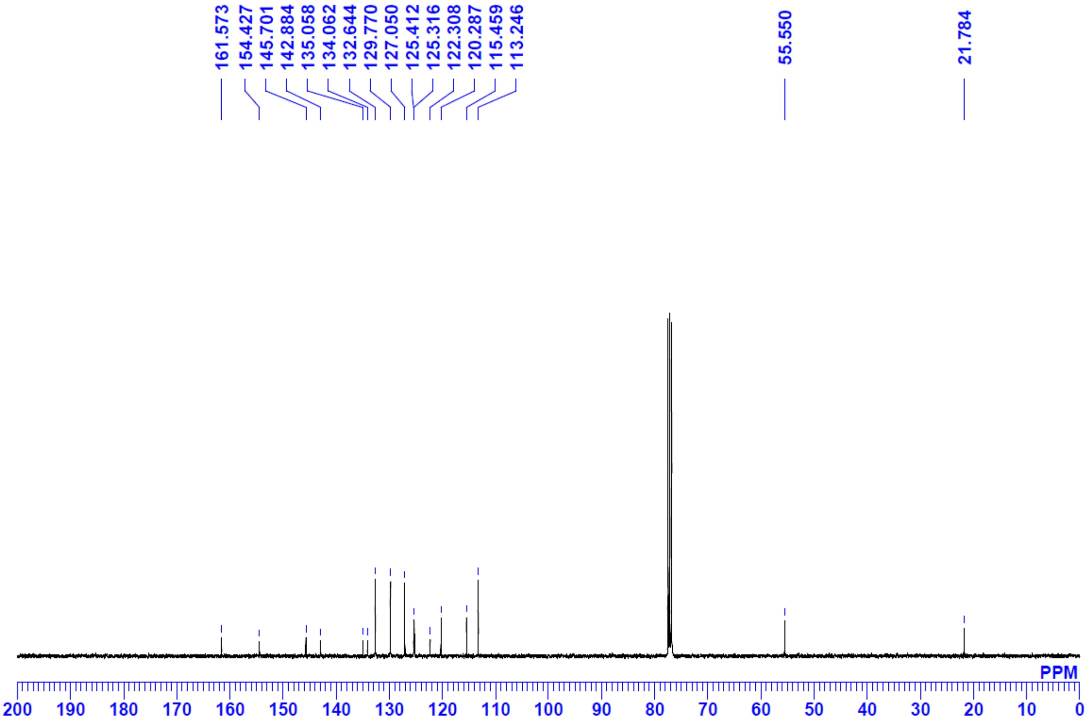

^{1}H NMR of **16c**

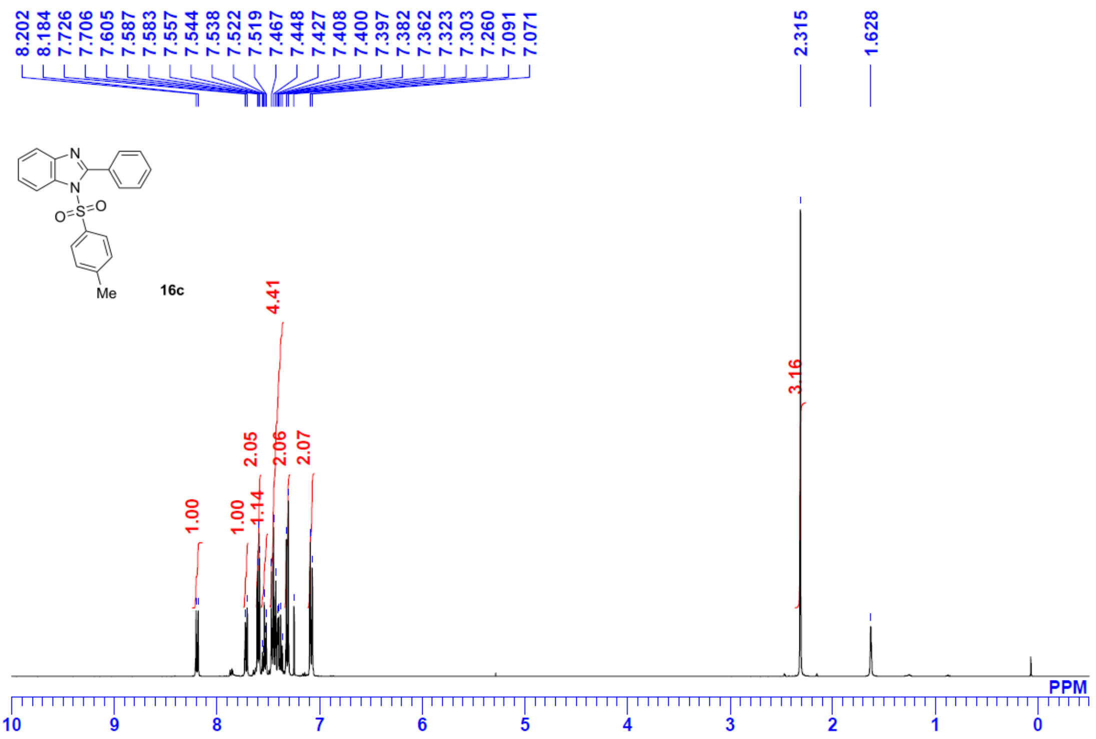

^{13}C NMR of **16c**

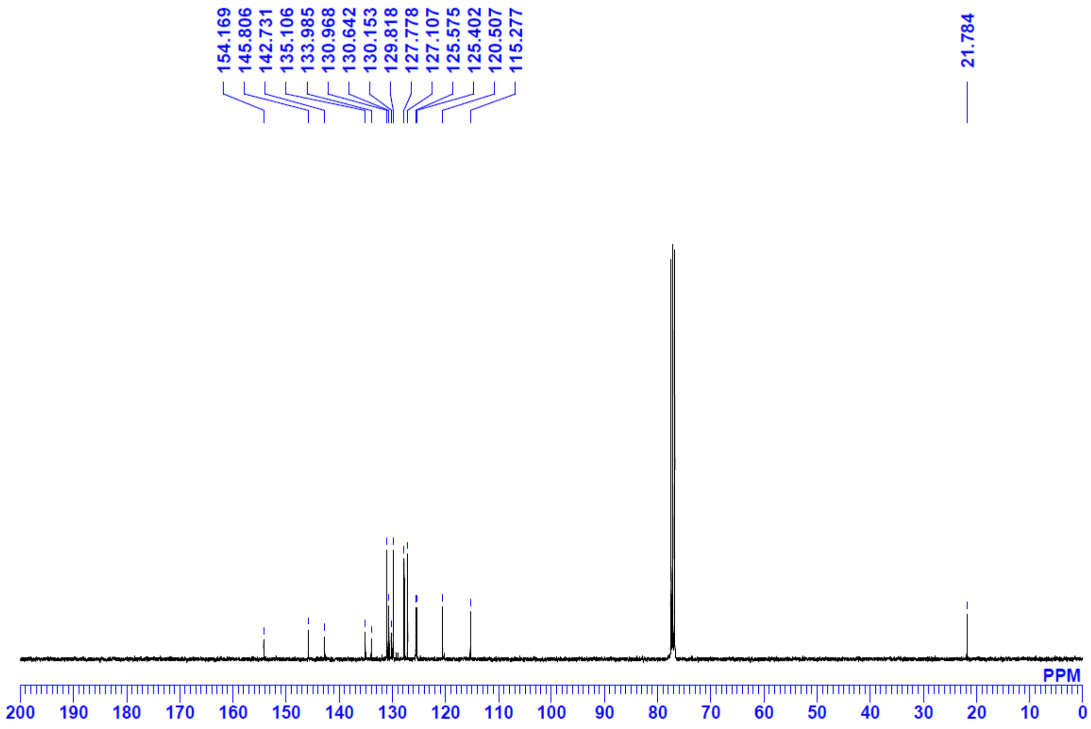

^{1}H NMR of **16d**

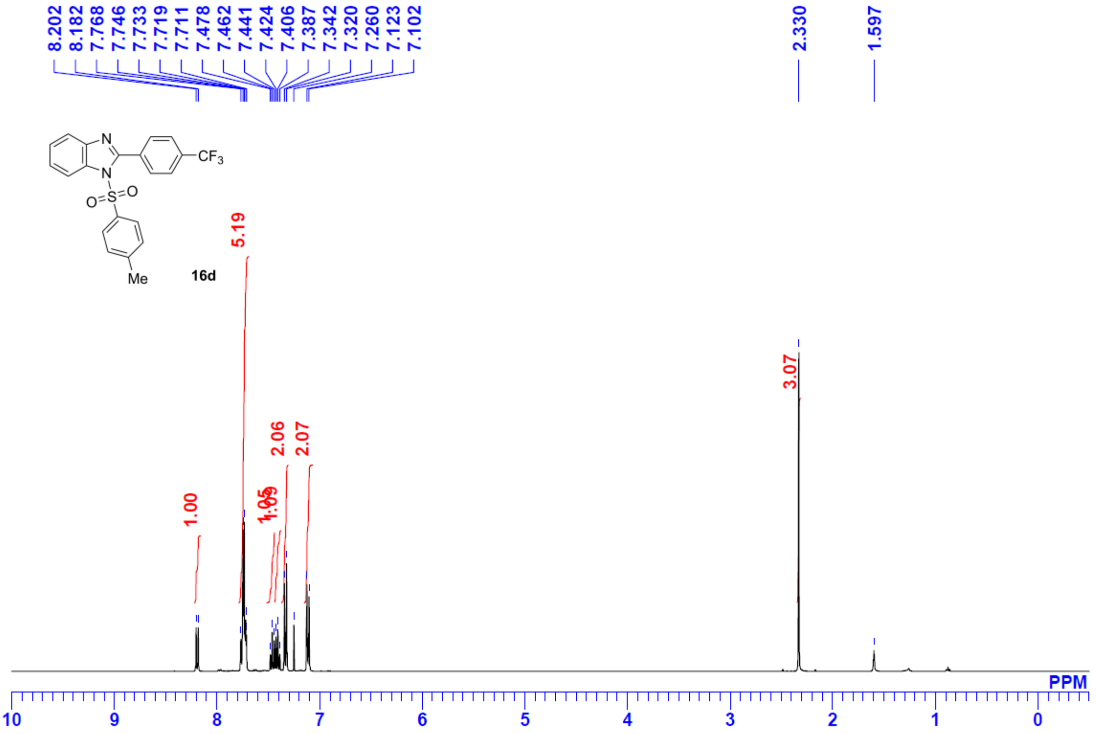

^{13}C NMR of **16d**

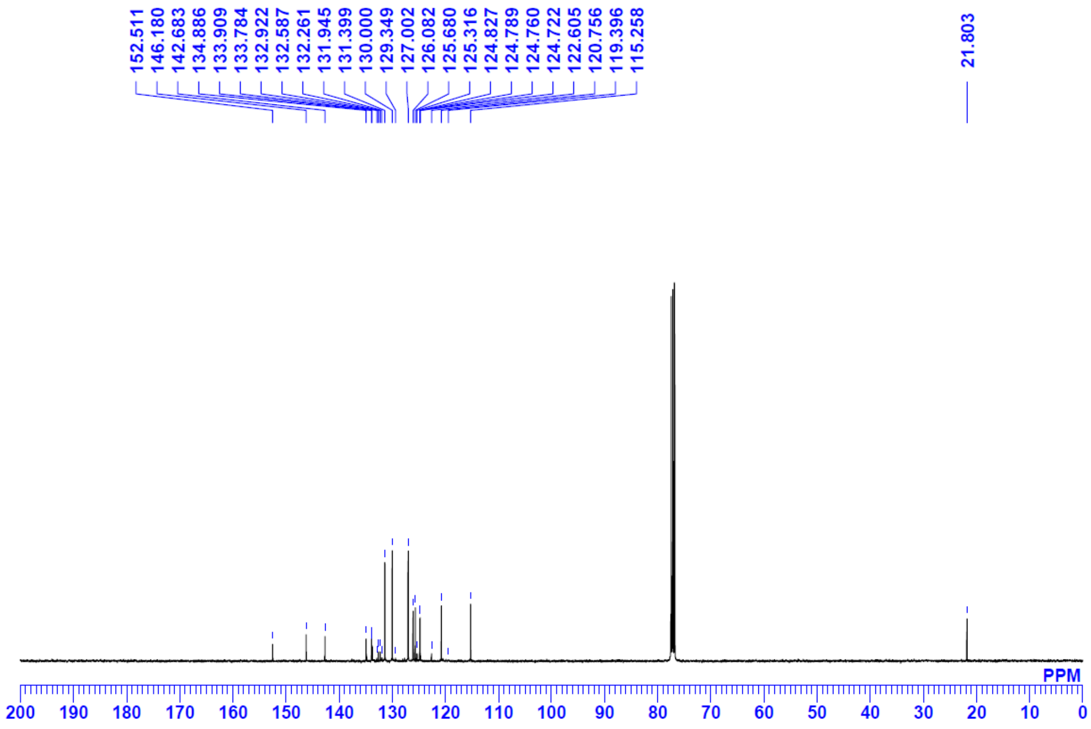

^{19}F NMR of **16d**

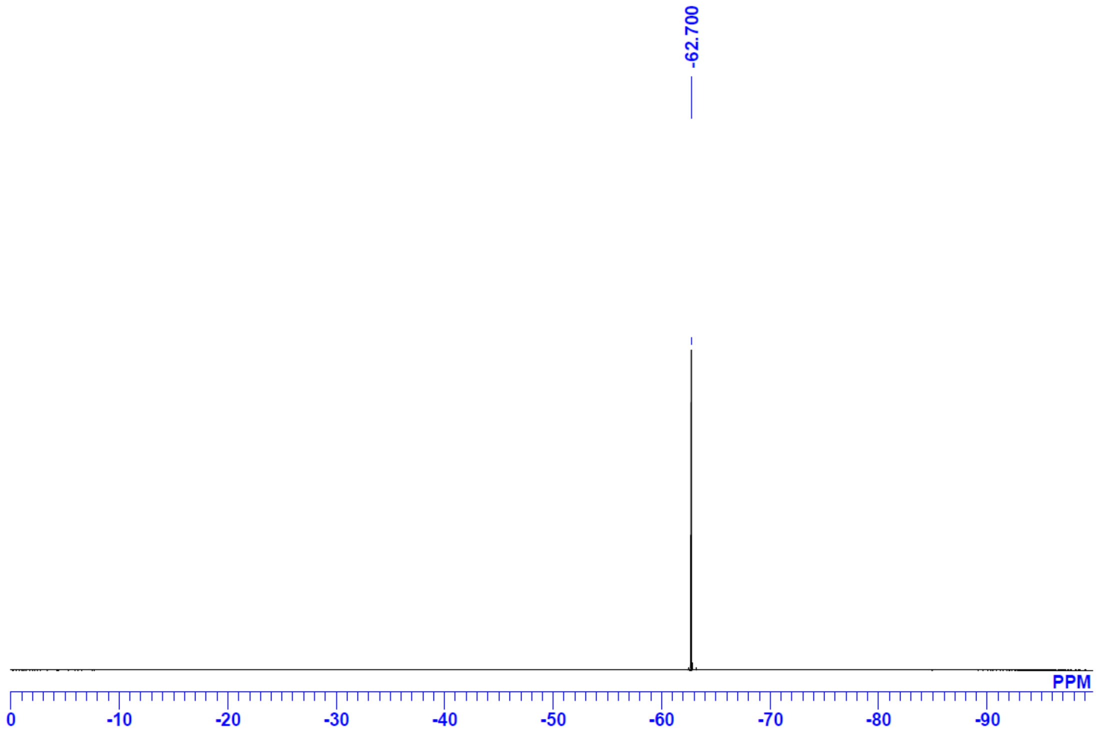

^{1}H NMR of **16e**

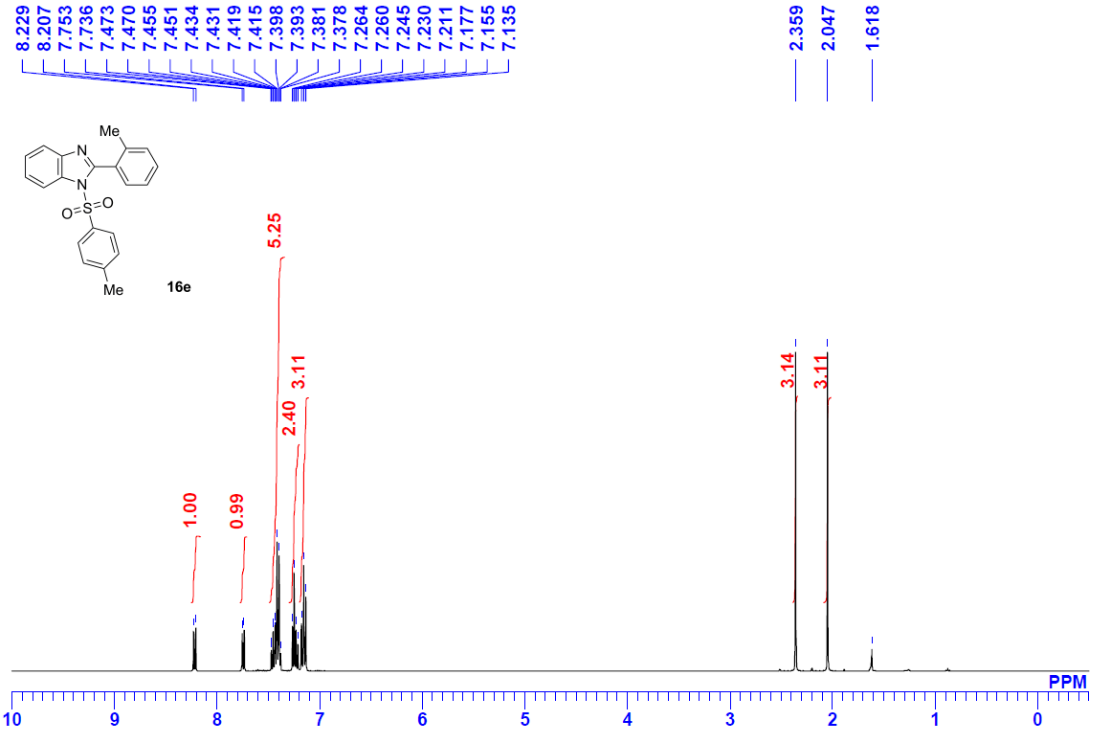

^{13}C NMR of **16e**

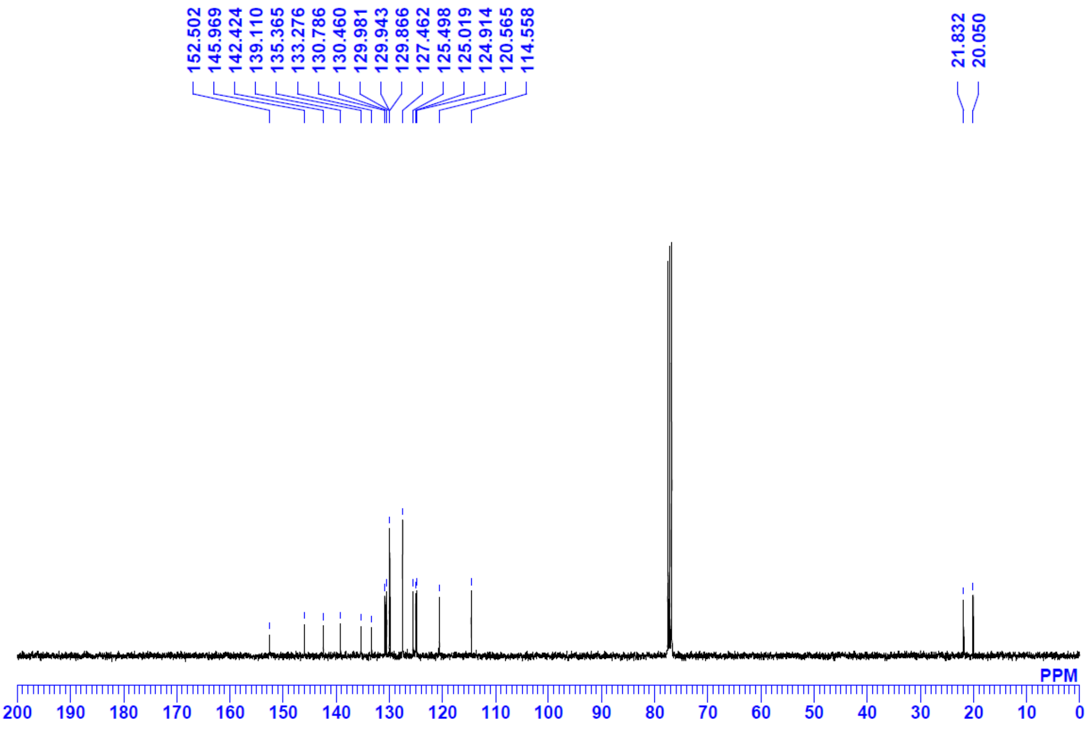

^{1}H NMR of **16f**

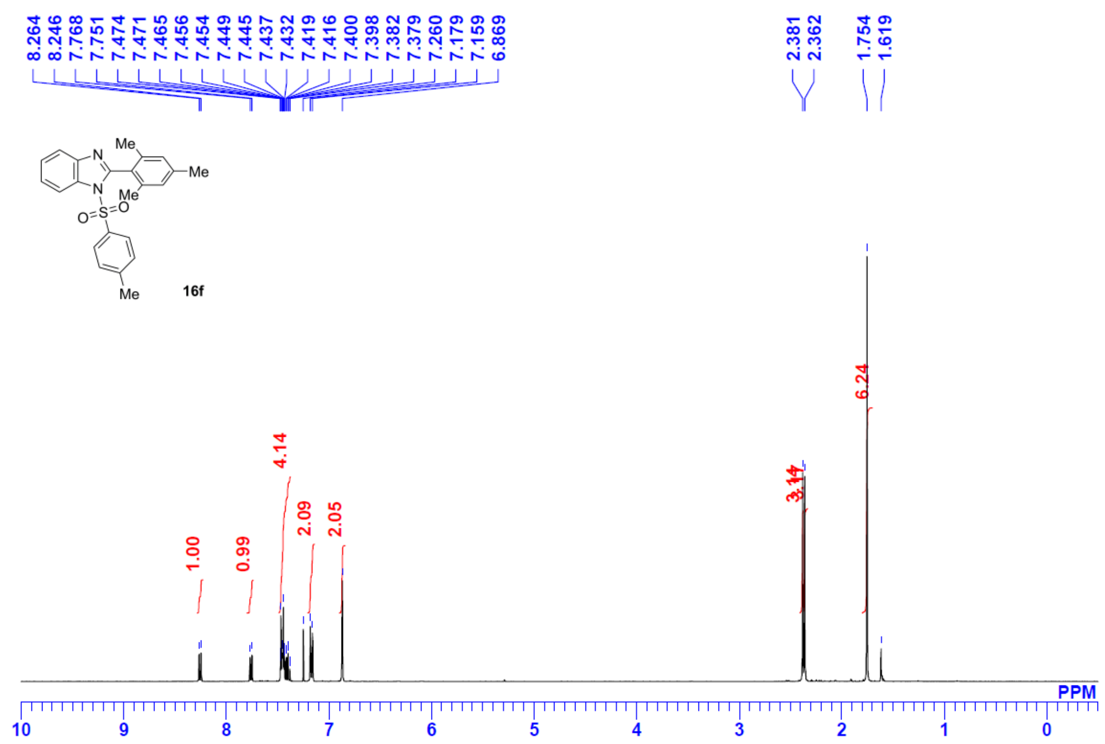

^{13}C NMR of **16f**

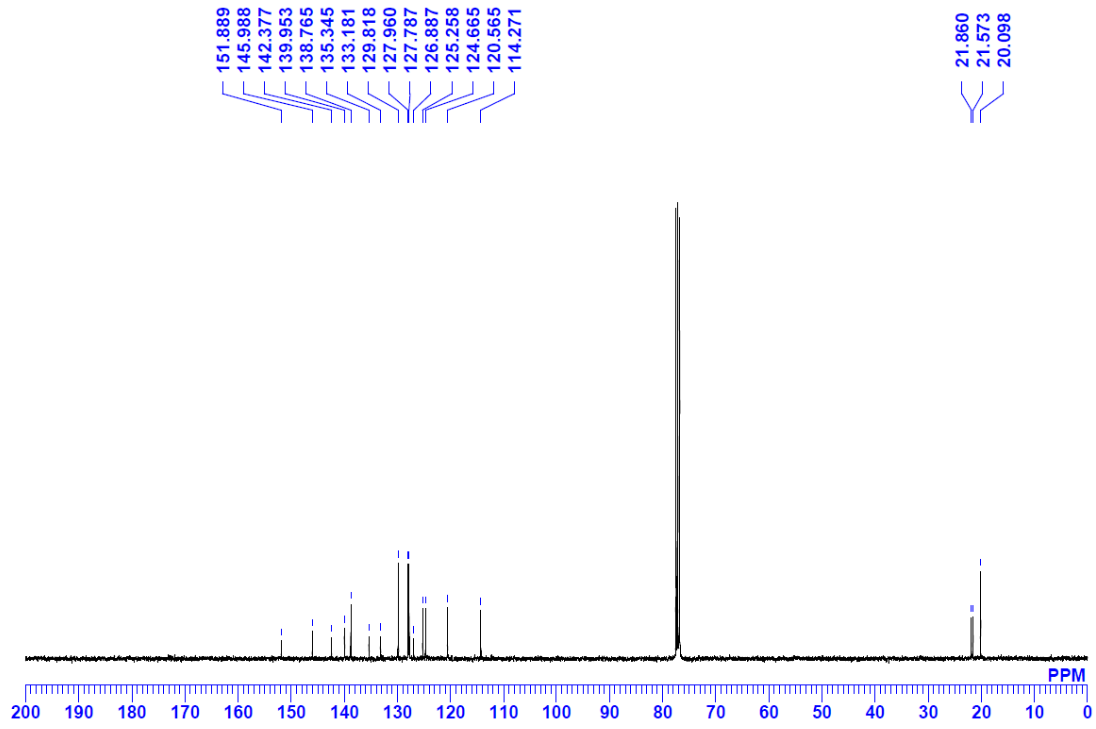

^{1}H NMR of **16g**

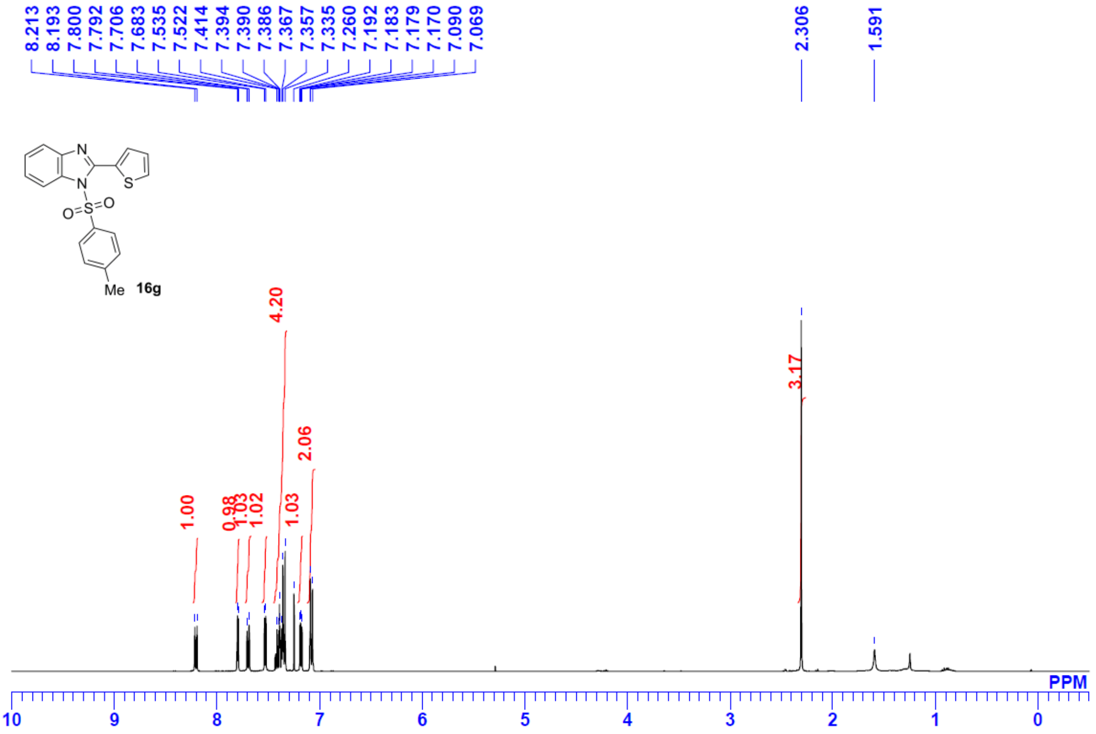

^{13}C NMR of **16g**

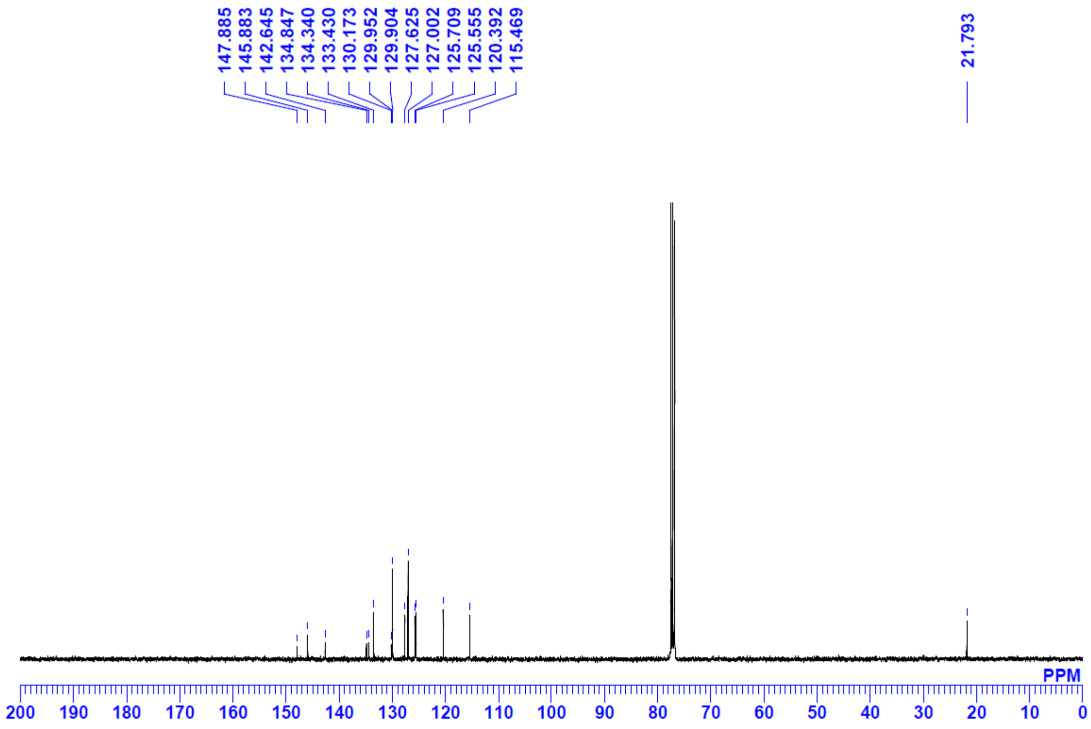

^{1}H NMR of **16h**

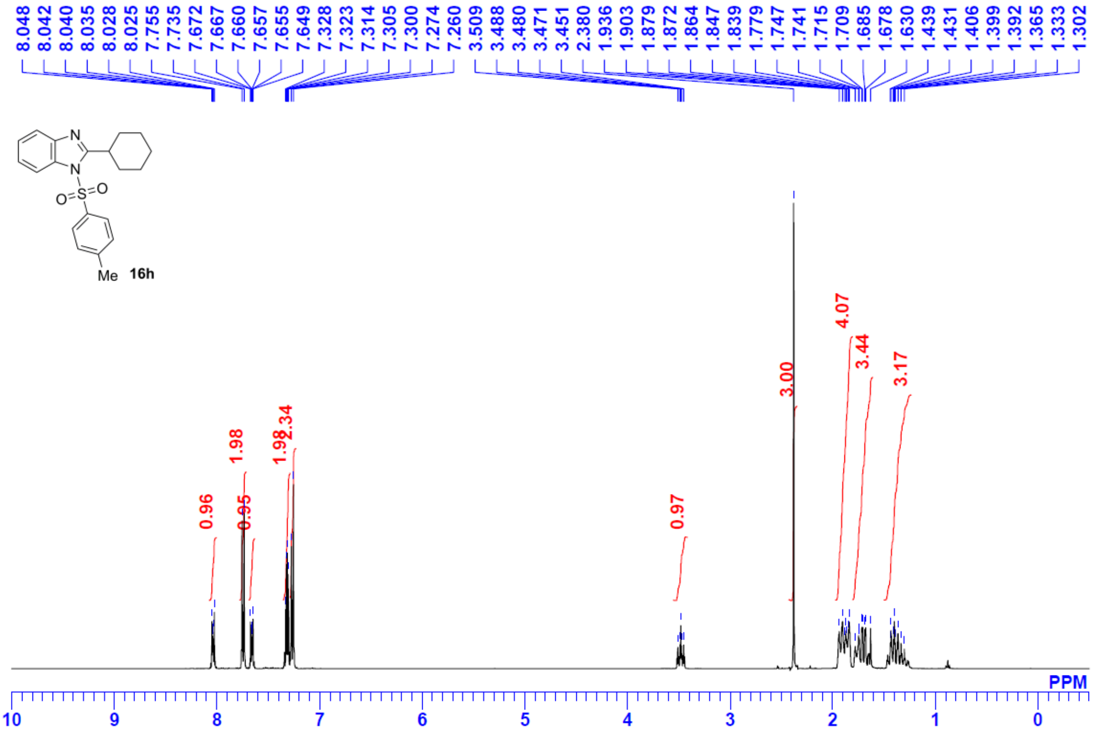

^{13}C NMR of **16h**

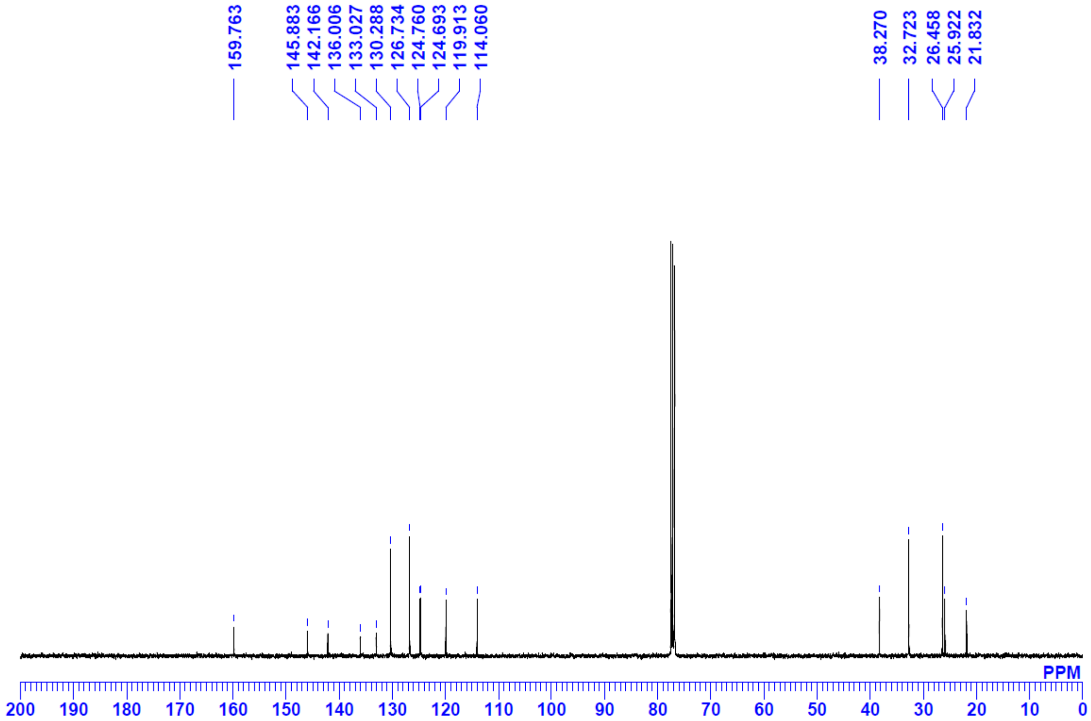

^{1}H NMR of **16i**

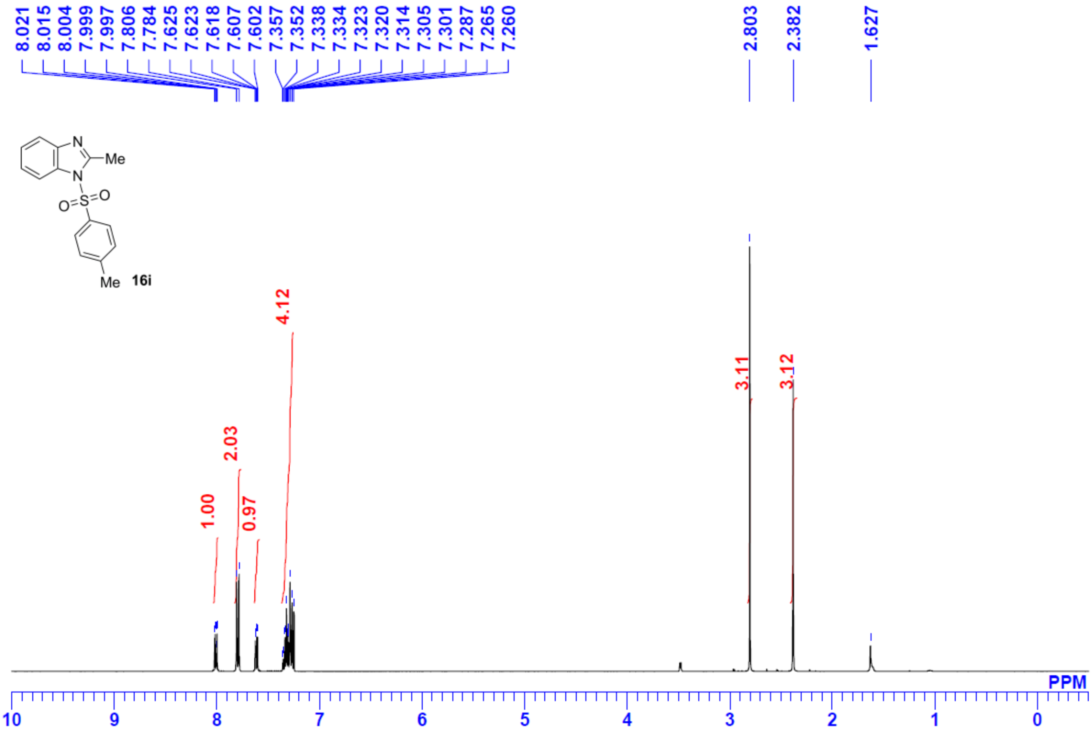

^{13}C NMR of **16i**

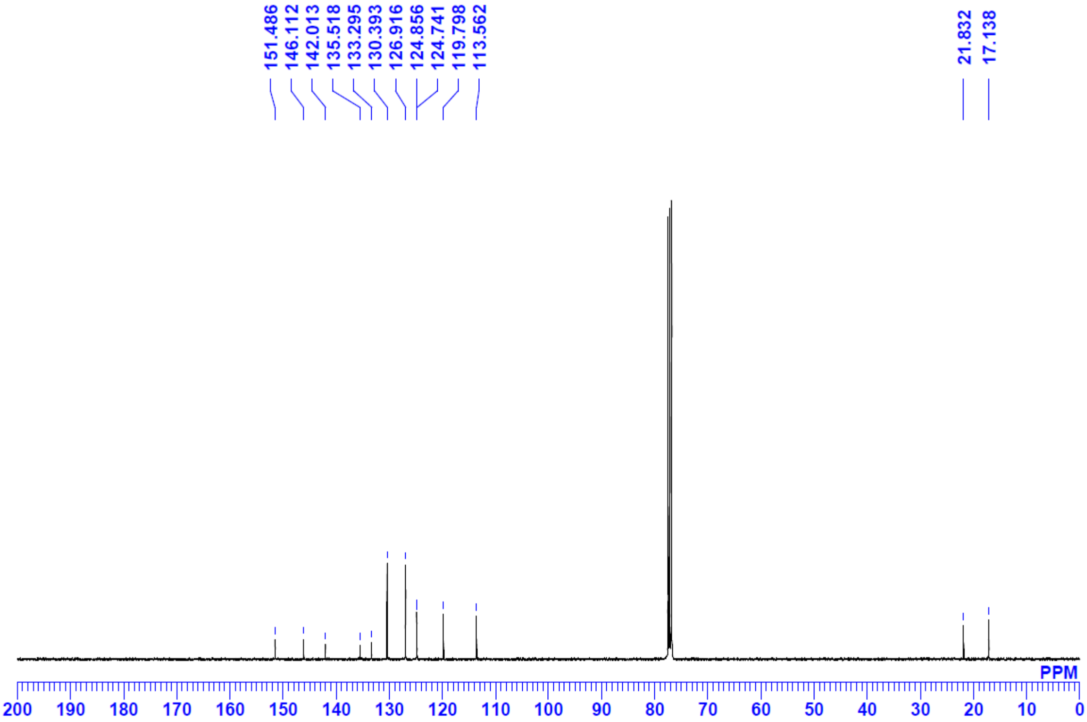

7. NMR Experiments

Given in Figure S1 are results of NMR experiments for reaction mixture (6 h) showing the in situ generation of **17**.

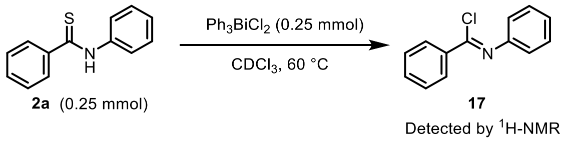

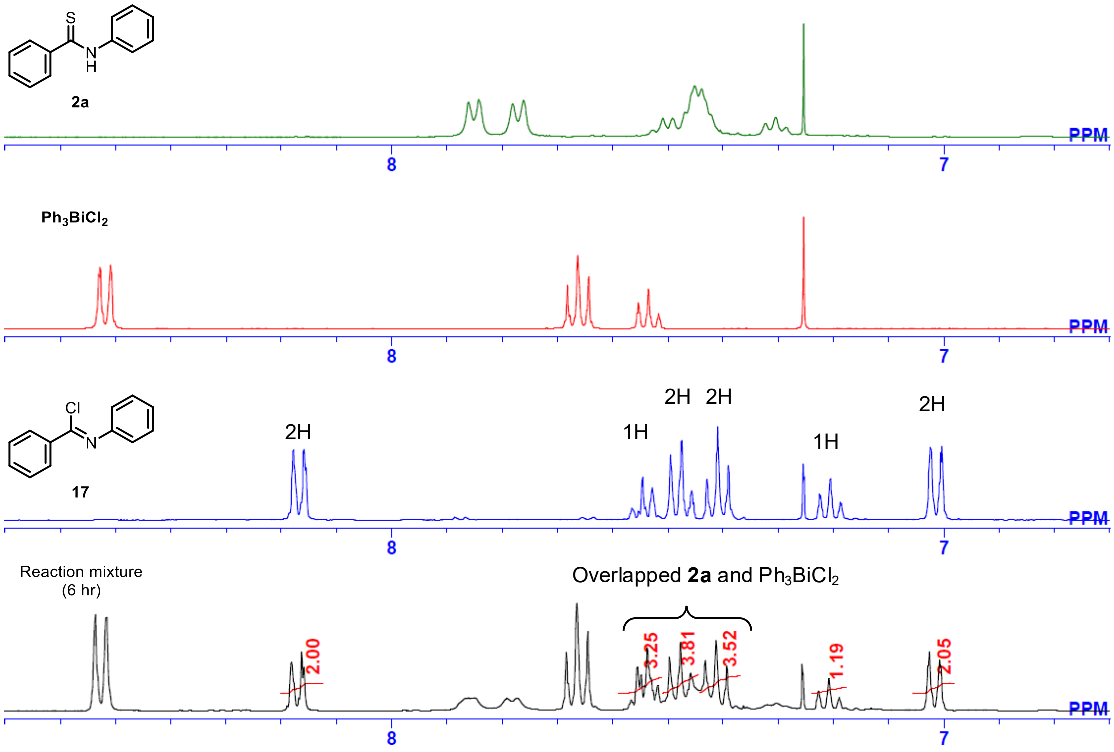

Figure S1. Detected reaction intermediate by ^{1}H NMR in $CDCl_3$ (aromatic region).
